# Interaction of a Densovirus with Glycans of the Peritrophic Matrix Mediates Oral Infection of the Lepidopteran Pest *Spodoptera frugiperda*

**DOI:** 10.3390/v11090870

**Published:** 2019-09-17

**Authors:** Laetitia Pigeyre, Malvina Schatz, Marc Ravallec, Leila Gasmi, Nicolas Nègre, Cécile Clouet, Martial Seveno, Khadija El Koulali, Mathilde Decourcelle, Yann Guerardel, Didier Cot, Thierry Dupressoir, Anne-Sophie Gosselin-Grenet, Mylène Ogliastro

**Affiliations:** 1 Ecole Pratique des Hautes Etudes (EPHE), PSL Research Univ, DGIMI, Univ Montpellier, INRA, 34095 Montpellier, France; laetitia.pigeyre@hotmail.com (L.P.); malvina.schatz@ephe.psl.eu (M.S.); thierry.dupressoir@ephe.psl.eu (T.D.); 2 Diversité des Génomes et Interactions Microorganismes Insectes (DGIMI), Univ Montpellier, INRA, 34095 Montpellier, France; marc.ravallec@inra.fr (M.R.); nicolas.negre@umontpellier.fr (N.N.); cecile.clouet@inra.fr (C.C.); 3 Estructura de Recerca Interdisciplinar en Biotecnologia I Biomedicina (ERI-BIOTECMED, Deaprtment of Genetics Faculty of Biological Sciences Univ Valencia, 46100 Burjassot, Spain; gasmi.leila@gmail.com; 4 BioCampus, Univ Montpellier, CNRS, INSERM, 34000 Montpellier, France; martial.seveno@fpp.cnrs.fr (M.S.); Khadija.El-Koulali@fpp.cnrs.fr (K.E.K.); Mathilde.Decourcelle@fpp.cnrs.fr (M.D.); 5 Unité de Glycobiologie Structurale et Fonctionnelle (UGSF) Univ Lille, CNRS, UMR 8576—UGSF, 59000 Lille, France; yann.guerardel@univ-lille1.fr; 6 Institut Européen des Membranes (IEM), Univ Montpellier, CBRS, ENSCM, 34095 Montpellier, France; didier.cot@umontpellier.fr

**Keywords:** insect, Lepidoptera, insect parvovirus, chitin, peritrophins, glycans, biocontrol

## Abstract

The success of oral infection by viruses depends on their capacity to overcome the gut epithelial barrier of their host to crossing over apical, mucous extracellular matrices. As orally transmitted viruses, densoviruses, are also challenged by the complexity of the insect gut barriers, more specifically by the chitinous peritrophic matrix, that lines and protects the midgut epithelium; how capsids stick to and cross these barriers to reach their final cell destination where replication goes has been poorly studied in insects. Here, we analyzed the early interaction of the *Junonia coenia* densovirus (JcDV) with the midgut barriers of caterpillars from the pest *Spodoptera frugiperda*. Using combination of imaging, biochemical, proteomic and transcriptomic analyses, we examined in vitro, ex vivo and in vivo the early interaction of the capsids with the peritrophic matrix and the consequence of early oral infection on the overall gut function. We show that the JcDV particle rapidly adheres to the peritrophic matrix through interaction with different glycans including chitin and glycoproteins, and that these interactions are necessary for oral infection. Proteomic analyses of JcDV binding proteins of the peritrophic matrix revealed mucins and non-mucins proteins including enzymes already known to act as receptors for several insect pathogens. In addition, we show that JcDV early infection results in an arrest of *N*-Acetylglucosamine secretion and a disruption in the integrity of the peritrophic matrix, which may help viral particles to pass through. Finally, JcDV early infection induces changes in midgut genes expression favoring an increased metabolism including an increased translational activity. These dysregulations probably participate to the overall dysfunction of the gut barrier in the early steps of viral pathogenesis. A better understanding of early steps of densovirus infection process is crucial to build biocontrol strategies against major insect pests.

## 1. Introduction

The transmission of parvoviruses predominantly occurs by horizontal routes through inhalation or oral exposure, making interaction with mucosal epithelia a crucial part of their pathogenesis (for review [1]). The oral route represents a major challenge for viruses as they need to overcome a diversity of barriers to invade their host. Indeed, most animal epithelia are covered in their apical surface by a carbohydrate-rich meshwork of various complexity and thickness, the glycocalyx, which can be coated by an additional layer of secreted mucus [2]. These structures constitute successive protective surfaces where viruses aggregate and either access to attachment factors and receptors at the surface of the epithelial cells or are eliminated by luminal or cilia movements [3]. This dual fate depends on the virus affinity for glycans, which must allow to escape the trap of the mucus. The diversity of glycans present on the epithelial surfaces vary between and within species and therefore constitute an important component of the innate immunity and of the species barrier [4].

Members of the *Parvoviridae* family are non-enveloped viruses that have a simple capsid with T = 1 icosaedral symmetry protecting a 4–6 kb linear, single stranded (ss) DNA genome [5]. They can cause diseases of various severity in a wide range of animals. Parvovirus interest in human and animal health or in biomedicine as vectors for gene transfer, has long focused research to understand their cell tropism and entry mechanisms. A number of cellular attachment factors and receptors have been characterized, mostly for vertebrates’ parvoviruses. They highlight the importance of glycans in capsid recognition and binding specificity [6,7,8]. However, how capsids interact with epithelia remains poorly known, mainly due to difficulties to reconstitute in cellular models the complexity of animal epithelial systems [9,10].

Insect parvoviruses, named densoviruses, can be highly pathogenic, a feature that can both represent threats for insect mass rearing or opportunities for biocontrol against harmful insects as alternative to chemicals. Developing methods against infections or tools for biocontrol requires a deep understanding of the mechanisms driving host range and pathogenesis.

Like their vertebrate counterparts, densoviruses are mainly transmitted orally, gut recognition and binding constitute the primary step of their pathogenesis. The mechanisms determining densovirus specificity are poorly known. Depending on species, densovirus replication can be either restricted to or exclude the gut, viral particles being then transported across the epithelium by transcytosis to reach internal organs where replication goes [11,12,13,14]. Only one cellular receptor has been so far characterized for a “gut restricted” densovirus. It is a mucin of the gut of the silkworm *Bombyx mori*, whose inactivation makes silkworms resistant to infection with the *Bombyx mori* densovirus type 1 (BmDV) [15]. We have previously reported that gut transcytosis of the *Junonia coenia* densovirus (JcDV) involves a gut specific receptor-dependent mechanism in caterpillars [12]. However, the mechanisms used by viral particles to overcome the successive intestinal barriers of different structures and composition remain elusive.

In insects, the intestinal tract is covered by a chitinous acellular layer, which has specific features due to the dual embryonic origin of the gut. Indeed, anterior and posterior extremities of the gut are ectodermal and the acellular layer is covered by an impermeable cuticle. The midgut section is endodermal and has no cuticle but is covered in most insects by a semi-permeable membrane, named the peritrophic matrix (PM) (for review [16]). The PM forms a highly organized lattice of chitin fibrils associated with glycoproteins, mainly peritrophins that have a chitin-binding domain [17,18]. The midgut is thus the portal of entry for most pathogens and their interaction with the PM a critical step of their pathogenesis.

The PM forms pores whose size varies between insect species (e.g., 7–36 nm in Lepidoptera and up to 150 nm in Coleoptera) and developmental stages [19,20]. Large entomopathogenic viruses have developed specific mechanisms to pass through extracellular matrices including virus-encoded enzymes and specific proteins that are associated with the viral particles of baculo-and entomopoxviruses [21,22,23,24]. How densoviruses cope with the physical barriers that constitute the gut and in particular the PM is so far unknow. Due to their small size, it was initially thought they could diffuse passively across the pores of the matrix, but measures of the size of pores, the complexity of the PM and the nature of the interactions between components make this hypothesis unlikely [16,20].

We previously reported that following oral infection, viral particles of JcDV, a type species ofthe *Ambidensovirus* genus, aggregate on the PM of *Spodoptera frugiperda* caterpillars as a first step of the infection process [12]. Such rapid virus concentration on a carbohydrate-rich surface suggested a lectin-like activities of the capsids. Although there is no sequence similarity of the unique protein making the surface of the JcDV with lectin domains, its structure displays similarities with cellular carbohydrate binding proteins including lectins, which suggests that capsids could indeed recognize and bind carbohydrates [25].

Herein, we used a combination of approaches, including microscopy, biochemistry, proteomics and transcriptomics, to decipher the interaction of JcDV with PM major components (i.e., chitin, glycans and proteins). We found that capsids affinity for the PM might result from multiple interactions with different glycans including chitin and glycosylated proteins. In addition, we showed that JcDV early infection results in (i) an arrest of *N*-Acetylglucosamine (GlcNAc) secretion by epithelial cells associated with a disorganization of the PM structure mimicking the effect of chitin-binding plant lectin; (ii) substantial changes in the expression of gut genes, which may also contribute to an early gut dysfunction and participate to viral pathogenesis.

## 2. Materials and Methods

### 2.1. Insect Rearing and Virus Preparation

Caterpillars were reared under controlled conditions (25 ± 1 °C, 65 to 70% relative humidity [RH], 16-h light, 8-h dark photoperiod) on a wheat germ-based artificial diet. JcDV was amplified by oral infection. At death, larvae were crushed and virus extraction was processed by clarification and filtration on 0.22 μm to constitute a semi-purified virus stock (JcDV). To obtain a purified viral stock (P_JcDV), the semi-purified virus stock was loaded on OptiPrep^TM^ (Sigma-Aldrich, Lyon, France) density gradient and dialyzed against Phosphate-buffered saline (PBS) 1X as previously described in [13]. Viral concentrations were estimated by quantitative PCR (qPCR) as described in [12] and expressed as viral equivalent genomes ([veg]). Virus titers were determined by the tissue culture assay method (50% tissue culture infective dose (TCID50) in the permissive Ld652 cells as previously described [26].

### 2.2. Calcofluor, Glycans and Lectins

Calcofluor white M2R (Calcofluor; F3543), *N*-Acetyl-D-glucosamine (GlcNAc; A4106), *N*-Acetyl-D-galactosamine (GalNAc; A2795), D-(+)-Fucose (Fucose; F8150), D-(+)-Mannose (Mannose; M6020), Mucin from porcine stomach (Porcine mucin; M2378) and WGA-FITC conjugate (L4895) were all purchased from Sigma-Aldrich (Lyon, France).

### 2.3. Insect Bioassays

For in vivo bioassays, third-instar (L3) *S. frugiperda* caterpillars were individually infected by feeding with JcDV (10^9^ veg/caterpillar) or by intrahemocelic injection (10^9^ veg/caterpillar). Each treatment was applied to a cohort of 24 caterpillars and three independent experiments were performed. Calcofluor (0.5% to 3%) was concomitantly administrated with the virus to L3 or L6 caterpillars (as described in the Figures). For competition assays, JcDV was incubated with glycans (GlcNAc, GalNAc, Fucose or Mannose at 5 μM and/or 5 mM; for one hour before feeding or injection. In all experiments, control larvae were fed or injected with PBS. Caterpillars mortality was recorded each day during 10 days and results were presented as survival rates per day. The time to death was assessed by comparing the survival curves using the Kaplan Meier method (GraphPad Prism software, version 7). The significance between groups were analyzed using log-rank (Mantel-cox) tests and Gehan-Breslow-Wilcoxon tests.

### 2.4. Hemagglutination Assay

Rabbit erythrocytes cells were diluted at 3% (*v*/*v*) with 150 mM NaCl and treated with 0.5 mg/mL of trypsin (Sigma) for 30 min at 37 °C. After 3 washes with 150 mM NaCl, 25 μL serially two-fold dilutions of viral inocula (JcDV or P_JcDV; started from 10^11^ veg/μL) were mixed with 25 μL of erythrocytes in 96 well microtiter plates for 30 min at 37 °C. Positive controls of hemagglutination was performed using 25 μL of WGA (1 mg/mL). Fifty μL of erythrocytes were subsequently added to each well for 30 min at 37 °C. Hemagglutination was read under a light microscope.

### 2.5. PM Isolation, Ex Vivo Infection and Microscopic Observation

For Calcofluor experiments, PMs were isolated from sixth-instar (L6) *S. frugiperda* caterpillars, previously anesthetized on ice, then opened and washed with Phosphate Buffered Saline (PBS) to remove the food bolus. PMs isolated from caterpillars treated with Calcofluor were fixed 1 h with 4% paraformaldehyde (PFA), then dehydrated with increasing concentrations of ethanol (50% to 100%) and in 1:1 (100% ethanol and hexamethyldisilazane [HMDS]) for 10 min and finally in 100% HMDS for 20 min. After overnight evaporation, samples were ultimately coated with Platinium and observed with a Scanning Electron Microscope (SEM) HITACHI S-4800, with an acceleration voltage of 2 kV and a working distance of 5 mm.

For ex vivo infection experiments, PMs were isolated as described above and incubated for 30 min at room temperature (RT) with JcDV (10^9^ veg/μL; 500μL), or with JcDV pre-incubated for 1 h with glycans (GlcNAc, GalNAc, Fucose or Mannose; 5 μM to 5 mM) before infection. The PMs were then washed with PBS and fixed with 4% PFA as above. Immunolabelling of JcDV particles was performed using a specific rabbit anti-capsid antibody (1:1,000; Eurogentec) incubated for 2 h at RT and an anti-rabbit secondary antibody (Alexa Fluor^®^ 568; 1:500; Invitrogen, Carlsbad, CA, USA) incubated for 45 min at RT. Labeled PMs were then mounted in Dako (Sigma) on coverslips for observation. For epifluorescence measurements, we worked on a Zeiss AxioImager Z2, equipped with suitable filters for the dyes, using a 40×/1.3 Plan-Apochromat Oil PH3 and a 63×/1.4 Plan-Apochromat Oil PH3 objectives. For structured illumination images, the Zeiss ApoTome-slider was introduced into the field-stop plane of the microscope to improve resolution of images. We used the ZEN software to operate the microscope and took at least 10 images (10 fields per PM) for each treatment. All images were taken with a CMOS Orca Flash 4.0 B&W camera. Images were processed with Fiji software. The intensity of fluorescence (arbitrary unit) were measured on epifluorescence images. Statistical analyses were performed using the non-parametric Kruskal-Wallis test (GraphPad Prism Software, version 7, GraphPad Software, San Diego, CA, USA). Each experiment was repeated at least three times, and each independent experiment gave similar results.

### 2.6. Midgut Semi-Thin Sections and Immunolabelling

L3 *S. frugiperda* caterpillars were individually infected by feeding as in Section 2.3. Twenty four hours later, caterpillars were anesthetized on ice and sacrificed. In parallel, caterpillars were kept until death to insure their infection status. Sacrificed caterpillars were fixed in 4% PFA + 2.5% glutaraldehyde for 1h at 4 °C and dehydrated by incubations in increasing concentrations of ethanol (50% to 100%) before progressive embedding in 100% Unicryl resin (BB International) as described in [12]. Semi-thin sections (1 μm) were cut in the central part of the caterpillars corresponding to the midgut. After 45 min of incubation with WGA-FITC (1:300), to label GlcNAc and the PM, or with Phalloidin-FITC (1:300; Sigma), to label actin cytoskeleton, and 10 min with Dapi (1 μg/mL; Invitrogen) to label nuclei, semi-thin sections were mounted and observed with a Zeiss AxioImager Z2 microscope using the structured illumination module Zeiss ApoTome-slider as in 4.5. Images were taken with a CMOS Orca Flash 4.0 B&W camera using fixed parameters for all treatments and processed with Fiji software.

### 2.7. Chitin Binding Assay and Pull Down

Ten μL of chitin beads (New England Biolab) were washed three times with PBS and added in 200 μL of virus suspension (10^9^ veg/μL in PBS). After 1 h of incubation with gentle rotation, beads were pulled-down by centrifugation (500× *g* for 5 min à 4 °C), washed five times with PBS, then resuspended in Laemmli buffer 2 × (4% SDS, 20% glycerol, 10% 2-mercaptoethanol, 0.004% bromophenol blue and 0.125 M Tris HCl, pH 7) and heated at 95 °C for 5 min before western blot analysis. Two μL (5% of the pull down and 1% of the initial input) was loaded on 4–15% polyacrylamide Tris-HCl gel (Mini-PROTEAN^®^ TGX™ Precast Gels, Biorad, Hercules, CA, USA) and separated by SDS-PAGE for 1 h at 150 V. Next, samples were transferred to PVDF membrane (Immobilon-P, Merck) for 2 h at 200 mA. Subsequently, the membrane was saturated with 5% of milk in PBS/Tween 0.1% (PBST), then incubated 2 h at RT with the primary anti-capsid antibody (1:1,000; see above). After washes in PBST, the membrane was incubated 1 h at RT with an anti-rabbit secondary antibody HRP-conjugated (1:3000; Biorad, Hercules, CA, USA). Proteins were revealed by enhanced chemiluminescence (Millipore, Burlington, MA, USA) using a Chemidoc imager (Biorad).

### 2.8. SDS-PAGE, PAS Staining and VOPBA

Peritrophins were extracted from pools of five PMs from sixth instar *S. frugiperda* in 200 μL of Laemmli buffer 2X, then boiled and heated at 95 °C for 5 min [27]. After centrifugation at 8000× *g* for 10 min 4 °C, the supernatant was collected and 1/15 was loaded on 4–15% polyacrylamide Tris-HCl gel and separated by SDS-PAGE as above. Thirty μg of porcine mucins were also loaded on the same gel. Gel was stained with Page blue (Thermo Fisher Scientific, Waltham, MA, USA) to analyze total proteins or with Periodic Acid-Schiff (PAS) as described in [28] to analyze glycosylated proteins. Proteins were also transferred on nitrocellulose membranes (Biorad) for 3 h at 70 V for viral protein overlay binding assay [29]. Briefly, the membrane was saturated with 3% BSA in PBST for 2 h at RT, then incubated OVN at 4 °C with JcDV (10^9^ veg diluted in PBST containing 1% BSA). After washes in PBST, the membrane was incubated with the rabbit anti-capsid antibody (1:1000; see above), then the anti-rabbit secondary antibody HRP-conjugated, and proteins were revealed by enhanced chemiluminescence as above.

### 2.9. Proteomic LC-MS/MS Analysis and Data Processing

Protein bands revealed by VOPBA were cut in SDS-PAGE gel stained with Page blue (3 replicates, 10 bands) and destained with three washes in 50% acetonitrile and 50 mM triethylammonium bicarbonate (TEABC). After protein reduction (with 10 mM dithiothreitol in 50 mM TEABC at 60 °C for 30 min in the dark) and alkylation (55 mM iodoacetamide TEABC at room temperature for 30 min) proteins were digested in-gel using trypsin (0.5 μg/band, Gold, Promega, Madison, WI, USA) as previously described [30]. Digested products were dehydrated in a vacuum centrifuge and resuspended with 0.05% Trifluoroacetic acid (TFA) and 2% ACN. The generated peptides were analyzed online by nano-flow HPLC–nanoelectrospray ionization using a Q-Exactive Plus mass spectrometer (Thermo Fisher Scientific, Waltham, MA, USA) coupled to an Ultimate 3000 RSLC (Thermo Fisher Scientific). Desalting and pre-concentration of samples were performed on-line on a Pepmap^®^ pre-column (0.3 mm × 10 mm, Dionex, Sunnyvale, CA, USA). The capillary reverse-phase column (0.075 mm × 150 mm, Acclaim Pepmap 100^®^ C18, Thermo Fisher Scientific) fitted with an uncoated silica PicoTip Emitter (New Objective, Woburn, MA, USA) was first equilibrated in solvent A (0.1% formic acid) and a multistep linear gradient of acetonitrile consisting of 0–25% of solvent B (0.1% formic acid in 80% acetonitrile) for 30 min, 25–40% for 5 min and 90% for 8 min, at 300 nL/min was used to elute peptides from. Spectra were acquired with the instrument operating in the information-dependent acquisition mode throughout the HPLC gradient. Survey scans were acquired in the Orbitrap system with resolution set at a value of 70,000. Up to twelve of the most intense ions per cycle were fragmented and analyzed using a resolution of 17,500. Peptide fragmentation was performed using nitrogen gas on the most abundant and at least doubly charged ions detected in the initial MS scan and an active exclusion time of 45 s. For all full scan measurements with the Orbitrap detector a lock-mass ion from ambient air (*m*/*z* 445.120024) was used as an internal calibrant as described [31].

Analysis was performed using the MaxQuant software (version 1.5.5.1) [32]. All MS/MS spectra were searched using Andromeda against a decoy database consisting of a combination of *S. frugiperda* databases [33] and 236 classical contaminants, containing forward and reverse entities. The following settings were applied: Spectra were searched with a mass tolerance of 7 ppm (MS) and 0.5 Th (MS/MS). Enzyme specificity was set to trypsin. Up to two missed cleavages were allowed and only peptides with at least seven amino acids in length were considered. Carbamidomethylation was set as fixed cystein modification and oxidation was set as variable methionine modification for searches. FDR was set at 0.01 for peptides and proteins. Sequences which found homology were annotated according to the gene ontology (GO) terms and classified using Blast2Go software (https://www.blast2go.com/; [34]). The enrichment in GO terms compared to the *S. frugiperda* reference (predicted proteins from the OGS2.2 genome; Gouin et al., 2017) was analyzed with the same software (FDR set at 0.01).

### 2.10. RNA Extraction, DGE Library Construction and Sequencing

Fourth instar *S. frugiperda* caterpillars were orally infected or not with JcDV (10^12^ veg per caterpillar; Twenty caterpillars per condition). At 1-day p.i. or 3 days p.i., caterpillars were anesthetized on ice and dissected. The midguts were washed in PBS to eliminate the food bolus and the PMs. Trachea, Malpighi tubes and visceral muscles were removed and the epithelia were incubated with 2.5% trypsin for 30 min to dissociate the tissues. After washes, gut cells were lysed in 800 μL of TRIzol^®^ reagent (Invitrogen) for total RNA extraction according to the manufacturer’s instructions. Total RNA amount and purity were checked by using spectrophotometrer NanoDrop ND-1000 (Thermo Scientific) and the integrity of total RNA was analyzed by capillary electrophoresis (2100 Bioanalyzer Instrument, Agilent, Santa Clara, CA, USA).

We used Digital Gene Expression (DGE) method that generates short sequences (Tags) specific for mRNA [35,36,37]. Four DGE libraries were constructed from midgut total RNA extracted from *S. frugiperda* caterpillars infected (or not) for 1 and 3 days. Sequence tag preparation was done with Illumina’s Digital Gene Expression Tag Profiling Kit according to the manufacturer’s protocol (version 2.1B) as described in [36]. For fourth libraries, 10 μg of total RNA were incubated with oligo-dT beads. First-and second-strand cDNA syntheses were performed using superscript II reverse transcription kit according to the manufacturer’s instructions (Invitrogen). The cDNAs were cleaved using the NlaIII anchoring enzyme. Subsequently, digested cDNAs were ligated with the GEX adapter 1 containing a restriction site for MmEI. The second digestion with MmeI was performed, which cuts 17 bp downstream of the CATG site. At this point, the fragments detach from the beads. The GEX adapter 2 was ligated to the 3′ end of the tag. A PCR amplification with 15 cycles using Phusion polymerase (Finnzymes) was performed with primers complementary to the adapter sequences to enrich the samples for the desired fragments. The resulting fragments of 85 bp were purified by excision from a 6% polyacrylamide TBE gel. The DNA was eluted from the gel using Spin-X Cellulose Acetate Filter (0.45 μm), precipitated, resuspended in 10 mM Tris-HCl (pH 8.5) and quantified using Nanodrop 1000 spectrophotometer. Cluster generation was performed after applying 4 pM of each sample to the individual lanes of the Illumina 1G flowcell. After hybridization of the sequencing primer to the single-stranded products, 18 cycles of base incorporation were carried out on the 1G analyzer according to the manufacturer’s instructions. Image analysis and base calling were performed using the Illumina Pipeline, where sequence tags were obtained after purity filtering. We could assign 63% of the tags (Supplementary Materials Tables S2–S4) out of which 54% correspond to multiple matches and were discarded from functional analysis with GO.

### 2.11. Transcriptomic Analysis of the Midgut

Functional annotation was performed using BIOTAG software (Skuld-Tech, Grabels, France). The statistical value of DGE data comparisons, as a function of tag counts, was calculated by assuming that each tag has an equal chance of being detected. Differential expression of the Tag counts of the infected vs. mock conditions was performed to obtain a list of up- and down-regulated tags for each condition. Tags for which differential expression was ≥ 5 fold change were assigned using the reference databases for *S. frugiperda* [33,38]. Sequences with homology were annotated according to the GO terms and classified using Blast2Go software (https://www.blast2go.com/; [34]) and represented at level 2 of Biological process and Molecular function. The enrichment in GO terms compared to the *S. frugiperda* reference (predicted transcripts from the OGS2.2 genome) was analyzed with the same software (FDR set at 0.01). The Junonia coenia densovirus (former JcDNV) corresponds to the complete genome of the Oxford isolate (Genbank accession number KC883978.1).

## 3. Results

### 3.1. The Peritrophic Matrix of S. frugiperda is a Barrier to JcDV Infection

**Figure 1:**
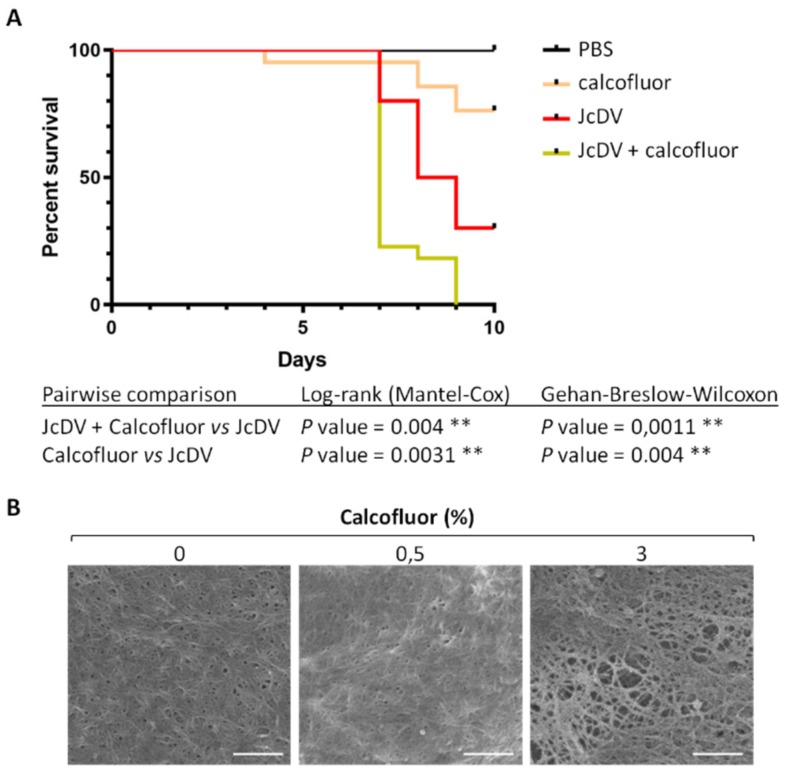
Calcofluor increases *S. frugiperda* caterpillar’s susceptibility to densovirus oral infection. (**A**) Survival of *S. frugiperda* caterpillars following JcDV infection. Larvae (n = 24) were infected individually by feeding with JcDV (10^9^ veg/caterpillar) concomitantly with 0.5% (5 μg) of Calcofluor or PBS as a control. Caterpillar mortality was recorded once a day during 10 days and results were presented as survival rates per day. Three independent experiments were performed, each independent experiment gave similar results, one is represented here. The log-rank (Mantel-cox) and the Gehan-Breslow-Wilcoxon tests were used to determine statistical significance. *p* Values of less than 0.05 were considered significant (**, *p* < 0.01). PBS refers to control (PBS-treated and non-infected) caterpillars; Calcofluor refers to Calcofluor-treated and non-infected caterpillars; JcDV refers to JcDV-infected caterpillars and JcDV+Calcofluor to Calcofluor-treated and JcDV-infected caterpillars. (**B**) Calcofluor disrupts the PM integrity. SEM images of PM ultrastructure isolated from L6 caterpillars fed with PBS (0%), 0.5% (5 μg) or 3% (30 μg) of Calcofluor. Endoperitrophic face is shown (bars, 300 nm).

Early studies have estimated that the average pore size of the PM is around 8 nm in *S. frugiperda* caterpillars, which might exclude 25 nm densovirus particles [39]. To assess the PM barrier function against densovirus infection, we disrupted its structure by feeding third instar (L3) larvae with sub lethal doses (0.5%) of the chitin-binding agent Calcofluor White prior JcDV infection [40,41,42,43,44]. We then measured larval mortality rates daily. Results showed that JcDV infected larvae pre-treated with Calcofluor displayed a significant shorter median time to death (LT50) compared to untreated infected larvae (7 vs. 9 days p.i. for control larvae; *p* < 0.01) (Figure 1A and Supplementary Materials Figure S1), supporting that the PM can limit JcDV infection. Noteworthy, an increased larval mortality was also observed at late time post-treatment with Calcofluor in mock-infected caterpillars compared to untreated controls (18% at 10 days p.i.), confirming a detrimental inhibition of chitin assembly on larval development [41].

We analyzed the effect of Calcofluor on the PM integrity by scanning electron microscopy (SEM). Because the PM of L3 larvae has a gel-like structure that cannot be manipulated, we took L6 larvae for this experiment as the PM is thick and solid at this stage and can be easily dissected. L6 caterpillars were fed with up to 3% Calcofluor (not lethal at 24 h post treatment) and PMs were isolated 24 h post-treatment and prepared for SEM analysis. As shown by Figure 1B, PMs from control larvae displayed a highly organized structure, similar to PMs from caterpillars treated with 0.5% Calcofluor. By contrast, PMs from caterpillar fed with 3% Calcofluor had a clear disrupted structure with enlarged pores, confirming that Calcofluor binding to chitin fibrils compromised the integrity of the matrix.

### 3.2. JcDV Binding to the PM is Required for Oral Infection of S. frugiperda Caterpillars

**Figure 2:**
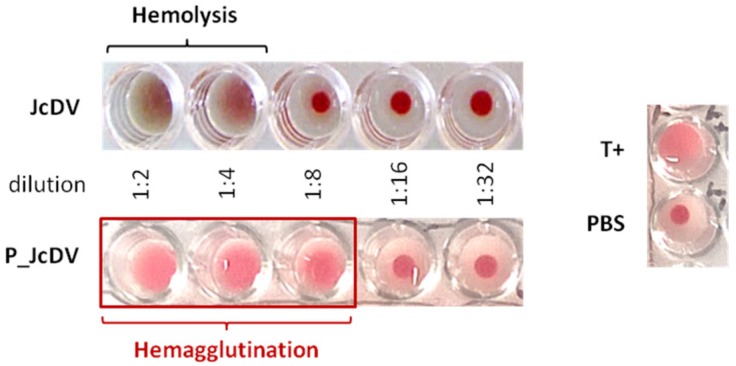
Hemagglutination assays with JcDV. Rabbit erythrocytes cells were incubated with serially two-fold dilutions of JcDV or P_JcDV (from 10^11^ veg/μL, 25 μL/well) for 30 min at 37 °C. Positive control (T+) of hemagglutination was performed using 25 μL of Wheat Germ Agglutin (WGA; 1 mg/mL), negative control (PBS) with 25 μL of PBS instead of virus dilution. Semi-purified virus (JcDV) induced a green coloration in the wells, as a result of hemolysis of erythrocytes. The wells containing purified virus (P_JcDV) showed a clear hemagglutination at the lowest dilutions (1:2 to 1:8).

The rapid recognition of the PM by JcDV capsids suggests that their affinity for glycans is important for the oral infection process. To test this hypothesis, we first assayed the capsid ability to agglutinate erythrocytes, a feature displayed by vertebrate parvoviruses [45,46]. We performed a typical hemagglutination assay, adding serial dilutions of the virus inoculum to rabbit erythrocytes (Figure 2). The first dilutions (1:2 and 1:4) of JcDV triggered a strong hemolysis of erythrocytes suggesting a toxic effect of the viral inoculum, ie capsids or some host-derived component associated with the inoculum. It is worthy to note that we use semi-purified inoculum as it mimics naturally occurring infections. JcDV was therefore further purified on a density gradient (P_JcDV) and similarly assayed for hemagglutination. A clear hemagglutination was obtained with P_JcDV, supporting that toxicity is likely due to a host-derived component that can be eliminated during the purification process. Hemagglutination with P_JcDV was obtained up to the third dilution (hemagglutination titer of 1:8), which indicates a rather weak interaction of the capsids with glycans at the surface of (mammalian) erythrocytes.

**Figure 3:**
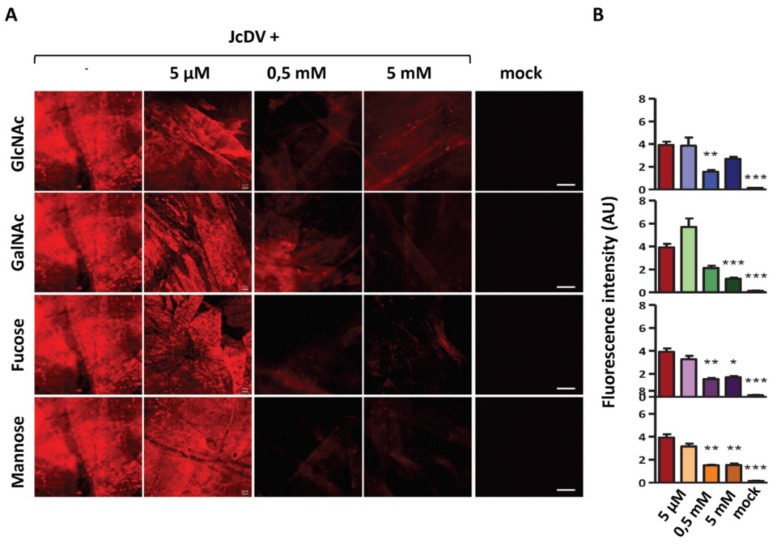
Ex vivo interaction of JcDV with the PM involves carbohydrates. (**A**) Apotome images of isolated PMs incubated with JcDV (5.10^11^ veg/PM) alone or with JcDV treated for 1 h with 5 μM to 5 mM of glycans (GlcNAc, GalNAc, Fucose or Mannose) before incubation. JcDV labelling is in red. Control PMs (mock) were incubated with a clarified and filtered homogenate of non-infected caterpillars. Bars, 50 μm. Each experiment was repeated at least three times, and each independent experiment gave similar results, one is shown here. (**B**) Relative quantification of JcDV binding on isolated PM and competition assays with monomeric glycans. Intensity of red fluorescence (arbitrary unit A.U) of PMs were measured on epifluorescence images (10 images from 3 independent experiments). Statistical analyses were determined using the non-parametric Kruskal-Wallis test. *p* Values of less than 0.05 were considered significant (* *p* < 0.05; ** *p* < 0.01; *** *p* < 0.001).

To better understand capsid affinity for glycans, we performed a competition bioassay using monomeric glycans as JcDV-binding competitors. JcDV binding was revealed with an immunofluorescence staining on the PMs using a specific anti-capsid antibody. We quantified this fluorescence as a proxy of binding and competition. We first performed competition ex vivo on isolated PMs incubated with JcDV in the presence of four monosaccharides commonly found in insects [47], ie *N*-Acetyl-D-glucosamine (GlcNAc), which is the monomeric unit of chitin, *N*-Acetyl-D-galactosamine (GalNAc), D-Fucose and D-Mannose (Figure 3). We first verified with a dot blot assay that capsids interaction with monosaccharides were not interfering with antibody recognition, which validated the competition bioassay (Supplementary Materials Figure S2). As shown in Figure 3, JcDV binding resulted in an intense fluorescence signal on the PMs (left panel), which was similarly competed away by the four monosacharides and within a similar concentration range (0.5 mM to 5 mM). We noted that fluorescence quantification did not result in a strictly linear dose-dependent effect.

**Figure 4:**
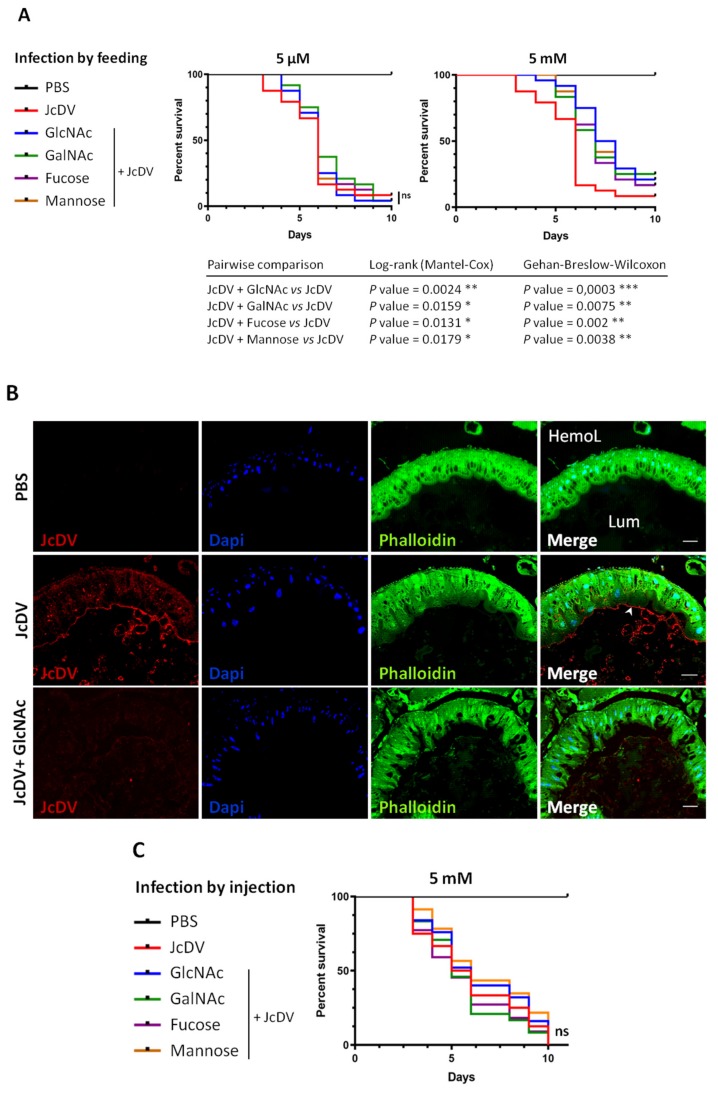
Affinity for glycans mediates JcDV oral infection of *S. frugiperda* caterpillars. (**A**) Survival curves of caterpillars (n = 24) infected by feeding with JcDV alone or with JcDV (10^9^ veg/caterpillar) incubated for 1 h with 5 μM or 5 mM of glycans (GlcNAc, GalNAc, Fucose or Mannose) before infection. Control caterpillars were fed with PBS. Three independent experiments were performed, each independent experiment gave similar results, one is represented here. The log-rank (Mantel-cox) and the Gehan-Breslow-Wilcoxon tests were used to determine statistical significance. *p* Values of less than 0.05 were considered significant (ns, non-significant; * *p* < 0.05; ** *p* < 0.01; *** *p* < 0.001). (**B**) Immunolabeling of midgut semithin transversal sections 2 h after ingestion of JcDV alone or JcDV (10^9^ veg/caterpillar) incubated for 1 h with 5 mM of GlcNAc before oral infection. Control caterpillars were fed with PBS. The PM is shown by an arrowhead. Phalloidin-FITC is in green, JcDV is in red, and nuclei are labeled with Dapi (blue). Bars, 30 μm. Lum, midgut lumen; HemoL, hemolymphatic compartment. (**C**) Survival curves of caterpillars (n = 24) infected by injection of JcDV alone (10^9^ veg/caterpillar) or of JcDV incubated for 1 h with 5 mM of each glycan (GlcNAc, GalNAc, Fucose or Mannose) before infection. Control caterpillars were injected with PBS. Three independent experiments were performed, each independent experiment gave similar results, one is represented here. The log-rank (Mantel-cox) and the Gehan-Breslow-Wilcoxon tests were used to determine statistical significance, *p* > 0.05 were considered non-significant (ns). PBS refers to control (PBS-treated and non-infected) caterpillars; ‘JcDV’ to JcDV-infected caterpillars; ‘JcDV + GlcNAc’, ‘JcDV + GalNAc’, ‘Jc + Fucose’ and ‘Jc + Mannose’ refer to caterpillars infected with JcDV incubated with GlcNAc, GalNAc, Fucose or Mannose, respectively, before infection.

To further test the role of glycans in JcDV pathogenesis, we carried out these competition bioassays in vivo (Figure 4 and Supplementary Materials Figure S3). We mixed JcDV with each monosaccharide prior infection, fed caterpillars with these inocula and calculated mortality rates daily as in Figure 1. Results showed that oral infection with 5 mM (but not 5 μM) of each monosaccharide significantly delayed the median time to death (LT50) of caterpillars (7 vs. 6 days; *p* < 0.05 for 5 mM) (Figure 4A and Supplementary Materials Figure S3A,B), further supporting that PM recognition is the first step of JcDV oral infection. To confirm these results and reveal JcDV binding and competition for binding the PM in vivo, we carried out midgut semi-thin sections and immunofluorescence as above. Caterpillars were infected with JcDV mixed or not with 5 mM GlcNAc and sacrificed at 2 h p.i. for midgut isolation and preparation. As shown in Figure 4B, we observed a red fluorescence signal in untreated infected caterpillars that typically lines the PM. In addition, labelling was also observed in the lumen, likely revealing JcDV interaction with food bolus and/or microbial components. Both signals were strongly and specifically decreased following competition with GlcNAc (Figure 4B), showing that different GlcNAc-containing glycans in the gut lumen can recognize the capsids.

Last, we studied whether such “stickiness” was specifically required by the densovirus to cross the gut, i.e., for oral infection. JcDV infection of target cells (eg epidermis, trachea, hemocytes) proceeds by a receptor-dependent mechanism different from intestinal cells [12]. These cells express glycan structures of various complexity that are attached to the cell surface or secreted and forming extracellular matrices, whose glycans might be similarly targeted by JcDV for attachment. We performed competition bioassays in vivo, bypassing the midgut by injecting caterpillars with JcDV mixed or not with 5 mM of each monosaccharide. Interestingly, the median time to death was similar for all conditions (Figure 4C and Supplementary Materials Figure S3C; *p* > 0.05), showing that none of the monosaccharides competed with JcDV infection proceeding by the systemic route.

Altogether these results show that JcDV capsid is a carbohydrate-binding protein and this feature is required for oral infection to target the PM of caterpillars.

### 3.3. JcDV Binds to Both Chitin and Protein Components of the PM

**Figure 5:**
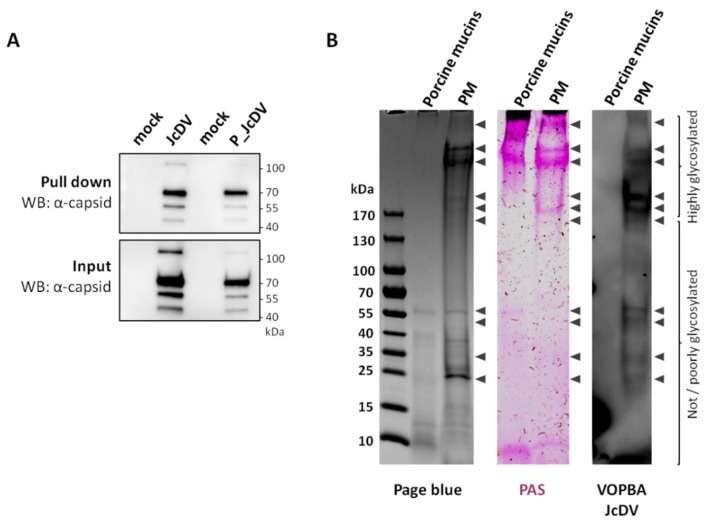
JcDV interacts with chitin and has specific affinity for proteins of the PM. (**A**) Western blot analysis of the chitin binding assay. Chitin beads were incubated with JcDV or P_JcDV. Control binding assays were performed with non-infected homogenate of caterpillars (for JcDV) or with PBS (for P_JcDV). Five % of the pull down and 1% of the input (before pull down) were analysed by SDS-PAGE followed by western blotting using a polyclonal anti-JcDV capsid antibody. The four VP capsids proteins (theoretical molecular weights are VP1, 89 kDa; VP2, 58 kDa; VP3, 53 kDa and VP4, 47 kDa) are detected, with a better detection of VP2 [48]. (**B**) VOPBA analysis of PM proteins interacting with JcDV. Proteins were extracted from PMs and separated by SDS-PAGE as described in Methods. Thirty μg of porcine mucins were also loaded in the gel as a control of highly O-glycosylated proteins. Proteins were then stained with Page Blue or Periodic Acid Schiff (PAS, pink) to visualize total or glycosylated proteins, respectively, and transferred to nitrocellulose membranes for probing with JcDV and anti-JcDV capsid antibody. Proteins interacting with JcDV capsids were finally revealed by enhanced chemiluminescence (black arrowheads on the VOPBA JcDV membrane); the corresponding positions of these bands were reported on the Page blue and PAS gels and indicated as well by black arrowheads on the right of these gels.

We next wanted to determine which component of the PM, i.e., chitin and/or glycosylated proteins were involved in capsid interactions, using biochemical assays. We first tested capsid physical interaction with chitin using a pull-down assay with chitin beads. JcDV from purified or semi-purified inocula were incubated with chitin beads, pulled-down by centrifugation and subsequently revealed by Western blot using a specific JcDV anti-capsid antibody. Figure 5A shows JcDV pull-down by chitin beads and we did not observed difference between inocula (purified vs. semi-purified), which confirmed that capsids can interact directly with chitin.

Second, we tested virus interaction with PM proteins using a Viral Overlay Binding Assay (VOBPA). Total proteins were extracted from isolated PMs, separated with SDS-PAGE and either stained with PAGE Blue and Periodic Acid Schiff (PAS) in order to visualize total and glycosylated proteins respectively, or blotted onto nitrocellulose membranes for VOPBA. We included porcine mucins as a control of highly (O-)glycosylated proteins. At the first glance, VOPBA revealed that JcDV binds to most if not all the PM proteins labelled by PAGE blue and PAS combined, although with different intensities (Figure 5B). Interestingly, no binding was observed with the porcine mucins which might support some specificity for insect glycans. More specifically, a set of JcDV-interacting proteins was identified at high molecular weights (>250 kDa) including proteins with a pattern similar to porcine mucins. These proteins were labelled with PAGE blue and PAS or only with PAS suggesting that they are mostly and probably highly glycosylated (Figure 5B). Interestingly, proteins at 180 kDa displayed a higher intensity as they are in a relative lower amount (according to PAGE blue), which suggests higher affinity for JcDV. Proteins interacting with JcDV were detected at 150–200 kDa and 25–60 kDa, and corresponding to proteins with low or no glycosylation (according to PAS staining).

In total, 10 bands representing JcDV interacting proteins are reproducibly obtained with VOPBA. Noteworthy, each band probably include several proteins and/or isoform/glycoform of the same proteins.

**Figure 6:**
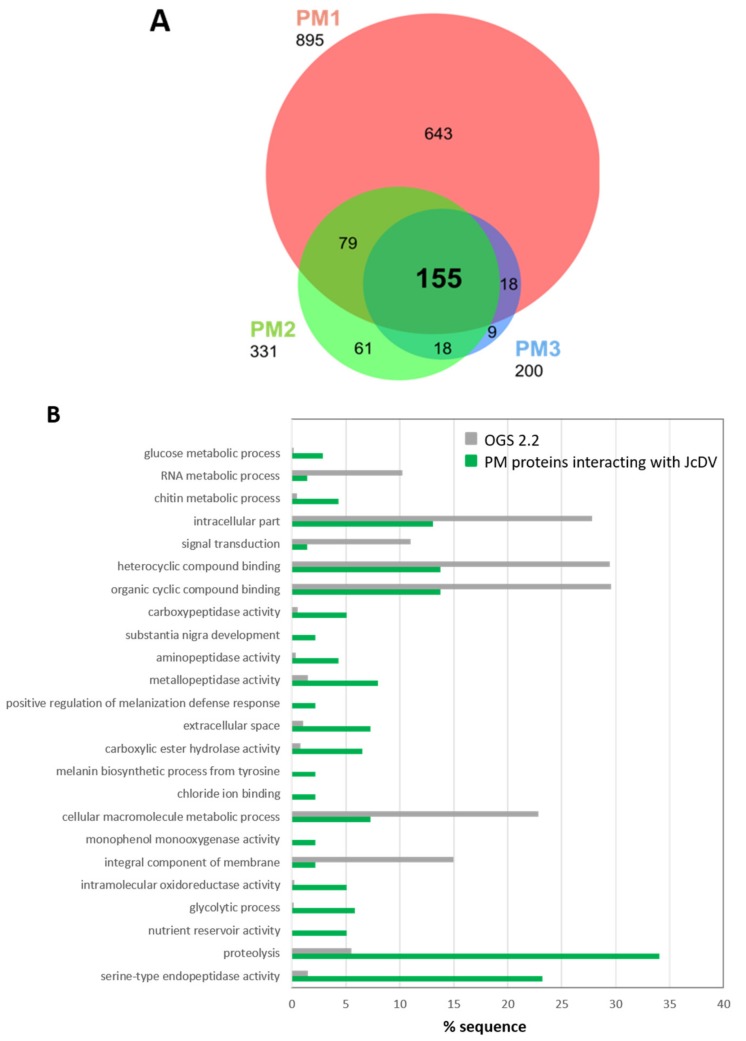
(**A**) Venn diagram of three replicates of proteomic analysis (PM1, PM2, PM3) of PM proteins interacting with JcDV. Protein bands revealed by VOPBA were cut in SDS-PAGE gel stained with Page blue and analyzed by LC–MS/MS as described in Methods. Among the 155 proteins common to the three replicates, 138 were annotated in the reference genome of *S. frugiperda* [33]. (**B**) GO terms enrichment for the 138 common annotated PM proteins interacting with JcDV (in green), compared to the reference in grey (predicted proteins from OGS2.2 *S. frugiperda* genome) (FDR set at 0.01). Specific enrichment in JcDV interacting proteins is considered when the green bars exceed the greys (controls). The 138 common PM proteins were assignated to the GO terms using Blast2Go software.

Proteins corresponding to these 10 bands were next analyzed by LC–MS/MS mass spectrometry. We only considered 155 proteins that were shared between 3 replicates (Figure 6A), out of which 138 were annotated in the reference genome of *S. frugiperda* [33]. These proteins are PM structural proteins (i.e., peritrophins including intestinal mucins) and PM-associated proteins (enzymes, i.e., serine proteases and aminopeptidases N (APN) (Supplementary Materials Table S1). Gene ontology (GO) annotation confirmed the enrichment in proteolytic activities (particularly serine-type endopeptidases) and chitin synthesis, which are consistent with the PM composition and the gut function (Figure 6B). Interestingly among the set of proteins >150 kDa, we identified intestinal mucins, an ATP binding cassette A type 5 (ABCA5) transporter and aminopeptidases N (Supplementary Materials Table S1); the latter being known receptors for a number of viruses and for the Cry toxins from *Bacillus thuringiensis* [49,50,51,52].

These results show that JcDV capsids can recognize and bind to the different components of the PM including chitin and several highly glycosylated proteins, both structural components of the PM (mucin, peritrophins) or associated proteins (enzymes).

### 3.4. JcDV Early Pathogenesis Induces Changes in Midgut Metabolism

**Figure 7:**
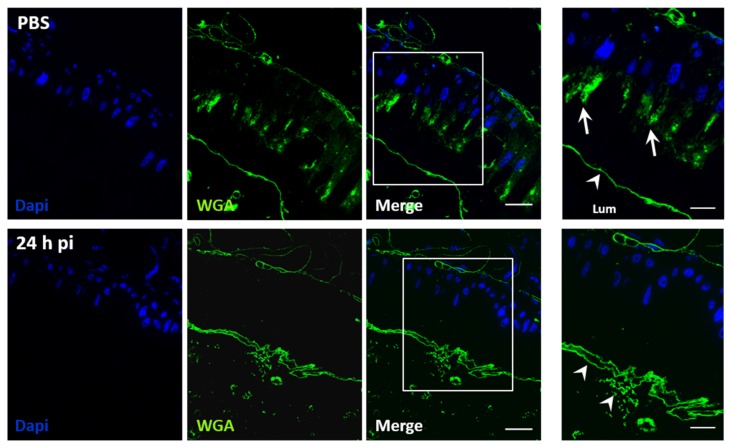
JcDV oral infection induces PM disorganization. Caterpillars (L3) were orally infected with JcDV (10^9^ veg/caterpillar) or fed with PBS as a control. At 24 h p.i., semi-thin transversal sections (1 μm) were prepared and processed for immunolabeling as described in Methods. WGA-FITC was used to visualize chitin synthesis and PM structure (in green). Nuclei are in blue (Dapi). Arrows show GlcNAc secretion by microvilli and arrowhead the PM. Lum, midgut lumen. Bars, 20 μm.

JcDV recognition and binding to glycans of the PM concentrates viral particles close to the epithelial surface, which raised questions about the mechanism involved to cross over and reach the midgut receptor(s). We hypothesized that capsids aggregation on the matrix can result in its disorganization, in a way similar to chitin-binding wheat germ agglutinin (WGA) lectin or Calcofluor [53]. To test this hypothesis, we used fluorescent WGA-labelling (WGA-FITC) to label chitin and thus examine chitin fibrils formation and PM organization. Third-instar caterpillars were fed with JcDV and then sacrificed at 1 day p.i. to dissect and prepare midguts for semi-thin sections and WGA labelling. As shown in Figure 7, the labelling of the PM (green) lined the apical surface of the epithelium in non-infected larvae (PBS condition, Figure 7, upper panel). In addition, we observed a specific labelling at the apex of columnar cells, probably corresponding to microvillar secretion of GlcNAc from these cells. By contrast, the labelling lining the epithelium appeared discontinuous following infection (Figure 7, lower panel), displaying a disorganized pattern reminiscent of the PM structure observed for caterpillars fed with the WGA lectin [53]. Moreover, intracellular labelling was no longer observed in the sections from infected caterpillars suggesting an arrest of GlcNAc secretion from the cells following early infection, i.e., before we can detect virus replication in subepithelial tissues [13]. These results thus support the hypothesis that JcDV binding on the PM and transcytosis is associated with a loss in its integrity, which might reveal gut dysfunction.

**Figure 8:**
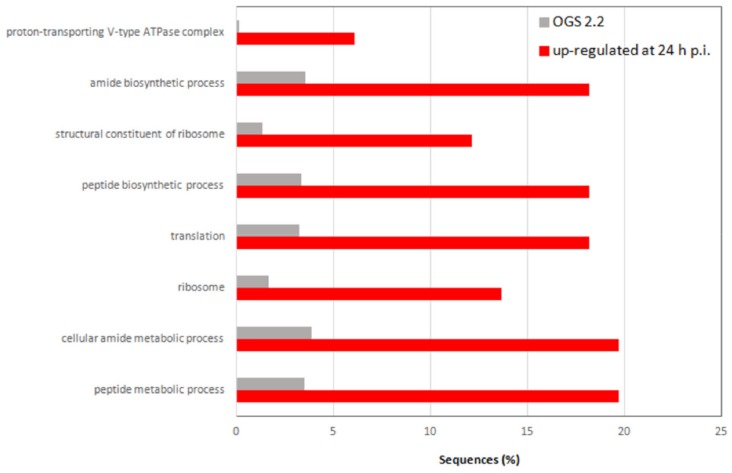
GO terms enrichment between infected and non-infected midgut DGE libraries, for the up-regulated genes at 24 h p.i. (in red), compared to the *S. frugiperda* reference genome (in grey) (OGS2.2 version; [33]) (FDR set at 0.01). The Blast2Go software was used for the assignation to the GO terms.

To determine the midgut response following JcDV break in, we analyzed the transcriptomic response. We used Digital Gene Expression (DGE) based on the Serial Analysis of Gene Expression approach [35]. This method involves the sequencing and quantification of end tagged short cDNA fragments (i.e., Tags), which enables quantitative differential gene-expression analysis. We built four cDNA libraries from midguts of mock-and infected larvae (Supplementary Materials Tables S2 and S3). Tag sequences were mapped to the genome and transcriptome of *S. frugiperda* ([33,38]) and to the JcDV genome. None of the tags were assigned to viral transcripts, which is consistent with JcDV pathogenesis excluding replication in midgut cells [13]. Pie charts represented GO assignment corresponding to unique transcripts at 1 and 3 days p.i. that displayed a differential expression at least 5-fold up- or down-regulated (Supplementary Materials Figure S4A,B). Interestingly, the distribution of GO terms was roughly similar at 1 and 3 days p.i., suggesting that the overall intestinal response to JcDV oral infection was poorly affected by virus replication going on in subepithelial tissues (Supplementary Materials Figure S4A,B). We observed only GO terms enrichment for the over-represented transcripts at 1-day p.i., more specifically in functions involved in metabolic processes including translation (i.e., regulation of biological processes, response to stimuli and signaling) at 1 day p.i., that might indicate that JcDV intrusion induces a rapid metabolic response in the gut (Figure 8). Interestingly, these changes did not change significantly at 3 days p.i. suggesting that the gut response is rapidly initiated by JcDV transcytosis and was not affected by the viral replication that takes place in underlying tissues. We did not observe any significant activation of genes involved in “inflammation” nor in the canonical gut immune response. However, we observed an increased expression in cytochrome P450 and catalase genes that might indicate a response of the cells to the ongoing infection.

Concerning the expression of genes involved in carbohydrate, chitin metabolism and/or coding for mucin-like proteins (all the corresponding results are provided in Supplementary Materials Table S4), we only observed few changes including a trehalose transporter and an intestinal mucin, both down-regulated from day 1 p.i., and a chitinase up-regulated at day 3 p.i.. Except for an aminopeptidase N, no gene corresponding to the JcDV interacting proteins identified by VOPBA displayed a transcriptional change.

We did not observe any significant activation of genes involved in “inflammation” nor in the canonical gut immune response. However, we observed an increased expression in cytochrome P450 and catalase genes that might indicate a response of the cells to the ongoing infection.

Altogether these results show that JcDV infection induces rapid changes in the gut, particularly in translation and metabolism, within 24 h p.i.. Both increased molecular activities might favor viral invasion by supporting the increased energetic demand associated with virus replication in target tissues. Interestingly, the canonical midgut immune system did not detect JcDV break in and transport across the epithelium.

## 4. Discussion

How densoviruses cope with the forest of glycans that constitute extracellular matrices and decorates insect cell surfaces has been so far a neglected step of their early pathogenesis. Results presented here show that JcDV capsids display carbohydrate-binding properties that insure recognition of the peritrophic matrix and determines caterpillars oral infection. We found that capsids can bind to the different components of the PM and their agglutination on the PM surface is associated with the disruption of its organization. Furthermore, we showed that this primary step of infection of caterpillars results in a series of physiological changes in the midgut including an arrest of chitin synthesis by epithelial cells.

### 4.1. JcDV Recognition and Binding to Glycans

The PM is an obligatory binding platform for capsids to avoid elimination and get closer to the epithelial cell surface where receptor recognition can occur. However, strong attachment to glycans composing the PM would trap capsids there and thus impair their physical connection with the receptor(s). Therefore, a first hypothesis is that the “stickiness” of the capsids is balanced to bind and unbind glycans. We used competitions assays with monosaccharides to test this “bind and release” hypothesis and our results showed that indeed, capsids have an affinity for glycans, although the concentration range of the monosaccharides (mM) we tested likely indicates their poor affinity for the capsids. As these monosaccharides could compete capsids away from the PM further suggests that glycan-capsids interaction are probably of low affinity.

The issue for bound viral particles is then to move across the PM. Our experiments show that capsid binding results in a structural disorganization of the PM similar to effects induced by chitin-binding lectin WGA and Calcofluor. Such capsid-induced disruption of the PM thus favors a second hypothesis, involving a “saturate and pass through” mechanism, where bound capsids are not released but open a way for viral particles to cross over. Such “cooperative” mechanism of the capsids to overcome the PM is supported by the fact that PM disruption is enhanced by virus concentration and decreased as caterpillars age. Such developmental resistance of *S. frugiperda* has been also reported following baculovirus infections and a high synergism with Calcofluor was obtained at late instars (e.g., > 60-fold at 4th instar vs. 3 to 6-fold at 2nd and 3rd instars) [54].The structure and the composition of the PM can vary as caterpillars grow and feed, or between populations, which might impact virus-PM interactions and consequently insect susceptibility [16,17,55]. Whether PM disruption results from mechanical stresses on chitin fibers similar to calcofluor and probably WGA lectins or from an enzymatic activity of the capsids (i.e., of VP4) remains to be analyzed more thoroughly. Understanding the early interaction of JcDV with the glycans of the PM within species, i.e., along the larval development is of importance to develop biocontrol strategies against insect pests.

### 4.2. The Role of PM Glycans in the Species Barrier to Densovirus Infection

Whether or not the PM could contribute to the species barrier against densovirus infection is unknown. A better understanding of the structure and the glycan composition of the PM in *S. frugiperda* together with comparative studies in different lepidopteran species are essential to go further on the role of the PM in densovirus infection.

Structure-fonction studies of the capsid of parvoviruses infecting vertebrates, in particular for species in the genera *Protoparvovirus* and *Dependovirus*, have highlighted the importance of glycans recognition on tissue tropism, pathogenicity, and host range adaption [6,45,56,57,58,59]. Regarding densoviruses, information and capsid structure-function studies is poor [60]. We performed preliminary assays with a glycan array from the Consortium for functional glycomics (http://www.functionalglycomics.org/fg/, AS Gosselin-Grenet, unpublished data). Although this array represents mammalian glycans, the specific recognition of JcDV capsids by paucimannose, which are particularly abundant in insects, suggests some specificity in the interaction of the capsid with glycans. An “insect array” would be of interest to explore glycan ligands affinity and specificity for densovirus capsids. Morevover, insects are particularly “handly” animal models for structure-function assays in vivo.

### 4.3. JcDV Early Infection Induces Physiological Changes in the Midgut

We showed that JcDV early infection triggers an arrest of GlcNAc secretion, which might considerably weaken the PM and explain the disorganized pattern observed with chitin-labelling at 1 day p.i. Interestingly, these changes are observed before we could detect JcDV transcription/replication in primary targeted cells [13], suggesting that these effects are induced as a consequence of the transport of the viral particles across the epithelium. It has been reported that specific drugs that disorganize microtubules induce an arrest of chitin synthesis [41,61]. We speculate that JcDV transcytosis might induce some stress on the cytoskeletal network, similar to microtubules disorganizing drugs. It is worthy to note that chitin synthesis arrest was not associated with a drop in the expression of chitin synthase genes. However, we cannot exclude to have missed enzymes due to some lack in the annotation of the genome of *S. frugiperda* [33].

Last, our results showed that JcDV capsids can also interact with components of the luminal compartment including food and bacteria. The competition of the capsid with monosaccharides for binding the PM, suggests that food components could interfere with infection. Indeed, plants contain compounds that can interfere with PM synthesis, which has been shown to consequently affect baculovirus infection [55] (Chen et al., 2018). Regarding bacteria, it has been shown recently that the PM controls commensal bacteria, and conversely that its synthesis and integrity can be microbiota-dependent, i.e., the gut microbiota inducing the expression of components of the peritrophic matrix [62,63]. So, it is plausible that food and microbiota can modify the outcome of the densovirus infection, either by directly competing for binding the PM, or indirectly by modulating the composition of the PM.

The binding of densovirus capsids to a wide array of glycans questioned about the role of this “stickiness” in the whole infection cycle including transmission. Groundbreaking articles have shown the role of microbiota polysaccharides including GlcNAc, on the infectivity and thermostability of picornaviruses [64,65,66], whose capsid share structural similarity with parvoviruses [25]. It is tempting to speculate that densovirus “stickiness” can similarly impact their transmission, which might participate to their success if only among arthropods that occupy extremely diversified ecosystems [67]. Such consideration could also apply to parvoviruses going through faecal–oral route and environmental contamination [58,68]. More generally, stickiness is a major issue for most viruses to and mathematical models have been applied to influenza. They predict that a maximum stickiness favors a maximum fitness [69]. However, trade-off probably exists in biological systems with an optimal “stickiness” that must be found to infect and leave a host for transmission.

Densoviruses can be highly pathogenic for insect pests and vectors, which have long stimulated their interest as biocontrol agents or genes vectors [70]. They are considered today with a renewed interest as solutions to control harmful insects are lacking, which encourages efforts to understand their pathogenesis and their specificity. Altogether our results suggest that PM glycans are crucial interacting components of the early JcDV pathogenesis. Exploring their diversity and their complexity in insects can also provide important cues on the extend of the mechanisms that determine densovirus specificity.

## Supplementary Materials

The following are available online at https://www.mdpi.com/1999-4915/11/9/870/s1, Figure S1. Calcofluor increases *S. frugiperda* caterpillars susceptibility to densovirus oral infection. Figure S2. Virus interaction with monosaccharides did not interfere with anti-capsid antibody recognition. Figure S3. Replicates of survival curves of caterpillars infected by JcDV. Figure S4. JcDV oral infection induces midgut gene expression modulation. Table S1. Annotation of PM proteins interacting with JcDV in VOPBA. Table S2. Characteristics of DGE libraries generated from caterpillars orally infected with JcDV. Table S3. Characteristics of annotated Tags and transcripts. Table S4. Annotation of regulated intestinal transcripts following oral JcDV infection.
