# Supplementary material for: Interaction of a Densovirus with Glycans of the Peritrophic Matrix Mediates Oral Infection of the Lepidopteran Pest Spodoptera frugiperda
Source: Viruses. 2019 Sep 17;11(9):870. doi: 10.3390/v11090870 (PMC6783882; doi:10.3390/v11090870)
Supplement: Supplementary file 1 [file viruses-11-00870-s001.zip › Supplementary figures and Tables/Supp Fig1.pdf]

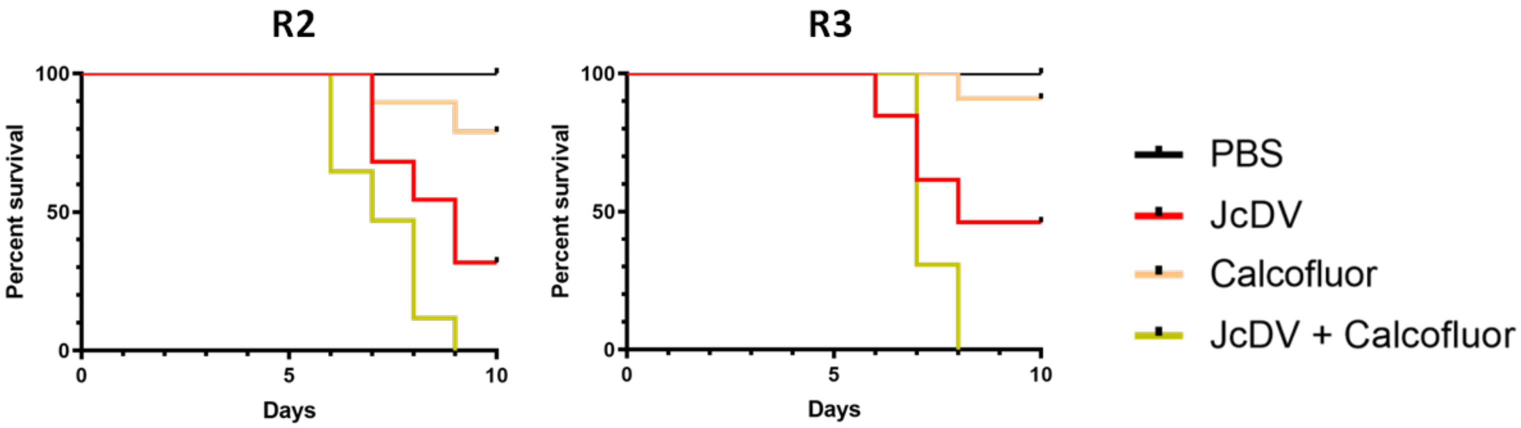

	Pairwise comparison	Log-rank (Mantel-Cox)	Gehan-Breslow-Wilcoxon
R2	JcDV + Calcofluor *vs* JcDV	*P* value = 0.0013 **	*P* value = 0,0026 **
	Calcofluor *vs* JcDV	*P* value = 0.0027 **	*P* value = 0.0039 **
R3	JcDV + Calcofluor *vs* JcDV	*P* value = 0.0352 *	*P* value = 0.1110 ns
	Calcofluor *vs* JcDV	*P* value = 0.0046 **	*P* value = 0.0057 **
