# Supplementary material for: Interaction of a Densovirus with Glycans of the Peritrophic Matrix Mediates Oral Infection of the Lepidopteran Pest Spodoptera frugiperda
Source: Viruses. 2019 Sep 17;11(9):870. doi: 10.3390/v11090870 (PMC6783882; doi:10.3390/v11090870)
Supplement: Supplementary file 1 [file viruses-11-00870-s001.zip › Supplementary figures and Tables/Supp Fig3_AB.pdf]

A

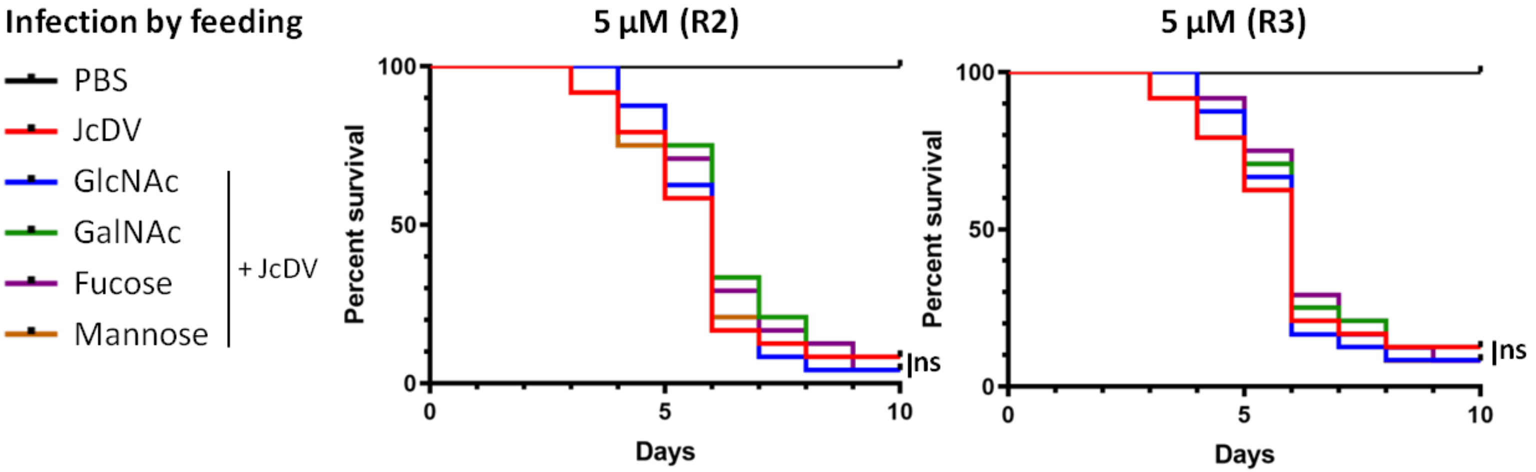

B

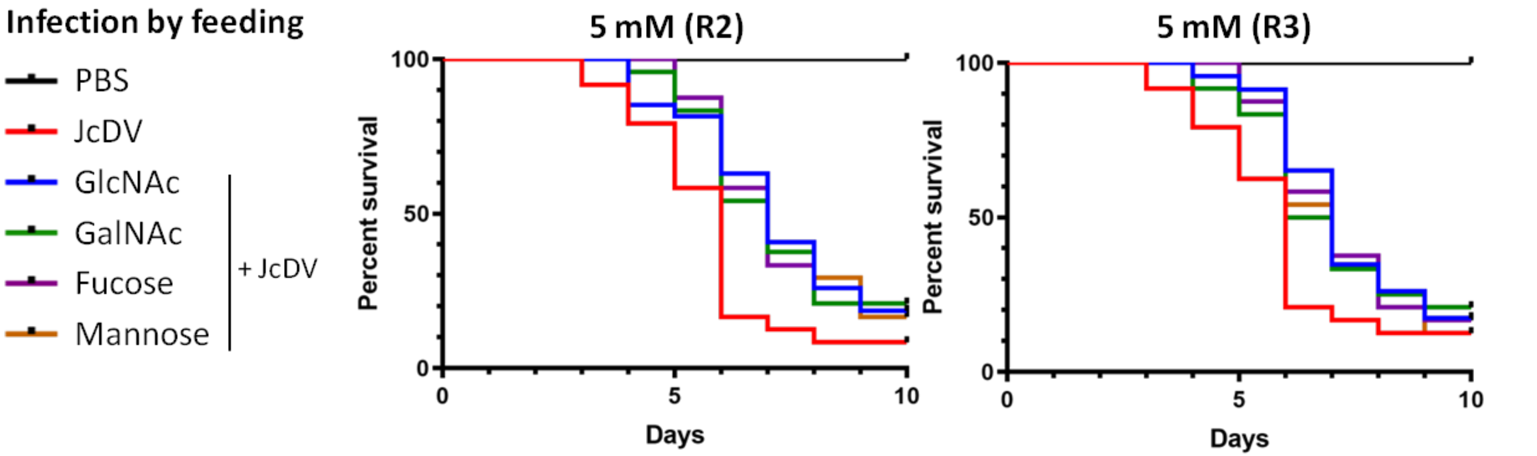

	Pairwise comparison	Log-rank (Mantel-Cox)	Gehan-Breslow-Wilcoxon
R2	JcDV + GlcNAc *vs* JcDV	*P* value = 0.0143 *	*P* value = 0,006 **
	JcDV + GalNAc *vs* JcDV	*P* value = 0.0174 *	*P* value = 0.007 **
	JcDV + Fucose *vs* JcDV	*P* value = 0.009 **	*P* value = 0.0023 **
	JcDV + Mannose *vs* JcDV	*P* value = 0.0157 *	*P* value = 0.0038 **
R3	JcDV + GlcNAc *vs* JcDV	*P* value = 0.0371 *	*P* value = 0,0047 **
	JcDV + GalNAc *vs* JcDV	*P* value = 0.0954 ns	*P* value = 0.0371 *
	JcDV + Fucose *vs* JcDV	*P* value = 0.0588 ns	*P* value = 0.0099 **
	JcDV + Mannose *vs* JcDV	*P* value = 0.1179 ns	*P* value = 0.0211 *
