# Supplementary material for: Interaction of a Densovirus with Glycans of the Peritrophic Matrix Mediates Oral Infection of the Lepidopteran Pest Spodoptera frugiperda
Source: Viruses. 2019 Sep 17;11(9):870. doi: 10.3390/v11090870 (PMC6783882; doi:10.3390/v11090870)
Supplement: Supplementary file 1 [file viruses-11-00870-s001.zip › Supplementary figures and Tables/Supp Fig4_A.pdf]

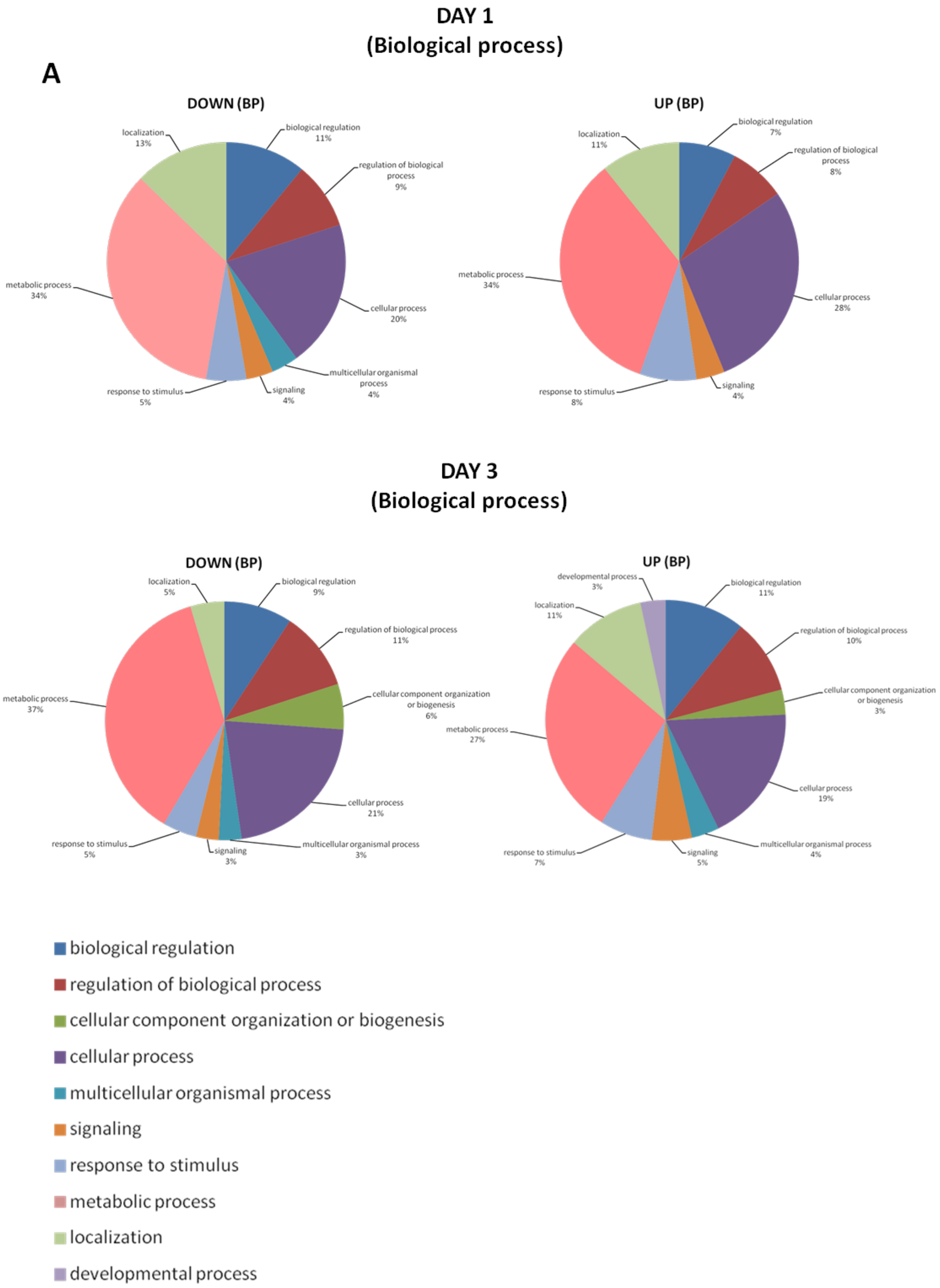
DAY 1
(Biological process)
A
DOWN (BP)
localization 13%
biological regulation 11%
regulation of biological process 9%
cellular process 20%
multicellular organismal process 4%
signaling 4%
response to stimulus 5%
metabolic process 34%
UP (BP)
localization 11%
biological regulation 7%
regulation of biological process 8%
cellular process 28%
signaling 4%
response to stimulus 8%
metabolic process 34%
DAY 3
(Biological process)
DOWN (BP)
localization 5%
biological regulation 9%
regulation of biological process 11%
cellular component organization or biogenesis 6%
cellular process 21%
multicellular organismal process 3%
signaling 3%
response to stimulus 5%
metabolic process 37%
UP (BP)
developmental process 3%
localization 11%
biological regulation 11%
regulation of biological process 10%
cellular component organization or biogenesis 3%
cellular process 19%
multicellular organismal process 4%
signaling 5%
response to stimulus 7%
metabolic process 27%
biological regulation
regulation of biological process
cellular component organization or biogenesis
cellular process
multicellular organismal process
signaling
response to stimulus
metabolic process
localization
developmental process
