# Supplementary material for: Interaction of a Densovirus with Glycans of the Peritrophic Matrix Mediates Oral Infection of the Lepidopteran Pest Spodoptera frugiperda
Source: Viruses. 2019 Sep 17;11(9):870. doi: 10.3390/v11090870 (PMC6783882; doi:10.3390/v11090870)
Supplement: Supplementary file 1 [file viruses-11-00870-s001.zip › Supplementary figures and Tables/Supp Fig4_B.pdf]

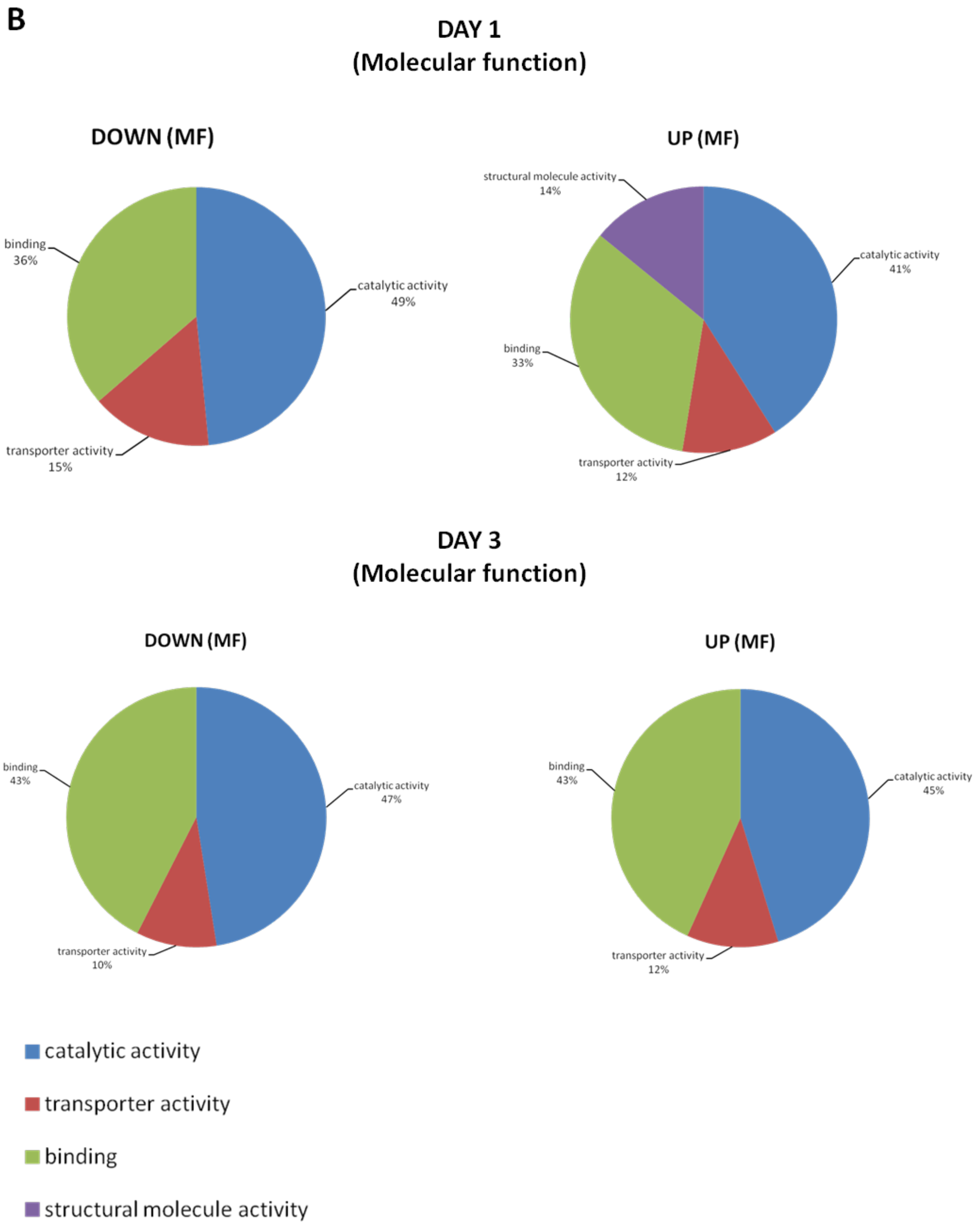
B
DAY 1
(Molecular function)
DOWN (MF)
binding
36%
catalytic activity
49%
transporter activity
15%
UP (MF)
structural molecule activity
14%
catalytic activity
41%
binding
33%
transporter activity
12%
DAY 3
(Molecular function)
DOWN (MF)
binding
43%
catalytic activity
47%
transporter activity
10%
UP (MF)
binding
43%
catalytic activity
45%
transporter activity
12%
catalytic activity
transporter activity
binding
structural molecule activity
