# Supplementary material for: Interaction of a Densovirus with Glycans of the Peritrophic Matrix Mediates Oral Infection of the Lepidopteran Pest Spodoptera frugiperda
Source: Viruses. 2019 Sep 17;11(9):870. doi: 10.3390/v11090870 (PMC6783882; doi:10.3390/v11090870)
Supplement: Supplementary file 1 [file viruses-11-00870-s001.zip › Supplementary figures and Tables/Supp Table1.pdf]

Name	Description	#GO	GO IDs	GO Names
GSSPFG00020857001.3-PA	14-3-3 protein epsilon isoform x1	41	P:GO:0000075; P:GO:0000086; P:GO:0001764; P:GO:0003064; F:GO:0003723; F:GO:0004497; C:GO:0005739; C:GO:0005829; C:GO:0005871; C:GO:0005925; P:GO:0006367; P:GO:0006605; P:GO:0007264; F:GO:0015459; P:GO:0016032; F:GO:0019904; P:GO:0021762; P:GO:0021766; P:GO:0021987; F:GO:0023026; C:GO:0030424; C:GO:0030659; F:GO:0031625; P:GO:0035329; C:GO:0042470; F:GO:0042826; P:GO:0043281; F:GO:0044325; F:GO:0046982; P:GO:0048011; F:GO:0050815; P:GO:0055114; P:GO:0060306; C:GO:0070062; P:GO:0086013; P:GO:0086091; P:GO:0097193; P:GO:1900034; P:GO:1900740; P:GO:1901016; P:GO:1902309	P:cell cycle checkpoint; P:G2/M transition of mitotic cell cycle; P:neuron migration; P:regulation of heart rate by hormone; F:RNA binding; F:monooxygenase activity; C:mitochondrion; C:cytosol; C:kinesin complex; C:focal adhesion; P:transcription initiation from RNA polymerase II promoter; P:protein targeting; P:small GTPase mediated signal transduction; F:potassium channel regulator activity; P:viral process; F:protein domain specific binding; P:substantia nigra development; P:hippocampus development; P:cerebral cortex development; F:MHC class II protein complex binding; C:axon; C:cytoplasmic vesicle membrane; F:ubiquitin protein ligase binding; P:hippo signaling; C:melanosome; F:histone deacetylase binding; P:regulation of cysteine-type endopeptidase activity involved in apoptotic process; F:ion channel binding; F:protein heterodimerization activity; P:neurotrophin TRK receptor signaling pathway; F:phosphoserine residue binding; P:oxidation-reduction process; P:regulation of membrane repolarization; C:extracellular exosome; P:membrane repolarization during cardiac muscle cell action potential; P:regulation of heart rate by cardiac conduction; P:intrinsic apoptotic signaling pathway; P:regulation of cellular response to heat; P:positive regulation of protein insertion into mitochondrial membrane involved in apoptotic signaling pathway; P:regulation of potassium ion transmembrane transporter activity; P:negative regulation of peptidyl-serine dephosphorylation
GSSPFG00022879001-PA	14-3-3 protein zeta isoform x1	2	C:GO:0005737; F:GO:0019904	C:cytoplasm; F:protein domain specific binding
GSSPFG00016664001.1-PA	af361483_1chymotrypsin inhibitor ci-8a	3	C:GO:0005615; P:GO:0010466; F:GO:0030414	C:extracellular space; P:negative regulation of peptidase activity; F:peptidase inhibitor activity
GSSPFG00012908001.3-PA	alkaline a-like	2	F:GO:0004252; P:GO:0006508	F:serine-type endopeptidase activity; P:proteolysis
GSSPFG00019582001.4-PA	alkaline nuclease	4	F:GO:0003676; P:GO:0008152; F:GO:0016787; F:GO:0046872	F:nucleic acid binding; P:metabolic process; F:hydrolase activity; F:metal ion binding
GSSPFG00004452001-PA	alpha amylase	3	P:GO:0005975; F:GO:0016787; F:GO:0043169	P:carbohydrate metabolic process; F:hydrolase activity; F:cation binding
GSSPFG00027172001.1-PA	alpha-amylase 2	8	F:GO:0004556; P:GO:0005975; P:GO:0006567; F:GO:0008890; P:GO:0009058; F:GO:0016874; F:GO:0030170; F:GO:0043169	F:alpha-amylase activity; P:carbohydrate metabolic process; P:threonine catabolic process; F:glycine C-acetyltransferase activity; P:biosynthetic process; F:ligase activity; F:pyridoxal phosphate binding; F:cation binding
GSSPFG00000424001-PA	alpha-amylase 4n-like	3	F:GO:0004556; P:GO:0005975; F:GO:0043169	F:alpha-amylase activity; P:carbohydrate metabolic process; F:cation binding
GSSPFG00025752001.1-PA	aminopeptidase n	5	F:GO:0004177; P:GO:0006508; F:GO:0008237; F:GO:0008270; C:GO:0016020	F:aminopeptidase activity; P:proteolysis; F:metallopeptidase activity; F:zinc ion binding; C:membrane
GSSPFG00004595001-PA	aminopeptidase n	2	F:GO:0046872; F:GO:0070011	F:metal ion binding; F:peptidase activity, acting on L-amino acid peptides
GSSPFG00026992001-PA	aminopeptidase n	5	F:GO:0004177; P:GO:0006508; F:GO:0008237; F:GO:0008270; C:GO:0016020	F:aminopeptidase activity; P:proteolysis; F:metallopeptidase activity; F:zinc ion binding; C:membrane
GSSPFG00019131001-PA	aminopeptidase n	5	F:GO:0004177; P:GO:0006508; F:GO:0008237; F:GO:0008270; C:GO:0016020	F:aminopeptidase activity; P:proteolysis; F:metallopeptidase activity; F:zinc ion binding; C:membrane
GSSPFG00002605001-PA	aminopeptidase n1	4	F:GO:0004177; P:GO:0006508; F:GO:0008237; F:GO:0008270	F:aminopeptidase activity; P:proteolysis; F:metallopeptidase activity; F:zinc ion binding
GSSPFG00002604001-PA	aminopeptidase n4	4	F:GO:0004177; P:GO:0006508; F:GO:0008237; F:GO:0008270	F:aminopeptidase activity; P:proteolysis; F:metallopeptidase activity; F:zinc ion binding
GSSPFG00006479001.1-PA	aminopeptidase n5	4	F:GO:0004177; P:GO:0006508; F:GO:0008237; F:GO:0008270	F:aminopeptidase activity; P:proteolysis; F:metallopeptidase activity; F:zinc ion binding
GSSPFG00027324001-PA	anhydro-d-fructose reductase-like isoform x3	2	F:GO:0016491; P:GO:0055114	F:oxidoreductase activity; P:oxidation-reduction process
GSSPFG00022331001.1-PA	apolipophorins	7	F:GO:0005488; P:GO:0006810; P:GO:0009987; P:GO:0033036; C:GO:0044464; P:GO:0051179; P:GO:0065007	F:binding; P:transport; P:cellular process; P:macromolecule localization; C:cell part; P:localization; P:biological regulation
GSSPFG00013898001-PA	apolipophorins	7	F:GO:0005488; P:GO:0006810; P:GO:0009987; P:GO:0033036; C:GO:0044464; P:GO:0051179; P:GO:0065007	F:binding; P:transport; P:cellular process; P:macromolecule localization; C:cell part; P:localization; P:biological regulation
GSSPFG00000973001-PA	apolipoprotein d-like protein	2	C:GO:0005576; F:GO:0031409	C:extracellular region; F:pigment binding
GSSPFG00020046001-PA	arginine kinase	3	F:GO:0004054; F:GO:0005524; P:GO:0016310	F:arginine kinase activity; F:ATP binding; P:phosphorylation
GSSPFG00017029001-PA	arylphorin precursor	2	C:GO:0005615; F:GO:0045735	C:extracellular space; F:nutrient reservoir activity
GSSPFG00017028001-PA	arylphorin precursor	2	C:GO:0005615; F:GO:0045735	C:extracellular space; F:nutrient reservoir activity
GSSPFG00017030001-PA	arylphorin subunit alpha	2	C:GO:0005615; F:GO:0045735	C:extracellular space; F:nutrient reservoir activity
GSSPFG00017555001.4-PA	atp-binding cassette sub-family a member 5-like isoform x1	6	F:GO:0005215; F:GO:0005524; P:GO:0006810; P:GO:0008152; C:GO:0016021; F:GO:0016887	F:transporter activity; F:ATP binding; P:transport; P:metabolic process; C:integral component of membrane; F:ATPase activity
GSSPFG00031214001-PA	beta- partial	24	C:GO:0000790; F:GO:0000978; F:GO:0000980; P:GO:0001895; F:GO:0005524; C:GO:0005829; C:GO:0005886; C:GO:0005925; F:GO:0019894; P:GO:0021762; C:GO:0030863; F:GO:0030957; F:GO:0031492; C:GO:0035267; C:GO:0036464; F:GO:0042802; P:GO:0043044; C:GO:0043209; F:GO:0050998; C:GO:0070062; P:GO:0070527; C:GO:0070688; C:GO:0072562; C:GO:0097433	C:nuclear chromatin; F:RNA polymerase II proximal promoter sequence-specific DNA binding; F:RNA polymerase II distal enhancer sequence-specific DNA binding; P:retina homeostasis; F:ATP binding; C:cytosol; C:plasma membrane; C:focal adhesion; F:kinesin binding; P:substantia nigra development; C:cortical cytoskeleton; F:Tat protein binding; F:nucleosomal DNA binding; C:NuA4 histone acetyltransferase complex; C:cytoplasmic ribonucleoprotein granule; F:identical protein binding; P:ATP-dependent chromatin remodeling; C:myelin sheath; F:nitric-oxide synthase binding; C:extracellular exosome; P:platelet aggregation; C:obsolete MLL5-L complex; C:blood microparticle; C:dense body

GSSPFG00026444001-PA	beta- partial	24	C:GO:0000790; F:GO:0000978; F:GO:0000980; P:GO:0001895; F:GO:0005524; C:GO:0005829; C:GO:0005886; C:GO:0005925; F:GO:0019894; P:GO:0021762; C:GO:0030863; F:GO:0030957; F:GO:0031492; C:GO:0035267; C:GO:0036464; F:GO:0042802; P:GO:0043044; C:GO:0043209; F:GO:0050998; C:GO:0070062; P:GO:0070527; C:GO:0070688; C:GO:0072562; C:GO:0097433	C:nuclear chromatin; F:RNA polymerase II proximal promoter sequence-specific DNA binding; F:RNA polymerase II distal enhancer sequence-specific DNA binding; P:retina homeostasis; F:ATP binding; C:cytosol; C:plasma membrane; C:focal adhesion; F:kinesin binding; P:substantia nigra development; C:cortical cytoskeleton; F:Tat protein binding; F:nucleosomal DNA binding; C:NuA4 histone acetyltransferase complex; C:cytoplasmic ribonucleoprotein granule; F:identical protein binding; P:ATP-dependent chromatin remodeling; C:myelin sheath; F:nitric-oxide synthase binding; C:extracellular exosome; P:platelet aggregation; C:obsolete MLL5-L complex; C:blood microparticle; C:dense body
GSSPFG00017294001-PA	beta-glucanase	2	F:GO:0004553; P:GO:0005975	F:hydrolase activity, hydrolyzing O-glycosyl compounds; P:carbohydrate metabolic process
GSSPFG00002042001.2-PA	beta-glucanase	2	F:GO:0004553; P:GO:0005975	F:hydrolase activity, hydrolyzing O-glycosyl compounds; P:carbohydrate metabolic process
GSSPFG00017301001.1-PA	carboxypeptidase b precursor	3	F:GO:0004181; P:GO:0006508; F:GO:0008270	F:metallocarboxypeptidase activity; P:proteolysis; F:zinc ion binding
GSSPFG00017300001.1-PA	carboxypeptidase b precursor	3	F:GO:0004181; P:GO:0006508; F:GO:0008270	F:metallocarboxypeptidase activity; P:proteolysis; F:zinc ion binding
GSSPFG00030472001-PA	carboxypeptidase b-like	6	F:GO:0004181; F:GO:0004930; P:GO:0006508; P:GO:0007186; F:GO:0008270; C:GO:0016021	F:metallocarboxypeptidase activity; F:G protein-coupled receptor activity; P:proteolysis; P:G protein-coupled receptor signaling pathway; F:zinc ion binding; C:integral component of membrane
GSSPFG00030477001.4-PA	catalase	29	F:GO:0000981; F:GO:0001158; P:GO:0003007; F:GO:0004096; F:GO:0004102; C:GO:0005634; C:GO:0005777; C:GO:0005891; P:GO:0007274; P:GO:0007517; P:GO:0008292; F:GO:0008332; P:GO:0008340; P:GO:0016339; F:GO:0020037; P:GO:0035206; P:GO:0038001; P:GO:0042542; P:GO:0042673; P:GO:0042682; P:GO:0042744; P:GO:0045471; P:GO:0045676; P:GO:0045944; F:GO:0046872; P:GO:0048813; P:GO:0055114; P:GO:0070509; P:GO:0070588	F:DNA-binding transcription factor activity, RNA polymerase II-specific; F:enhancer sequence-specific DNA binding; P:heart morphogenesis; F:catalase activity; F:choline O-acetyltransferase activity; C:nucleus; C:peroxisome; C:voltage-gated calcium channel complex; P:neuromuscular synaptic transmission; P:muscle organ development; P:acetylcholine biosynthetic process; F:low voltage-gated calcium channel activity; P:determination of adult lifespan; P:calcium-dependent cell-cell adhesion via plasma membrane cell adhesion molecules; F:heme binding; P:regulation of hemocyte proliferation; P:paracrine signaling; P:response to hydrogen peroxide; P:regulation of retinal cone cell fate specification; P:regulation of compound eye cone cell fate specification; P:hydrogen peroxide catabolic process; P:response to ethanol; P:regulation of R7 cell differentiation; P:positive regulation of transcription by RNA polymerase II; F:metal ion binding; P:dendrite morphogenesis; P:oxidation-reduction process; P:calcium ion import; P:calcium ion transmembrane transport
GSSPFG00019138001.5-PA	chymotrypsin	2	F:GO:0004252; P:GO:0006508	F:serine-type endopeptidase activity; P:proteolysis
GSSPFG00019137001.5-PA	chymotrypsin	2	F:GO:0004252; P:GO:0006508	F:serine-type endopeptidase activity; P:proteolysis
GSSPFG00012295001.5-PA	chymotrypsin	2	F:GO:0004252; P:GO:0006508	F:serine-type endopeptidase activity; P:proteolysis
GSSPFG00034352001.3-PA	chymotrypsin-like protease	2	F:GO:0004252; P:GO:0006508	F:serine-type endopeptidase activity; P:proteolysis
GSSPFG00034354001.3-PA	chymotrypsin-like protease	2	F:GO:0004252; P:GO:0006508	F:serine-type endopeptidase activity; P:proteolysis
GSSPFG00009808001.4-PA	chymotrypsin-like protease	2	F:GO:0004252; P:GO:0006508	F:serine-type endopeptidase activity; P:proteolysis
GSSPFG00008227001.3-PA	chymotrypsin-like protease	2	F:GO:0004252; P:GO:0006508	F:serine-type endopeptidase activity; P:proteolysis
GSSPFG00022970001.3-PA	chymotrypsin-like protease	2	F:GO:0004252; P:GO:0006508	F:serine-type endopeptidase activity; P:proteolysis
GSSPFG00030121001.3-PA	chymotrypsin-like protease	2	F:GO:0004252; P:GO:0006508	F:serine-type endopeptidase activity; P:proteolysis
GSSPFG00025114001.3-PA	chymotrypsin-like protease c9	3	F:GO:0004252; F:GO:0005515; P:GO:0006508	F:serine-type endopeptidase activity; F:protein binding; P:proteolysis
GSSPFG00032889001.4-PA	chymotrypsin-like proteinase	2	F:GO:0004252; P:GO:0006508	F:serine-type endopeptidase activity; P:proteolysis
GSSPFG00034358001.3-PA	chymotrypsin-like serine protease	2	F:GO:0004252; P:GO:0006508	F:serine-type endopeptidase activity; P:proteolysis
GSSPFG00034359001.3-PA	chymotrypsin-like serine protease	2	F:GO:0004252; P:GO:0006508	F:serine-type endopeptidase activity; P:proteolysis
GSSPFG00002322001.4-PA	chymotrypsin-like serine protease	1	F:GO:0008236	F:serine-type peptidase activity
GSSPFG00005604001.2-PA	chymotrypsin-like serine protease 14	3	F:GO:0008236; P:GO:0044238; P:GO:0071704	F:serine-type peptidase activity; P:primary metabolic process; P:organic substance metabolic process
GSSPFG00013449001.3-PA	chymotrypsin-like serine protease precursor	2	F:GO:0004252; P:GO:0006508	F:serine-type endopeptidase activity; P:proteolysis
GSSPFG00034231001-PA	cytosolic non-specific dipeptidase	5	F:GO:0004180; P:GO:0006508; F:GO:0008237; F:GO:0016805; F:GO:0034701	F:carboxypeptidase activity; P:proteolysis; F:metallopeptidase activity; F:dipeptidase activity; F:tripeptidase activity
GSSPFG00014405001-PA	dipeptidyl peptidase 4	3	P:GO:0006508; F:GO:0008236; C:GO:0016020	P:proteolysis; F:serine-type peptidase activity; C:membrane
GSSPFG00002766001-PA	elongation factor 1 alpha	9	F:GO:0003746; F:GO:0003924; F:GO:0005525; C:GO:0005811; C:GO:0005829; C:GO:0005853; C:GO:0005875; P:GO:0006414; P:GO:0008340	F:translation elongation factor activity; F:GTPase activity; F:GTP binding; C:lipid droplet; C:cytosol; C:eukaryotic translation elongation factor 1 complex; C:microtubule associated complex; P:translational elongation; P:determination of adult lifespan
GSSPFG00027742001-PA	elongation factor 1 alpha	9	F:GO:0003746; F:GO:0003924; F:GO:0005525; C:GO:0005811; C:GO:0005829; C:GO:0005853; C:GO:0005875; P:GO:0006414; P:GO:0008340	F:translation elongation factor activity; F:GTPase activity; F:GTP binding; C:lipid droplet; C:cytosol; C:eukaryotic translation elongation factor 1 complex; C:microtubule associated complex; P:translational elongation; P:determination of adult lifespan
GSSPFG00029239001-PA	ester hydrolase c11orf54 homolog	1	C:GO:0005634	C:nucleus
GSSPFG00003392001-PA	farnesoic acid o-methyl transferase	2	F:GO:0008168; P:GO:0032259	F:methyltransferase activity; P:methylation
GSSPFG00011246001-PA	farnesoic acid o-methyl transferase	2	F:GO:0008168; P:GO:0032259	F:methyltransferase activity; P:methylation
GSSPFG00008415001-PA	fructose -bisphosphate aldolase	2	F:GO:0004332; P:GO:0006096	F:fructose-bisphosphate aldolase activity; P:glycolytic process
GSSPFG00032332001.1-PA	fructose-bisphosphate aldolase isoform x1	2	F:GO:0004332; P:GO:0006096	F:fructose-bisphosphate aldolase activity; P:glycolytic process
GSSPFG00021650001-PA	glucose dehydrogenase	1	F:GO:0016491	F:oxidoreductase activity

GSSPFG00026492001.1-PA	glucosylceramidase-like	3	P:GO:0006665; F:GO:0016787; P:GO:0050794	P:sphingolipid metabolic process; F:hydrolase activity; P:regulation of cellular process
GSSPFG00029293001.4-PA	glutathione s-transferase	2	P:GO:0008152; F:GO:0016740	P:metabolic process; F:transferase activity
GSSPFG00031187001.6-PA	glutathione s-transferase	2	F:GO:0004364; P:GO:0008152	F:glutathione transferase activity; P:metabolic process
GSSPFG00031188001.6-PA	glutathione s-transferase	2	F:GO:0004364; P:GO:0008152	F:glutathione transferase activity; P:metabolic process
GSSPFG00028139001-PA	glyceraldehyde-3-phosphate dehydrogenase	6	F:GO:0004365; P:GO:0006006; P:GO:0006096; F:GO:0050661; F:GO:0051287; P:GO:0055114	F:glyceraldehyde-3-phosphate dehydrogenase (NAD+) (phosphorylating) activity; P:glucose metabolic process; P:glycolytic process; F:NADP binding; F:NAD binding; P:oxidation-reduction process
GSSPFG00007681001-PA	glyceraldehyde-3-phosphate dehydrogenase	6	F:GO:0004365; P:GO:0006006; P:GO:0006096; F:GO:0050661; F:GO:0051287; P:GO:0055114	F:glyceraldehyde-3-phosphate dehydrogenase (NAD+) (phosphorylating) activity; P:glucose metabolic process; P:glycolytic process; F:NADP binding; F:NAD binding; P:oxidation-reduction process
GSSPFG00004198001.1-PA	hatching enzyme-like protein	3	F:GO:0008237; P:GO:0022008; F:GO:0046872	F:metallopeptidase activity; P:neurogenesis; F:metal ion binding
GSSPFG00019658001.3-PA	heat shock protein 70	2	F:GO:0005524; C:GO:0005634	F:ATP binding; C:nucleus
GSSPFG00006887001.3-PA	hemocytin	1	F:GO:0005488	F:binding
GSSPFG00033211001-PA	hydroxybutyrate dehydrogenase	2	F:GO:0016491; P:GO:0055114	F:oxidoreductase activity; P:oxidation-reduction process
GSSPFG00031044001-PA	imaginal disc growth factor	4	F:GO:0004568; C:GO:0005576; P:GO:0005975; P:GO:0006032	F:chitinase activity; C:extracellular region; P:carbohydrate metabolic process; P:chitin catabolic process
GSSPFG00031848001-PA	insect intestinal mucin 4	3	C:GO:0005576; P:GO:0006030; F:GO:0008061	C:extracellular region; P:chitin metabolic process; F:chitin binding
GSSPFG00012306001-PA	intestinal mucin	3	C:GO:0005576; P:GO:0006030; F:GO:0008061	C:extracellular region; P:chitin metabolic process; F:chitin binding
GSSPFG00034251001-PA	intestinal mucin	3	C:GO:0005576; P:GO:0006030; F:GO:0008061	C:extracellular region; P:chitin metabolic process; F:chitin binding
GSSPFG00000143001-PA	intestinal mucin	3	C:GO:0005576; P:GO:0006030; F:GO:0008061	C:extracellular region; P:chitin metabolic process; F:chitin binding
GSSPFG00032729001.1-PA	lipase	3	F:GO:0004806; C:GO:0005576; P:GO:0008152	F:triglyceride lipase activity; C:extracellular region; P:metabolic process
GSSPFG00032730001-PA	lipase	3	C:GO:0005576; P:GO:0008152; F:GO:0052689	C:extracellular region; P:metabolic process; F:carboxylic ester hydrolase activity
GSSPFG00019149001.1-PA	lipase 1-like	2	P:GO:0016042; F:GO:0016788	P:lipid catabolic process; F:hydrolase activity, acting on ester bonds
GSSPFG00022875001-PA	lipase 3-like	2	P:GO:0016042; F:GO:0016788	P:lipid catabolic process; F:hydrolase activity, acting on ester bonds
GSSPFG00030786001-PA	lipoprotein lipase-like	3	C:GO:0005576; P:GO:0008152; F:GO:0052689	C:extracellular region; P:metabolic process; F:carboxylic ester hydrolase activity
GSSPFG00030535001-PA	methionine-rich storage protein	2	C:GO:0005615; F:GO:0045735	C:extracellular space; F:nutrient reservoir activity
GSSPFG00021328001-PA	methionine-rich storage protein	2	C:GO:0005615; F:GO:0045735	C:extracellular space; F:nutrient reservoir activity
GSSPFG00001760001.1-PA	mitochondrial aldehyde dehydrogenase	4	F:GO:0004029; C:GO:0005759; P:GO:0006090; P:GO:0055114	F:aldehyde dehydrogenase (NAD) activity; C:mitochondrial matrix; P:pyruvate metabolic process; P:oxidation-reduction process
GSSPFG00028320001-PA	moderately methionine rich storage protein	1	F:GO:0045735	F:nutrient reservoir activity
GSSPFG00028322001-PA	moderately methionine rich storage protein	1	F:GO:0045735	F:nutrient reservoir activity
GSSPFG00007022001-PA	muscle	10	F:GO:0004452; F:GO:0005200; F:GO:0005524; C:GO:0005737; C:GO:0005884; P:GO:0006909; P:GO:0008299; P:GO:0014866; F:GO:0016787; P:GO:0071689	F:isopentenyl-diphosphate delta-isomerase activity; F:structural constituent of cytoskeleton; F:ATP binding; C:cytoplasm; C:actin filament; P:phagocytosis; P:isoprenoid biosynthetic process; P:skeletal myofibril assembly; F:hydrolase activity; P:muscle thin filament assembly
GSSPFG00002079001-PA	muscle	6	F:GO:0004452; F:GO:0005524; C:GO:0005737; C:GO:0005856; P:GO:0008299; F:GO:0016787	F:isopentenyl-diphosphate delta-isomerase activity; F:ATP binding; C:cytoplasm; C:cytoskeleton; P:isoprenoid biosynthetic process; F:hydrolase activity
GSSPFG00030827001-PA	myosin-ib isoform x2	1	F:GO:0000166	F:nucleotide binding
GSSPFG00005110001-PA	neutral lipase	3	F:GO:0004806; C:GO:0005576; P:GO:0008152	F:triglyceride lipase activity; C:extracellular region; P:metabolic process
GSSPFG00005485001.1-PA	neutral lipase	3	F:GO:0004806; C:GO:0005576; P:GO:0008152	F:triglyceride lipase activity; C:extracellular region; P:metabolic process
GSSPFG00006266001-PA	pancreatic triacylglycerol lipase-like	3	C:GO:0005576; P:GO:0008152; F:GO:0052689	C:extracellular region; P:metabolic process; F:carboxylic ester hydrolase activity
GSSPFG00024193001-PA	pancreatic triacylglycerol lipase-like	3	C:GO:0005576; P:GO:0008152; F:GO:0052689	C:extracellular region; P:metabolic process; F:carboxylic ester hydrolase activity
GSSPFG00009950001-PA	pancreatic triacylglycerol lipase-like	3	C:GO:0005576; P:GO:0008152; F:GO:0052689	C:extracellular region; P:metabolic process; F:carboxylic ester hydrolase activity
GSSPFG00005109001-PA	pancreatic triacylglycerol lipase-like	3	C:GO:0005576; P:GO:0008152; F:GO:0052689	C:extracellular region; P:metabolic process; F:carboxylic ester hydrolase activity
GSSPFG00003910001-PA	peptidyl-prolyl cis-trans isomerase 5	3	P:GO:0000413; F:GO:0003755; P:GO:0006457	P:protein peptidyl-prolyl isomerization; F:peptidyl-prolyl cis-trans isomerase activity; P:protein folding
GSSPFG00031847001-PA	peritrophic matrix insect intestinal mucin	3	C:GO:0005576; P:GO:0006030; F:GO:0008061	C:extracellular region; P:chitin metabolic process; F:chitin binding
GSSPFG00013728001-PA	peritrophic membrane chitin binding protein	3	P:GO:0005975; F:GO:0016810; F:GO:0051060	P:carbohydrate metabolic process; F:hydrolase activity, acting on carbon-nitrogen (but not peptide) bonds; F:pullulanase activity
GSSPFG00016558001.1-PA	peritrophic membrane chitin binding protein	3	P:GO:0005975; F:GO:0016810; F:GO:0051060	P:carbohydrate metabolic process; F:hydrolase activity, acting on carbon-nitrogen (but not peptide) bonds; F:pullulanase activity
GSSPFG00015718001.1-PA	polycomb protein suz12-b isoform x1	4	F:GO:0003756; C:GO:0005783; P:GO:0006457; P:GO:0045454	F:protein disulfide isomerase activity; C:endoplasmic reticulum; P:protein folding; P:cell redox homeostasis

GSSPFG00021013001.3-PA	prophenoloxidase subunit 1	7	F:GO:0004503; F:GO:0005507; C:GO:0005576; P:GO:0006583; F:GO:0031404; P:GO:0035008; P:GO:0055114	F:monophenol monooxygenase activity; F:copper ion binding; C:extracellular region; P:melanin biosynthetic process from tyrosine; F:chloride ion binding; P:positive regulation of melanization defense response; P:oxidation-reduction process
GSSPFG00012369001-PA	prophenoloxidase subunit 1	7	F:GO:0004503; F:GO:0005507; C:GO:0005576; P:GO:0006583; F:GO:0031404; P:GO:0035008; P:GO:0055114	F:monophenol monooxygenase activity; F:copper ion binding; C:extracellular region; P:melanin biosynthetic process from tyrosine; F:chloride ion binding; P:positive regulation of melanization defense response; P:oxidation-reduction process
GSSPFG00013976001.3-PA	prophenoloxidase subunit 2	7	F:GO:0004503; F:GO:0005507; C:GO:0005576; P:GO:0006583; F:GO:0031404; P:GO:0035008; P:GO:0055114	F:monophenol monooxygenase activity; F:copper ion binding; C:extracellular region; P:melanin biosynthetic process from tyrosine; F:chloride ion binding; P:positive regulation of melanization defense response; P:oxidation-reduction process
GSSPFG00034655001.1-PA	protein disulfide-isomerase	4	F:GO:0003756; C:GO:0005783; P:GO:0006457; P:GO:0045454	F:protein disulfide isomerase activity; C:endoplasmic reticulum; P:protein folding; P:cell redox homeostasis
GSSPFG00003602001.1-PA	protein disulfide-isomerase	4	F:GO:0003756; C:GO:0005783; P:GO:0006457; P:GO:0045454	F:protein disulfide isomerase activity; C:endoplasmic reticulum; P:protein folding; P:cell redox homeostasis
GSSPFG00025002001-PA	pyruvate kinase	5	F:GO:0000287; F:GO:0004743; P:GO:0006096; F:GO:0016301; F:GO:0030955	F:magnesium ion binding; F:pyruvate kinase activity; P:glycolytic process; F:kinase activity; F:potassium ion binding
GSSPFG00021735001.1-PA	pyruvate kinase-like isoform x1	5	F:GO:0000287; F:GO:0004743; P:GO:0006096; F:GO:0016301; F:GO:0030955	F:magnesium ion binding; F:pyruvate kinase activity; P:glycolytic process; F:kinase activity; F:potassium ion binding
GSSPFG00009143001-PA	retinal dehydrogenase 1	4	F:GO:0001758; F:GO:0004028; F:GO:0018479; P:GO:0055114	F:retinal dehydrogenase activity; F:3-chloroallyl aldehyde dehydrogenase activity; F:benzaldehyde dehydrogenase (NAD+) activity; P:oxidation-reduction process
GSSPFG00035967001.2-PA	serine protease	2	F:GO:0004252; P:GO:0006508	F:serine-type endopeptidase activity; P:proteolysis
GSSPFG00002248001.3-PA	serine protease 11	2	F:GO:0004252; P:GO:0006508	F:serine-type endopeptidase activity; P:proteolysis
GSSPFG00020781001.3-PA	serine protease 11	2	F:GO:0004252; P:GO:0006508	F:serine-type endopeptidase activity; P:proteolysis
GSSPFG00000365001.3-PA	serine protease 2	2	F:GO:0004252; P:GO:0006508	F:serine-type endopeptidase activity; P:proteolysis
GSSPFG00033017001.3-PA	serine protease 33	2	F:GO:0004252; P:GO:0006508	F:serine-type endopeptidase activity; P:proteolysis
GSSPFG00028610001.3-PA	serine protease 33	1	F:GO:0008236	F:serine-type peptidase activity
GSSPFG00033041001.2-PA	serine protease 38	1	F:GO:0008233	F:peptidase activity
GSSPFG00025116001.3-PA	serine protease 40	2	F:GO:0005515; F:GO:0008236	F:protein binding; F:serine-type peptidase activity
GSSPFG00014594001.3-PA	serine protease 40	3	F:GO:0004252; F:GO:0005515; P:GO:0006508	F:serine-type endopeptidase activity; F:protein binding; P:proteolysis
GSSPFG00028801001-PA	serine protease snake-like	1	F:GO:0016787	F:hydrolase activity
GSSPFG00009353001-PA	serine protease snake-like	1	F:GO:0008233	F:peptidase activity
GSSPFG00012894001-PA	serpin 1	4	C:GO:0005615; F:GO:0008233; P:GO:0010466; F:GO:0030414	C:extracellular space; F:peptidase activity; P:negative regulation of peptidase activity; F:peptidase inhibitor activity
GSSPFG00027555001-PA	thymus-specific serine protease	3	F:GO:0004180; P:GO:0006508; F:GO:0008236	F:carboxypeptidase activity; P:proteolysis; F:serine-type peptidase activity
GSSPFG00018186001.2-PA	triosephosphate isomerase	8	F:GO:0003735; F:GO:0004807; C:GO:0005840; P:GO:0006094; P:GO:0006096; P:GO:0006098; P:GO:0006412; F:GO:0019843	F:structural constituent of ribosome; F:triose-phosphate isomerase activity; C:ribosome; P:gluconeogenesis; P:glycolytic process; P:pentose-phosphate shunt; P:translation; F:rRNA binding
GSSPFG00018186001.2-PB	triosephosphate isomerase	8	F:GO:0003735; F:GO:0004807; C:GO:0005840; P:GO:0006094; P:GO:0006096; P:GO:0006098; P:GO:0006412; F:GO:0019843	F:structural constituent of ribosome; F:triose-phosphate isomerase activity; C:ribosome; P:gluconeogenesis; P:glycolytic process; P:pentose-phosphate shunt; P:translation; F:rRNA binding
GSSPFG00001201001.2-PA	trypsin ia2	1	F:GO:0008236	F:serine-type peptidase activity
GSSPFG00035969001.2-PA	trypsin ia2	2	F:GO:0004252; P:GO:0006508	F:serine-type endopeptidase activity; P:proteolysis
GSSPFG00034101001.3-PA	trypsin precursor 9	2	F:GO:0004252; P:GO:0006508	F:serine-type endopeptidase activity; P:proteolysis
GSSPFG00005080001.4-PA	trypsin precursor 9	2	F:GO:0004252; P:GO:0006508	F:serine-type endopeptidase activity; P:proteolysis
GSSPFG00019625001.2-PA	trypsin-like protease	2	F:GO:0004252; P:GO:0006508	F:serine-type endopeptidase activity; P:proteolysis
GSSPFG00012657001.3-PA	trypsin-like protease	2	F:GO:0004252; P:GO:0006508	F:serine-type endopeptidase activity; P:proteolysis
GSSPFG00007297001.3-PA	trypsin-like protease	2	F:GO:0004252; P:GO:0006508	F:serine-type endopeptidase activity; P:proteolysis
GSSPFG00032515001.3-PA	trypsin-like protease	1	F:GO:0016787	F:hydrolase activity
GSSPFG00021205001.4-PA	trypsin-like protease	2	F:GO:0004252; P:GO:0006508	F:serine-type endopeptidase activity; P:proteolysis
GSSPFG00013283001.6-PB	trypsin-like protease	2	F:GO:0004252; P:GO:0006508	F:serine-type endopeptidase activity; P:proteolysis
GSSPFG00013282001.6-PA	trypsin-like protease	2	F:GO:0004252; P:GO:0006508	F:serine-type endopeptidase activity; P:proteolysis
GSSPFG00012077001.4-PA	trypsin-like serine partial	2	F:GO:0004252; P:GO:0006508	F:serine-type endopeptidase activity; P:proteolysis
GSSPFG00028489001.5-PB	trypsin-like serine proteinase t26	3	F:GO:0004252; F:GO:0005515; P:GO:0006508	F:serine-type endopeptidase activity; F:protein binding; P:proteolysis
GSSPFG00028708001.1-PA	uncharacterized protein loc100878113	3	P:GO:0006413; C:GO:0032991; C:GO:0044444	P:translational initiation; C:protein-containing complex; C:cytoplasmic part
GSSPFG00027141001-PA	v-type proton atpase catalytic subunit a	5	F:GO:0005524; P:GO:0015991; C:GO:0033180; P:GO:0046034; F:GO:0046961	F:ATP binding; P:ATP hydrolysis coupled proton transport; C:proton-transporting V-type ATPase, V1 domain; P:ATP metabolic process; F:proton-transporting ATPase activity, rotational mechanism
GSSPFG00034314001-PA	xaa-pro aminopeptidase 1	2	P:GO:0008152; F:GO:0016787	P:metabolic process; F:hydrolase activity
GSSPFG00018804001.1-PA	zinc carboxypeptidase-like	1	F:GO:0004180	F:carboxypeptidase activity
GSSPFG00017650001-PA	zinc carboxypeptidase-like	2	F:GO:0004180; F:GO:0046872	F:carboxypeptidase activity; F:metal ion binding
