# Supplementary material for: Interaction of a Densovirus with Glycans of the Peritrophic Matrix Mediates Oral Infection of the Lepidopteran Pest Spodoptera frugiperda
Source: Viruses. 2019 Sep 17;11(9):870. doi: 10.3390/v11090870 (PMC6783882; doi:10.3390/v11090870)
Supplement: Supplementary file 1 [file viruses-11-00870-s001.zip › Supplementary figures and Tables/Supp Table2.pdf]

DGE libraries	Number of Tags
Day 1 Mock	605378
Day 1 JcDV	1753522
Day 3 Mock	640390
Day 3 JcDV	638945
