# Supplementary material for: Interaction of a Densovirus with Glycans of the Peritrophic Matrix Mediates Oral Infection of the Lepidopteran Pest Spodoptera frugiperda
Source: Viruses. 2019 Sep 17;11(9):870. doi: 10.3390/v11090870 (PMC6783882; doi:10.3390/v11090870)
Supplement: Supplementary file 1 [file viruses-11-00870-s001.zip › Supplementary figures and Tables/Supp Table3.pdf]

Number of	Day 1 (Mock/JcDV)	Day 3 (Mock/JcDV)
Unique Tags (*P* value<0.001)	8586	6317
Tags differentially represented between libraries (fold change ≥ 5)	190	196
Up-represented transcripts in JcDV condition	66	130
Down-represented transcripts in JcDV condition	28	32
