# Supplementary material for: Interaction of a Densovirus with Glycans of the Peritrophic Matrix Mediates Oral Infection of the Lepidopteran Pest Spodoptera frugiperda
Source: Viruses. 2019 Sep 17;11(9):870. doi: 10.3390/v11090870 (PMC6783882; doi:10.3390/v11090870)
Supplement: Supplementary file 1 [file viruses-11-00870-s001.zip › Supplementary figures and Tables/Supp Table4_A.pdf]

Name	Description	#GO	GO IDs	GO Names
GSSPFG00023569001-PA	acyl-binding protein	1	F:GO:0000062	F:fatty-acyl-CoA binding
GSSPFG00019131001-PA	aminopeptidase n	5	F:GO:0004177; P:GO:0006508; F:GO:0008237; F:GO:0008270; C:GO:0016020	F:aminopeptidase activity; P:proteolysis; F:metallopeptidase activity; F:zinc ion binding; C:membrane
GSSPFG00016399001.2-PA	d-arabinitol dehydrogenase 1-like	3	F:GO:0008270; F:GO:0016491; P:GO:0055114	F:zinc ion binding; F:oxidoreductase activity; P:oxidation-reduction process
GSSPFG00030737001-PA	e3 ubiquitin-protein ligase siah1-like	7	F:GO:0004842; C:GO:0005634; P:GO:0006511; P:GO:0007275; F:GO:0008270; P:GO:0016567; F:GO:0016874	F:ubiquitin-protein transferase activity; C:nucleus; P:ubiquitin-dependent protein catabolic process; P:multicellular organism development; F:zinc ion binding; P:protein ubiquitination; F:ligase activity
GSSPFG00014785001-PA	eukaryotic translation initiation factor 3 subunit g	8	F:GO:0000166; P:GO:0001731; F:GO:0003743; C:GO:0005852; P:GO:0006446; F:GO:0008270; C:GO:0016282; C:GO:0033290	F:nucleotide binding; P:formation of translation preinitiation complex; F:translation initiation factor activity; C:eukaryotic translation initiation factor 3 complex; P:regulation of translational initiation; F:zinc ion binding; C:eukaryotic 43S preinitiation complex; C:eukaryotic 48S preinitiation complex
GSSPFG00002382001-PA	facilitated trehalose transporter tret1-2 homolog	5	F:GO:0005355; C:GO:0005886; C:GO:0016021; P:GO:0046427; P:GO:0055085	F:glucose transmembrane transporter activity; C:plasma membrane; C:integral component of membrane; P:positive regulation of JAK-STAT cascade; P:transmembrane transport
GSSPFG00034701001-PA	fatty acid binding protein	3	F:GO:0005215; P:GO:0006810; F:GO:0008289	F:transporter activity; P:transport; F:lipid binding
GSSPFG00034702001-PA	fatty acid binding protein	3	F:GO:0005215; P:GO:0006810; F:GO:0008289	F:transporter activity; P:transport; F:lipid binding
GSSPFG00026492001.1-PA	glucosylceramidase-like	3	P:GO:0006665; F:GO:0016787; P:GO:0050794	P:sphingolipid metabolic process; F:hydrolase activity; P:regulation of cellular process
GSSPFG00027388001-PA	hydroxyacyl-coenzyme a dehydrogenase	4	F:GO:0003857; F:GO:0004300; P:GO:0006635; C:GO:0016507	F:3-hydroxyacyl-CoA dehydrogenase activity; F:enoyl-CoA hydratase activity; P:fatty acid beta-oxidation; C:mitochondrial fatty acid beta-oxidation multienzyme complex
GSSPFG00002672001-PA	inorganic pyrophosphatase	4	F:GO:0000287; F:GO:0004427; C:GO:0005737; P:GO:0006796	F:magnesium ion binding; F:inorganic diphosphatase activity; C:cytoplasm; P:phosphate-containing compound metabolic process
GSSPFG00032730001-PA	lipase	3	C:GO:0005576; P:GO:0008152; F:GO:0052689	C:extracellular region; P:metabolic process; F:carboxylic ester hydrolase activity
GSSPFG00028977001-PA	nitric oxide synthase	9	F:GO:0004517; F:GO:0005506; F:GO:0005516; P:GO:0006809; F:GO:0010181; F:GO:0020037; F:GO:0050660; F:GO:0050661; P:GO:0055114	F:nitric-oxide synthase activity; F:iron ion binding; F:calmodulin binding; P:nitric oxide biosynthetic process; F:FMN binding; F:heme binding; F:flavin adenine dinucleotide binding; F:NADP binding; P:oxidation-reduction process
GSSPFG00019809001-PA	nose resistant to fluoxetine protein 6-like	2	P:GO:0008152; F:GO:0016747	P:metabolic process; F:transferase activity, transferring acyl groups other than amino-acyl groups
GSSPFG00005109001-PA	pancreatic triacylglycerol lipase-like	3	C:GO:0005576; P:GO:0008152; F:GO:0052689	C:extracellular region; P:metabolic process; F:carboxylic ester hydrolase activity
GSSPFG00009950001-PA	pancreatic triacylglycerol lipase-like	3	C:GO:0005576; P:GO:0008152; F:GO:0052689	C:extracellular region; P:metabolic process; F:carboxylic ester hydrolase activity
GSSPFG00034748001-PA	parathyroid hormone-responsive b1	1	C:GO:0034464	C:BBSome
GSSPFG00009935001.1-PA	protein aatf	1	C:GO:0005634	C:nucleus
GSSPFG00008671001-PA	protein rop isoform x1	2	C:GO:0005623; P:GO:0006904	C:cell; P:vesicle docking involved in exocytosis
GSSPFG00015295001-PA	rna polymerase ii transcriptional coactivator	3	F:GO:0003677; F:GO:0003713; P:GO:0006355	F:DNA binding; F:transcription coactivator activity; P:regulation of transcription, DNA-templated
GSSPFG00018170001-PA	sterol carrier protein 2 3-oxoacyl- thiolase	7	F:GO:0005548; C:GO:0005739; C:GO:0005777; C:GO:0005811; P:GO:0008152; P:GO:0015914; F:GO:0016747	F:phospholipid transporter activity; C:mitochondrion; C:peroxisome; C:lipid droplet; P:metabolic process; P:phospholipid transport; F:transferase activity, transferring acyl groups other than amino-acyl groups
GSSPFG00027537001-PA	transmembrane protein 14c	1	C:GO:0016021	C:integral component of membrane
GSSPFG00010017001-PA	trna pseudouridine synthase mitochondrial-like isoform x1	5	P:GO:0001522; F:GO:0003723; F:GO:0004730; P:GO:0008033; F:GO:0009982	P:pseudouridine synthesis; F:RNA binding; F:pseudouridylate synthase activity; P:tRNA processing; F:pseudouridine synthase activity
GSSPFG00030048001.3-PA	trypsin-like serine protease	2	F:GO:0004252; P:GO:0006508	F:serine-type endopeptidase activity; P:proteolysis
GSSPFG00027850001-PA	uncharacterized peptidase c1-like protein isoform x2	6	F:GO:0005044; P:GO:0006508; P:GO:0006898; P:GO:0006955; F:GO:0008234; F:GO:0030247	F:scavenger receptor activity; P:proteolysis; P:receptor-mediated endocytosis; P:immune response; F:cysteine-type peptidase activity; F:polysaccharide binding
GSSPFG00012410001-PA	uncharacterized protein loc101739891	1	C:GO:0016020	C:membrane
GSSPFG00034417001-PA	uv opsin	7	F:GO:0004930; P:GO:0007186; P:GO:0007601; P:GO:0007602; F:GO:0009881; C:GO:0016021; P:GO:0018298	F:G protein-coupled receptor activity; P:G protein-coupled receptor signaling pathway; P:visual perception; P:phototransduction; F:photoreceptor activity; C:integral component of membrane; P:protein-chromophore linkage
GSSPFG00014231001-PA	v-type proton atpase subunit e	4	P:GO:0008152; P:GO:0015991; C:GO:0033178; F:GO:0046961	P:metabolic process; P:ATP hydrolysis coupled proton transport; C:proton-transporting two-sector ATPase complex, catalytic domain; F:proton-transporting ATPase activity, rotational mechanism
