# Supplementary material for: Interaction of a Densovirus with Glycans of the Peritrophic Matrix Mediates Oral Infection of the Lepidopteran Pest Spodoptera frugiperda
Source: Viruses. 2019 Sep 17;11(9):870. doi: 10.3390/v11090870 (PMC6783882; doi:10.3390/v11090870)
Supplement: Supplementary file 1 [file viruses-11-00870-s001.zip › Supplementary figures and Tables/Supp Table4_B.pdf]

Name	Description	#GO	GO IDs	GO Names
GSSPFG00022879001-PA	14-3-3 protein zeta isoform x1	2	C:GO:0005737; F:GO:0019904	C:cytoplasm; F:protein domain specific binding
GSSPFG00006068001-PA	40s ribosomal protein s11	3	F:GO:0003735; C:GO:0005840; P:GO:0006412	F:structural constituent of ribosome; C:ribosome; P:translation
GSSPFG00003276001-PA	40s ribosomal protein s14	3	F:GO:0003735; P:GO:0006412; C:GO:0022627	F:structural constituent of ribosome; P:translation; C:cytosolic small ribosomal subunit
GSSPFG00019839001-PA	40s ribosomal protein s23	4	F:GO:0003677; F:GO:0003735; P:GO:0006412; C:GO:0015935	F:DNA binding; F:structural constituent of ribosome; P:translation; C:small ribosomal subunit
GSSPFG00014527001-PA	60s ribosomal protein l10a	4	F:GO:0003723; F:GO:0003735; P:GO:0006412; C:GO:0015934	F:RNA binding; F:structural constituent of ribosome; P:translation; C:large ribosomal subunit
GSSPFG00007050001-PA	60s ribosomal protein l10a	4	F:GO:0003723; F:GO:0003735; P:GO:0006412; C:GO:0015934	F:RNA binding; F:structural constituent of ribosome; P:translation; C:large ribosomal subunit
GSSPFG00012069001.3-PA	actin-related protein 3	21	F:GO:0003779; F:GO:0005200; F:GO:0005524; C:GO:0005884; C:GO:0005885; P:GO:0006886; P:GO:0006909; P:GO:0007413; P:GO:0007520; P:GO:0010592; P:GO:0022416; C:GO:0030027; P:GO:0030037; P:GO:0030589; P:GO:0030713; P:GO:0034314; P:GO:0045747; P:GO:0045887; P:GO:0051491; P:GO:0072553; P:GO:0097320	F:actin binding; F:structural constituent of cytoskeleton; F:ATP binding; C:actin filament; C:Arp2/3 protein complex; P:intracellular protein transport; P:phagocytosis; P:axonal fasciculation; P:myoblast fusion; P:positive regulation of lamellipodium assembly; P:chaeta development; C:lamellipodium; P:actin filament reorganization involved in cell cycle; P:pseudocleavage involved in syncytial blastoderm formation; P:ovarian follicle cell stalk formation; P:Arp2/3 complex-mediated actin nucleation; P:positive regulation of Notch signaling pathway; P:positive regulation of synaptic growth at neuromuscular junction; P:positive regulation of filopodium assembly; P:terminal button organization; P:plasma membrane tubulation
GSSPFG00014383001-PA	aldo-keto reductase	2	F:GO:0016491; P:GO:0055114	F:oxidoreductase activity; P:oxidation-reduction process
GSSPFG00004769001-PA	cadherin-87a	2	P:GO:0007155; C:GO:0016020	P:cell adhesion; C:membrane
GSSPFG00002450001-PA	carboxylesterase	2	P:GO:0008152; F:GO:0016787	P:metabolic process; F:hydrolase activity
GSSPFG00030094001-PA	carboxypeptidase b-like	1	F:GO:0004180	F:carboxypeptidase activity
GSSPFG00006714001-PA	catalase	13	F:GO:0004096; F:GO:0005515; P:GO:0006979; P:GO:0009987; F:GO:0020037; C:GO:0043231; P:GO:0044238; P:GO:0044260; C:GO:0044444; F:GO:0046872; P:GO:0050794; P:GO:0055114; P:GO:1901360	F:catalase activity; F:protein binding; P:response to oxidative stress; P:cellular process; F:heme binding; C:intracellular membrane-bounded organelle; P:primary metabolic process; P:cellular macromolecule metabolic process; C:cytoplasmic part; F:metal ion binding; P:regulation of cellular process; P:oxidation-reduction process; P:organic cyclic compound metabolic process
GSSPFG00030477001.4-PA	catalase	29	F:GO:0000981; F:GO:0001158; P:GO:0003007; F:GO:0004096; F:GO:0004102; C:GO:0005634; C:GO:0005777; C:GO:0005891; P:GO:0007274; P:GO:0007517; P:GO:0008292; F:GO:0008332; P:GO:0008340; P:GO:0016339; F:GO:0020037; P:GO:0035206; P:GO:0038001; P:GO:0042542; P:GO:0042673; P:GO:0042682; P:GO:0042744; P:GO:0045471; P:GO:0045676; P:GO:0045944; F:GO:0046872; P:GO:0048813; P:GO:0055114; P:GO:0070509; P:GO:0070588	F:DNA-binding transcription factor activity, RNA polymerase II-specific; F:enhancer sequence-specific DNA binding; P:heart morphogenesis; F:catalase activity; F:choline O-acetyltransferase activity; C:nucleus; C:peroxisome; C:voltage-gated calcium channel complex; P:neuromuscular synaptic transmission; P:muscle organ development; P:acetylcholine biosynthetic process; F:low voltage-gated calcium channel activity; P:determination of adult lifespan; P:calcium-dependent cell-cell adhesion via plasma membrane cell adhesion molecules; F:heme binding; P:regulation of hemocyte proliferation; P:paracrine signaling; P:response to hydrogen peroxide; P:regulation of retinal cone cell fate specification; P:regulation of compound eye cone cell fate specification; P:hydrogen peroxide catabolic process; P:response to ethanol; P:regulation of R7 cell differentiation; P:positive regulation of transcription by RNA polymerase II; F:metal ion binding; P:dendrite morphogenesis; P:oxidation-reduction process; P:calcium ion import; P:calcium ion transmembrane transport
GSSPFG00006818001.1-PA	cd63 antigen	1	C:GO:0016021	C:integral component of membrane
GSSPFG00004358001-PA	cell cycle checkpoint control protein rad9a	3	P:GO:0000077; P:GO:0006281; C:GO:0030896	P:DNA damage checkpoint; P:DNA repair; C:checkpoint clamp complex
GSSPFG00008227001.3-PA	chymotrypsin-like protease	2	F:GO:0004252; P:GO:0006508	F:serine-type endopeptidase activity; P:proteolysis
GSSPFG00030121001.3-PA	chymotrypsin-like protease	2	F:GO:0004252; P:GO:0006508	F:serine-type endopeptidase activity; P:proteolysis
GSSPFG00034359001.3-PA	chymotrypsin-like serine protease	2	F:GO:0004252; P:GO:0006508	F:serine-type endopeptidase activity; P:proteolysis
GSSPFG00003632001-PA	complement component 1 q subcomponent-binding mitochondrial	1	C:GO:0005759	C:mitochondrial matrix
GSSPFG00022978001.3-PA	cysteine protease atg4d isoform x2	5	C:GO:0005737; P:GO:0006508; P:GO:0006914; F:GO:0008234; P:GO:0015031	C:cytoplasm; P:proteolysis; P:autophagy; F:cysteine-type peptidase activity; P:protein transport
GSSPFG00019339001-PA	delta-aminolevulinic acid dehydratase isoform x2	4	F:GO:0004655; F:GO:0005524; P:GO:0006779; F:GO:0046872	F:porphobilinogen synthase activity; F:ATP binding; P:porphyrin-containing compound biosynthetic process; F:metal ion binding
GSSPFG00009074001-PA	deoxycytidylate deaminase	4	F:GO:0004132; P:GO:0009117; F:GO:0046872; C:GO:0070062	F:dCMP deaminase activity; P:nucleotide metabolic process; F:metal ion binding; C:extracellular exosome
GSSPFG00020627001-PA	dj-1 beta	5	P:GO:0008344; P:GO:0042775; P:GO:0046626; P:GO:0048640; P:GO:0070301	P:adult locomotory behavior; P:mitochondrial ATP synthesis coupled electron transport; P:regulation of insulin receptor signaling pathway; P:negative regulation of developmental growth; P:cellular response to hydrogen peroxide
GSSPFG00025486001.2-PA	dna (cytosine-5)-methyltransferase -like isoform x1	6	F:GO:0003677; F:GO:0003682; F:GO:0003886; C:GO:0005634; F:GO:0008270; P:GO:0090116	F:DNA binding; F:chromatin binding; F:DNA (cytosine-5-)-methyltransferase activity; C:nucleus; F:zinc ion binding; P:C-5 methylation of cytosine
GSSPFG00005701001-PA	dopamine n-acetyltransferase-like	2	F:GO:0008080; P:GO:0008152	F:N-acetyltransferase activity; P:metabolic process
GSSPFG00033485001-PA	elongation factor 1 delta	3	F:GO:0003746; C:GO:0005853; P:GO:0006414	F:translation elongation factor activity; C:eukaryotic translation elongation factor 1 complex; P:translational elongation
GSSPFG00022059001-PA	glucosamine-6-phosphate isomerase-like isoform x1	7	F:GO:0004342; C:GO:0005737; P:GO:0005975; P:GO:0006043; P:GO:0006044; P:GO:0006091; F:GO:0016787	F:glucosamine-6-phosphate deaminase activity; C:cytoplasm; P:carbohydrate metabolic process; P:glucosamine catabolic process; P:N-acetylglucosamine metabolic process; P:generation of precursor metabolites and energy; F:hydrolase activity
GSSPFG00009170001.5-PA	glutathione s-transferase theta-1-like	2	F:GO:0004364; P:GO:0008152	F:glutathione transferase activity; P:metabolic process
GSSPFG00007670001-PA	growth arrest and dna damage-inducible protein gadd45 alpha-like	3	C:GO:0005634; P:GO:0006950; P:GO:0051726	C:nucleus; P:response to stress; P:regulation of cell cycle
GSSPFG00021491001.3-PA	growth hormone-inducible transmembrane	1	C:GO:0016021	C:integral component of membrane
GSSPFG00002254001-PA	haspin-like protein	3	F:GO:0004672; F:GO:0005524; P:GO:0006468	F:protein kinase activity; F:ATP binding; P:protein phosphorylation
GSSPFG00004200001.1-PA	hatching enzyme-like protein	3	F:GO:0004222; P:GO:0006508; F:GO:0008270	F:metalloendopeptidase activity; P:proteolysis; F:zinc ion binding

GSSPFG00015955001.3-PA	heat shock protein	1	F:GO:0005212	F:structural constituent of eye lens
GSSPFG00026171001-PA	high affinity copper uptake protein 1 isoform x2	3	F:GO:0005375; C:GO:0016021; P:GO:0035434	F:copper ion transmembrane transporter activity; C:integral component of membrane; P:copper ion transmembrane transport
GSSPFG00028706001-PA	histone	4	C:GO:0000786; F:GO:0003677; C:GO:0005634; F:GO:0046982	C:nucleosome; F:DNA binding; C:nucleus; F:protein heterodimerization activity
GSSPFG00031885001-PA	ischemia reperfusion inducible protein	4	F:GO:0003725; P:GO:0006412; F:GO:0016740; F:GO:0045182	F:double-stranded RNA binding; P:translation; F:transferase activity; F:translation regulator activity
GSSPFG00009739001.1-PA	leukocyte receptor cluster member 4 protein	1	C:GO:0016021	C:integral component of membrane
GSSPFG00009826001-PA	map kinase-interacting serine threonine-protein kinase 1	11	F:GO:0004674; F:GO:0005524; C:GO:0005635; C:GO:0005813; C:GO:0005829; C:GO:0005874; P:GO:0006468; P:GO:0007017; P:GO:0040008; P:GO:0046580; F:GO:0051019	F:protein serine/threonine kinase activity; F:ATP binding; C:nuclear envelope; C:centrosome; C:cytosol; C:microtubule; P:protein phosphorylation; P:microtubule-based process; P:regulation of growth; P:negative regulation of Ras protein signal transduction; F:mitogen-activated protein kinase binding
GSSPFG00011895001-PA	mitochondrial dicarboxylate carrier	2	P:GO:0006810; C:GO:0016021	P:transport; C:integral component of membrane
GSSPFG00001220001-PA	organic cation transporter protein isoform x1	3	C:GO:0016021; F:GO:0022857; P:GO:0055085	C:integral component of membrane; F:transmembrane transporter activity; P:transmembrane transport
GSSPFG00003126001.3-PA	partial	3	F:GO:0003700; P:GO:0006355; F:GO:0043565	F:DNA-binding transcription factor activity; P:regulation of transcription, DNA-templated; F:sequence-specific DNA binding
GSSPFG00002429001-PA	pleckstrin homology domain-containing family m member 1	3	C:GO:0005622; F:GO:0008270; P:GO:0035556	C:intracellular; F:zinc ion binding; P:intracellular signal transduction
GSSPFG00031628001-PA	probable phosphoserine aminotransferase	2	F:GO:0004648; P:GO:0006564	F:O-phospho-L-serine:2-oxoglutarate aminotransferase activity; P:L-serine biosynthetic process
GSSPFG00013235001-PA	pyruvate mitochondrial isoform x1	8	F:GO:0003677; F:GO:0004075; F:GO:0004736; F:GO:0005524; P:GO:0006090; P:GO:0006094; F:GO:0009374; F:GO:0046872	F:DNA binding; F:biotin carboxylase activity; F:pyruvate carboxylase activity; F:ATP binding; P:pyruvate metabolic process; P:gluconeogenesis; F:biotin binding; F:metal ion binding
GSSPFG00020427001-PA	ribosomal protein l13	3	F:GO:0003735; C:GO:0005840; P:GO:0006412	F:structural constituent of ribosome; C:ribosome; P:translation
GSSPFG00024299001-PA	ribosomal protein l18a	3	F:GO:0003735; C:GO:0005840; P:GO:0006412	F:structural constituent of ribosome; C:ribosome; P:translation
GSSPFG00002705001.1-PA	ribosomal protein s2	4	F:GO:0003723; F:GO:0003735; P:GO:0006412; C:GO:0015935	F:RNA binding; F:structural constituent of ribosome; P:translation; C:small ribosomal subunit
GSSPFG00023951001-PA	serine threonine-protein phosphatase alpha-2 isoform	2	F:GO:0004721; P:GO:0006470	F:phosphoprotein phosphatase activity; P:protein dephosphorylation
GSSPFG00000284001-PA	serine threonine-protein phosphatase rdgc-like	6	F:GO:0004721; F:GO:0005506; F:GO:0005509; P:GO:0006470; F:GO:0030145; P:GO:0050906	F:phosphoprotein phosphatase activity; F:iron ion binding; F:calcium ion binding; P:protein dephosphorylation; F:manganese ion binding; P:detection of stimulus involved in sensory perception
GSSPFG00001324001.1-PA	s-formylglutathione hydrolase	4	C:GO:0005737; F:GO:0018738; C:GO:0043231; P:GO:0046294	C:cytoplasm; F:S-formylglutathione hydrolase activity; C:intracellular membrane-bounded organelle; P:formaldehyde catabolic process
GSSPFG00025156001-PA	signal peptidase complex subunit 3-like	4	C:GO:0005787; P:GO:0006465; F:GO:0008233; C:GO:0016021	C:signal peptidase complex; P:signal peptide processing; F:peptidase activity; C:integral component of membrane
GSSPFG00029082001.6-PA	small heat shock protein	2	F:GO:0005212; C:GO:0005840	F:structural constituent of eye lens; C:ribosome
GSSPFG00010112001-PA	succinyl-ligase	4	F:GO:0005524; P:GO:0008152; F:GO:0016874; F:GO:0046872	F:ATP binding; P:metabolic process; F:ligase activity; F:metal ion binding
GSSPFG00011227001-PA	synaptic vesicle glycoprotein 2b	4	F:GO:0008270; C:GO:0016021; F:GO:0022857; P:GO:0055085	F:zinc ion binding; C:integral component of membrane; F:transmembrane transporter activity; P:transmembrane transport
GSSPFG00007437001.3-PA	transcription factor kayak isoform x3	3	F:GO:0003700; P:GO:0006357; F:GO:0043565	F:DNA-binding transcription factor activity; P:regulation of transcription by RNA polymerase II; F:sequence-specific DNA binding
GSSPFG00020171001-PA	translocon-associated protein subunit beta	4	C:GO:0005789; F:GO:0008519; C:GO:0016021; P:GO:0072488	C:endoplasmic reticulum membrane; F:ammonium transmembrane transporter activity; C:integral component of membrane; P:ammonium transmembrane transport
GSSPFG00021205001.4-PA	trypsin-like protease	2	F:GO:0004252; P:GO:0006508	F:serine-type endopeptidase activity; P:proteolysis
GSSPFG00028708001.1-PA	uncharacterized protein loc100878113	3	P:GO:0006413; C:GO:0032991; C:GO:0044444	P:translational initiation; C:protein-containing complex; C:cytoplasmic part
GSSPFG00028709001.1-PA	uncharacterized protein loc100878113	3	P:GO:0006413; C:GO:0032991; C:GO:0044444	P:translational initiation; C:protein-containing complex; C:cytoplasmic part
GSSPFG00017701001-PA	vesicle transport protein got1b	2	P:GO:0006457; P:GO:0016192	P:protein folding; P:vesicle-mediated transport
GSSPFG00009357001-PA	vesicle transport protein got1b	2	P:GO:0006457; P:GO:0016192	P:protein folding; P:vesicle-mediated transport
GSSPFG00027141001-PA	v-type proton atpase catalytic subunit a	5	F:GO:0005524; P:GO:0015991; C:GO:0033180; P:GO:0046034; F:GO:0046961	F:ATP binding; P:ATP hydrolysis coupled proton transport; C:proton-transporting V-type ATPase, V1 domain; P:ATP metabolic process; F:proton-transporting ATPase activity, rotational mechanism
GSSPFG00000066001-PA	v-type proton atpase subunit e 2-like	5	P:GO:0008152; F:GO:0015078; P:GO:0015991; F:GO:0016787; C:GO:0033179	P:metabolic process; F:proton transmembrane transporter activity; P:ATP hydrolysis coupled proton transport; F:hydrolase activity; C:proton-transporting V-type ATPase, V0 domain
GSSPFG00002721001-PA	v-type proton atpase subunit e 2-like	5	P:GO:0008152; F:GO:0015078; P:GO:0015991; F:GO:0016787; C:GO:0033179	P:metabolic process; F:proton transmembrane transporter activity; P:ATP hydrolysis coupled proton transport; F:hydrolase activity; C:proton-transporting V-type ATPase, V0 domain
GSSPFG00032564001-PA	v-type proton atpase subunit g	5	C:GO:0016324; C:GO:0016471; F:GO:0042626; P:GO:0055085; P:GO:1902600	C:apical plasma membrane; C:vacuolar proton-transporting V-type ATPase complex; F:ATPase activity, coupled to transmembrane movement of substances; P:transmembrane transport; P:proton transmembrane transport
GSSPFG00017650001-PA	zinc carboxypeptidase-like	2	F:GO:0004180; F:GO:0046872	F:carboxypeptidase activity; F:metal ion binding
