# Supplementary material for: Interaction of a Densovirus with Glycans of the Peritrophic Matrix Mediates Oral Infection of the Lepidopteran Pest Spodoptera frugiperda
Source: Viruses. 2019 Sep 17;11(9):870. doi: 10.3390/v11090870 (PMC6783882; doi:10.3390/v11090870)
Supplement: Supplementary file 1 [file viruses-11-00870-s001.zip › Supplementary figures and Tables/Supp Table4_C.pdf]

Name	Description	#GO	GO IDs	GO Names
GSSPFG00022879001-PA	14-3-3 protein zeta isoform x1	2	C:GO:0005737; F:GO:0019904	C:cytoplasm; F:protein domain specific binding
GSSPFG00020046001-PA	arginine kinase	3	F:GO:0004054; F:GO:0005524; P:GO:0016310	F:arginine kinase activity; F:ATP binding; P:phosphorylation
GSSPFG00030477001.4-PA	catalase	29	F:GO:0000981; F:GO:0001158; P:GO:0003007; F:GO:0004096; F:GO:0004102; C:GO:0005634; C:GO:0005777; C:GO:0005891; P:GO:0007274; P:GO:0007517; P:GO:0008292; F:GO:0008332; P:GO:0008340; P:GO:0016339; F:GO:0020037; P:GO:0035206; P:GO:0038001; P:GO:0042542; P:GO:0042673; P:GO:0042682; P:GO:0042744; P:GO:0045471; P:GO:0045676; P:GO:0045944; F:GO:0046872; P:GO:0048813; P:GO:0055114; P:GO:0070509; P:GO:0070588	F:DNA-binding transcription factor activity, RNA polymerase II-specific; F:enhancer sequence-specific DNA binding; P:heart morphogenesis; F:catalase activity; F:choline O-acetyltransferase activity; C:nucleus; C:peroxisome; C:voltage-gated calcium channel complex; P:neuromuscular synaptic transmission; P:muscle organ development; P:acetylcholine biosynthetic process; F:low voltage-gated calcium channel activity; P:determination of adult lifespan; P:calcium-dependent cell-cell adhesion via plasma membrane cell adhesion molecules; F:heme binding; P:regulation of hemocyte proliferation; P:paracrine signaling; P:response to hydrogen peroxide; P:regulation of retinal cone cell fate specification; P:regulation of compound eye cone cell fate specification; P:hydrogen peroxide catabolic process; P:response to ethanol; P:regulation of R7 cell differentiation; P:positive regulation of transcription by RNA polymerase II; F:metal ion binding; P:dendrite morphogenesis; P:oxidation-reduction process; P:calcium ion import; P:calcium ion transmembrane transport
GSSPFG00016528001-PA	cd63 antigen-like isoform x1	1	C:GO:0016021	C:integral component of membrane
GSSPFG00034359001.3-PA	chymotrypsin-like serine protease	2	F:GO:0004252; P:GO:0006508	F:serine-type endopeptidase activity; P:proteolysis
GSSPFG00022639001.1-PA	cklf-like marvel transmembrane domain-containing protein 4	1	C:GO:0016020	C:membrane
GSSPFG00018884001.2-PA	cytochrome p450	5	F:GO:0004497; F:GO:0005506; F:GO:0016705; F:GO:0020037; P:GO:0055114	F:monooxygenase activity; F:iron ion binding; F:oxidoreductase activity, acting on paired donors, with incorporation or reduction of molecular oxygen; F:heme binding; P:oxidation-reduction process
GSSPFG00016699001-PA	cytosolic 10-formyltetrahydrofolate dehydrogenase	10	C:GO:0005737; P:GO:0006730; F:GO:0008168; P:GO:0009058; P:GO:0009258; F:GO:0016155; F:GO:0016620; F:GO:0016742; P:GO:0032259; P:GO:0055114	C:cytoplasm; P:one-carbon metabolic process; F:methyltransferase activity; P:biosynthetic process; P:10-formyltetrahydrofolate catabolic process; F:formyltetrahydrofolate dehydrogenase activity; F:oxidoreductase activity, acting on the aldehyde or oxo group of donors, NAD or NADP as acceptor; F:hydroxymethyl-, formyl- and related transferase activity; P:methylation; P:oxidation-reduction process
GSSPFG00004184001-PA	dimethyladenosine transferase mitochondrial	2	F:GO:0000179; P:GO:0031167	F:rRNA (adenine-N6,N6-)-dimethyltransferase activity; P:rRNA methylation
GSSPFG00019440001-PA	dna repair protein rad51 homolog 1	37	F:GO:0000150; P:GO:0000730; C:GO:0000800; P:GO:0001932; F:GO:0003684; F:GO:0003690; F:GO:0003697; F:GO:0005524; C:GO:0005730; C:GO:0005759; C:GO:0005815; P:GO:0006268; P:GO:0006312; P:GO:0007131; F:GO:0008022; P:GO:0009636; P:GO:0010165; P:GO:0010569; C:GO:0016605; P:GO:0032200; P:GO:0042493; F:GO:0042802; F:GO:0043142; P:GO:0046686; C:GO:0048471; P:GO:0051106; P:GO:0051260; P:GO:0051597; P:GO:0070182; P:GO:0070192; P:GO:0071480; P:GO:0072711; P:GO:0072719; P:GO:0072757; P:GO:1902521; P:GO:1990414; P:GO:1990426	F:recombinase activity; P:DNA recombinase assembly; C:lateral element; P:regulation of protein phosphorylation; F:damaged DNA binding; F:double-stranded DNA binding; F:single-stranded DNA binding; F:ATP binding; C:nucleolus; C:mitochondrial matrix; C:microtubule organizing center; P:DNA unwinding involved in DNA replication; P:mitotic recombination; P:reciprocal meiotic recombination; F:protein C-terminus binding; P:response to toxic substance; P:response to X-ray; P:regulation of double-strand break repair via homologous recombination; C:PML body; P:telomere organization; P:response to drug; F:identical protein binding; F:single-stranded DNA-dependent ATPase activity; P:response to cadmium ion; C:perinuclear region of cytoplasm; P:positive regulation of DNA ligation; P:protein homooligomerization; P:response to methylmercury; F:DNA polymerase binding; P:chromosome organization involved in meiotic cell cycle; P:cellular response to gamma radiation; P:cellular response to hydroxyurea; P:cellular response to cisplatin; P:cellular response to camptothecin; P:response to etoposide; P:replication-born double-strand break repair via sister chromatid exchange; P:mitotic recombination-dependent replication fork processing
GSSPFG00008879001.3-PA	e3 sumo-protein ligase pias3 isoform x2	3	P:GO:0008152; F:GO:0008270; F:GO:0019789	P:metabolic process; F:zinc ion binding; F:SUMO transferase activity
GSSPFG00007904001-PA	electron transfer flavoprotein-ubiquinone mitochondrial	3	F:GO:0004174; F:GO:0051536; P:GO:0055114	F:electron-transferring-flavoprotein dehydrogenase activity; F:iron-sulfur cluster binding; P:oxidation-reduction process
GSSPFG00006413001-PA	ethanolamine-phosphate cytidylyltransferase	2	P:GO:0009058; F:GO:0016779	P:biosynthetic process; F:nucleotidyltransferase activity
GSSPFG00024615001-PA	eukaryotic translation initiation factor 3 subunit b	8	F:GO:0000166; P:GO:0001731; F:GO:0003743; C:GO:0005852; P:GO:0006446; C:GO:0016282; F:GO:0031369; C:GO:0033290	F:nucleotide binding; P:formation of translation preinitiation complex; F:translation initiation factor activity; C:eukaryotic translation initiation factor 3 complex; P:regulation of translational initiation; C:eukaryotic 43S preinitiation complex; F:translation initiation factor binding; C:eukaryotic 48S preinitiation complex
GSSPFG00008242001.4-PA	glutathione s-transferase delta	2	F:GO:0004364; P:GO:0008152	F:glutathione transferase activity; P:metabolic process
GSSPFG00008243001-PA	glutathione s-transferase delta	2	F:GO:0004364; P:GO:0008152	F:glutathione transferase activity; P:metabolic process
GSSPFG00010868001-PA	h+ transporting atp synthase gamma subunit	7	C:GO:0000275; C:GO:0005811; P:GO:0006909; F:GO:0008553; P:GO:0015986; F:GO:0046933; F:GO:0046961	C:mitochondrial proton-transporting ATP synthase complex, catalytic core F(1); C:lipid droplet; P:phagocytosis; F:proton-exporting ATPase activity, phosphorylative mechanism; P:ATP synthesis coupled proton transport; F:proton-transporting ATP synthase activity, rotational mechanism; F:proton-transporting ATPase activity, rotational mechanism
GSSPFG00023356001.1-PA	importin subunit beta-1 isoform x1	5	C:GO:0005622; P:GO:0006886; P:GO:0006913; F:GO:0008536; F:GO:0008565	C:intracellular; P:intracellular protein transport; P:nucleocytoplasmic transport; F:Ran GTPase binding; F:protein transporter activity
GSSPFG00034251001-PA	intestinal mucin	3	C:GO:0005576; P:GO:0006030; F:GO:0008061	C:extracellular region; P:chitin metabolic process; F:chitin binding
GSSPFG00032730001-PA	lipase	3	C:GO:0005576; P:GO:0008152; F:GO:0052689	C:extracellular region; P:metabolic process; F:carboxylic ester hydrolase activity
GSSPFG00023038001-PA	organic cation transporter	1	F:GO:0005215	F:transporter activity
GSSPFG00032599001-PA	paired mesoderm homeobox protein 1	3	C:GO:0005634; P:GO:0006355; F:GO:0043565	C:nucleus; P:regulation of transcription, DNA-templated; F:sequence-specific DNA binding
GSSPFG00002429001-PA	pleckstrin homology domain-containing family m member 1	3	C:GO:0005622; F:GO:0008270; P:GO:0035556	C:intracellular; F:zinc ion binding; P:intracellular signal transduction
GSSPFG00004485001-PA	presequence mitochondrial	3	P:GO:0006508; F:GO:0008237; F:GO:0046872	P:proteolysis; F:metallopeptidase activity; F:metal ion binding
GSSPFG00005884001.3-PA	protein aubergine-like isoform x1	1	F:GO:0003676	F:nucleic acid binding
GSSPFG00024299001-PA	ribosomal protein l18a	3	F:GO:0003735; C:GO:0005840; P:GO:0006412	F:structural constituent of ribosome; C:ribosome; P:translation

GSSPFG00002705001.1-PA	ribosomal protein s2	4	F:GO:0003723; F:GO:0003735; P:GO:0006412; C:GO:0015935	F:RNA binding; F:structural constituent of ribosome; P:translation; C:small ribosomal subunit
GSSPFG00004056001-PA	serine protease 115 isoform x1	2	F:GO:0004252; P:GO:0006508	F:serine-type endopeptidase activity; P:proteolysis
GSSPFG00030758001-PA	serine proteinase stubble-like	1	F:GO:0008233	F:peptidase activity
GSSPFG00031520001-PA	succinate dehydrogenase assembly factor 2- mitochondrial-like	5	F:GO:0005507; C:GO:0005739; P:GO:0006121; P:GO:0018293; P:GO:0055070	F:copper ion binding; C:mitochondrion; P:mitochondrial electron transport, succinate to ubiquinone; P:protein-FAD linkage; P:copper ion homeostasis
GSSPFG00031375001-PA	thioredoxin domain-containing protein 17-like	6	F:GO:0004197; C:GO:0005764; P:GO:0006508; P:GO:0006726; P:GO:0035071; C:GO:0045169	F:cysteine-type endopeptidase activity; C:lysosome; P:proteolysis; P:eye pigment biosynthetic process; P:salivary gland cell autophagic cell death; C:fusome
GSSPFG00035519001.3-PA	trypsin-like serine proteinase t26	3	F:GO:0004252; F:GO:0005515; P:GO:0006508	F:serine-type endopeptidase activity; F:protein binding; P:proteolysis
