# Supplementary material for: Interaction of a Densovirus with Glycans of the Peritrophic Matrix Mediates Oral Infection of the Lepidopteran Pest Spodoptera frugiperda
Source: Viruses. 2019 Sep 17;11(9):870. doi: 10.3390/v11090870 (PMC6783882; doi:10.3390/v11090870)
Supplement: Supplementary file 1 [file viruses-11-00870-s001.zip › Supplementary figures and Tables/Supp Table4_D.pdf]

Name	Description	#GO	GO IDs	GO Names
GSSPFG00023167001-PA	39s ribosomal protein mitochondrial	1	C:GO:0005840	C:ribosome
GSSPFG00026398001.1-PA	39s ribosomal protein mitochondrial	4	F:GO:0003723; F:GO:0003735; C:GO:0005762; P:GO:0006412	F:RNA binding; F:structural constituent of ribosome; C:mitochondrial large ribosomal subunit; P:translation
GSSPFG00006068001-PA	40s ribosomal protein s11	3	F:GO:0003735; C:GO:0005840; P:GO:0006412	F:structural constituent of ribosome; C:ribosome; P:translation
GSSPFG00032780001.1-PA	40s ribosomal protein s13	16	P:GO:0000184; F:GO:0003729; F:GO:0003735; F:GO:0005515; C:GO:0005730; C:GO:0005925; P:GO:0006413; P:GO:0006414; P:GO:0006415; P:GO:0006614; C:GO:0016020; P:GO:0019083; C:GO:0022627; P:GO:0033119; C:GO:0070062; F:GO:0070181	P:nuclear-transcribed mRNA catabolic process, nonsense-mediated decay; F:mRNA binding; F:structural constituent of ribosome; F:protein binding; C:nucleolus; C:focal adhesion; P:translational initiation; P:translational elongation; P:translational termination; P:SRP-dependent cotranslational protein targeting to membrane; C:membrane; P:viral transcription; C:cytosolic small ribosomal subunit; P:negative regulation of RNA splicing; C:extracellular exosome; F:small ribosomal subunit rRNA binding
GSSPFG00007084001-PA	40s ribosomal protein s28	6	P:GO:0002181; F:GO:0003735; P:GO:0006355; P:GO:0006407; C:GO:0022627; C:GO:0043189	P:cytoplasmic translation; F:structural constituent of ribosome; P:regulation of transcription, DNA-templated; P:rRNA export from nucleus; C:cytosolic small ribosomal subunit; C:H4/H2A histone acetyltransferase complex
GSSPFG00013754001-PA	4-hydroxybutyrate -transferase	7	C:GO:0005739; C:GO:0005811; C:GO:0005829; P:GO:0006084; F:GO:0008411; F:GO:0016787; P:GO:0022008	C:mitochondrion; C:lipid droplet; C:cytosol; P:acetyl-CoA metabolic process; F:4-hydroxybutyrate CoA-transferase activity; F:hydrolase activity; P:neurogenesis
GSSPFG00014527001-PA	60s ribosomal protein l10a	4	F:GO:0003723; F:GO:0003735; P:GO:0006412; C:GO:0015934	F:RNA binding; F:structural constituent of ribosome; P:translation; C:large ribosomal subunit
GSSPFG00007050001-PA	60s ribosomal protein l10a	4	F:GO:0003723; F:GO:0003735; P:GO:0006412; C:GO:0015934	F:RNA binding; F:structural constituent of ribosome; P:translation; C:large ribosomal subunit
GSSPFG00011770001-PA	acetylcholine receptor subunit alpha-like isoform x2	5	C:GO:0016021; F:GO:0022848; C:GO:0030054; C:GO:0045211; P:GO:0098655	C:integral component of membrane; F:acetylcholine-gated cation-selective channel activity; C:cell junction; C:postsynaptic membrane; P:cation transmembrane transport
GSSPFG00023569001-PA	acyl-binding protein	1	F:GO:0000062	F:fatty-acyl-CoA binding
GSSPFG00016217001-PA	adenine nucleotide translocase insect2	27	P:GO:0001508; F:GO:0003676; F:GO:0005471; C:GO:0005743; C:GO:0005811; P:GO:0006839; P:GO:0007268; P:GO:0007629; P:GO:0008340; P:GO:0009612; P:GO:0010507; P:GO:0015074; P:GO:0015866; P:GO:0015867; C:GO:0016021; P:GO:0034599; P:GO:0040011; P:GO:0046716; P:GO:0048477; P:GO:0048489; P:GO:0051124; P:GO:0051480; P:GO:0051560; P:GO:0051900; P:GO:0055085; P:GO:0070050; P:GO:2001171	P:action potential; F:nucleic acid binding; F:ATP:ADP antiporter activity; C:mitochondrial inner membrane; C:lipid droplet; P:mitochondrial transport; P:chemical synaptic transmission; P:flight behavior; P:determination of adult lifespan; P:response to mechanical stimulus; P:negative regulation of autophagy; P:DNA integration; P:ADP transport; P:ATP transport; C:integral component of membrane; P:cellular response to oxidative stress; P:locomotion; P:muscle cell cellular homeostasis; P:oogenesis; P:synaptic vesicle transport; P:synaptic growth at neuromuscular junction; P:regulation of cytosolic calcium ion concentration; P:mitochondrial calcium ion homeostasis; P:regulation of mitochondrial depolarization; P:transmembrane transport; P:neuron cellular homeostasis; P:positive regulation of ATP biosynthetic process
GSSPFG00016760001-PA	aldehyde dehydrogenase family 7 member a1 homolog	5	C:GO:0016021; F:GO:0016620; F:GO:0022857; P:GO:0055085; P:GO:0055114	C:integral component of membrane; F:oxidoreductase activity, acting on the aldehyde or oxo group of donors, NAD or NADP as acceptor; F:transmembrane transporter activity; P:transmembrane transport; P:oxidation-reduction process
GSSPFG00011282001-PA	alpha-mannosyltransferase alg2	6	F:GO:0004378; C:GO:0005811; P:GO:0009058; C:GO:0012505; F:GO:0033164; P:GO:0097502	F:GDP-Man:Man1GlcNAc2-PP-Dol alpha-1,3-mannosyltransferase activity; C:lipid droplet; P:biosynthetic process; C:endomembrane system; F:glycolipid 6-alpha-mannosyltransferase activity; P:mannosylation
GSSPFG00004595001-PA	aminopeptidase n	2	F:GO:0046872; F:GO:0070011	F:metal ion binding; F:peptidase activity, acting on L-amino acid peptides
GSSPFG00007626001-PA	atp synthase subunit mitochondrial	3	C:GO:0000276; F:GO:0015078; P:GO:0015986	C:mitochondrial proton-transporting ATP synthase complex, coupling factor F(o); F:proton transmembrane transporter activity; P:ATP synthesis coupled proton transport
GSSPFG00008485001-PA	atp synthase subunit mitochondrial	3	C:GO:0000276; F:GO:0015078; P:GO:0015986	C:mitochondrial proton-transporting ATP synthase complex, coupling factor F(o); F:proton transmembrane transporter activity; P:ATP synthesis coupled proton transport
GSSPFG00033909001-PA	atp synthase subunit mitochondrial	3	C:GO:0000276; F:GO:0015078; P:GO:0015986	C:mitochondrial proton-transporting ATP synthase complex, coupling factor F(o); F:proton transmembrane transporter activity; P:ATP synthesis coupled proton transport
GSSPFG00017555001.4-PA	atp-binding cassette sub-family a member 5-like isoform x1	6	F:GO:0005215; F:GO:0005524; P:GO:0006810; P:GO:0008152; C:GO:0016021; F:GO:0016887	F:transporter activity; F:ATP binding; P:transport; P:metabolic process; C:integral component of membrane; F:ATPase activity
GSSPFG00029664001-PA	b-cell cll lymphoma 7 protein family member b	2	F:GO:0003676; F:GO:0046872	F:nucleic acid binding; F:metal ion binding
GSSPFG00031255001-PA	bess motif-containing partial	1	F:GO:0003677	F:DNA binding
GSSPFG00004769001-PA	cadherin-87a	2	P:GO:0007155; C:GO:0016020	P:cell adhesion; C:membrane
GSSPFG00001835001.3-PA	carboxyl choline esterase cce016b	2	P:GO:0008152; F:GO:0016787	P:metabolic process; F:hydrolase activity
GSSPFG00002450001-PA	carboxylesterase	2	P:GO:0008152; F:GO:0016787	P:metabolic process; F:hydrolase activity
GSSPFG00006714001-PA	catalase	13	F:GO:0004096; F:GO:0005515; P:GO:0006979; P:GO:0009987; F:GO:0020037; C:GO:0043231; P:GO:0044238; P:GO:0044260; C:GO:0044444; F:GO:0046872; P:GO:0050794; P:GO:0055114; P:GO:1901360	F:catalase activity; F:protein binding; P:response to oxidative stress; P:cellular process; F:heme binding; C:intracellular membrane-bounded organelle; P:primary metabolic process; P:cellular macromolecule metabolic process; C:cytoplasmic part; F:metal ion binding; P:regulation of cellular process; P:oxidation-reduction process; P:organic cyclic compound metabolic process
GSSPFG00008368001-PA	cg3595	29	P:GO:0000281; P:GO:0001736; P:GO:0003384; F:GO:0005509; C:GO:0005911; C:GO:0005912; C:GO:0005938; P:GO:0007298; C:GO:0016324; C:GO:0016460; C:GO:0016461; P:GO:0019749; C:GO:0030496; P:GO:0031036; C:GO:0031941; F:GO:0032036; C:GO:0032154; P:GO:0032956; P:GO:0035148; P:GO:0035159; C:GO:0035183; P:GO:0035191; P:GO:0035277; P:GO:0035317; P:GO:0042060; C:GO:0048471; C:GO:0051233; P:GO:0060288; P:GO:0090254	P:mitotic cytokinesis; P:establishment of planar polarity; P:apical constriction involved in gastrulation; F:calcium ion binding; C:cell-cell junction; C:adherens junction; C:cell cortex; P:border follicle cell migration; C:apical plasma membrane; C:myosin II complex; C:unconventional myosin complex; P:cytoskeleton-dependent cytoplasmic transport, nurse cell to oocyte; C:midbody; P:myosin II filament assembly; C:filamentous actin; F:myosin heavy chain binding; C:cleavage furrow; P:regulation of actin cytoskeleton organization; P:tube formation; P:regulation of tube length, open tracheal system; C:female germline ring canal inner rim; P:nuclear axial expansion; P:spiracle morphogenesis, open tracheal system; P:imaginal disc-derived wing hair organization; P:wound healing; C:perinuclear region of cytoplasm; C:spindle midzone; P:formation of a compartment boundary; P:cell elongation involved in imaginal disc-derived wing morphogenesis
GSSPFG00034353001.4-PA	chymotrypsin-like protease	2	F:GO:0004252; P:GO:0006508	F:serine-type endopeptidase activity; P:proteolysis

GSSPFG00034354001.3-PA	chymotrypsin-like protease	2	F:GO:0004252; P:GO:0006508	F:serine-type endopeptidase activity; P:proteolysis
GSSPFG00022970001.3-PA	chymotrypsin-like protease	2	F:GO:0004252; P:GO:0006508	F:serine-type endopeptidase activity; P:proteolysis
GSSPFG00022971001.4-PA	chymotrypsin-like protease	2	F:GO:0004252; P:GO:0006508	F:serine-type endopeptidase activity; P:proteolysis
GSSPFG00034358001.3-PA	chymotrypsin-like serine protease	2	F:GO:0004252; P:GO:0006508	F:serine-type endopeptidase activity; P:proteolysis
GSSPFG00013450001.3-PA	chymotrypsin-like serine protease precursor	2	F:GO:0004252; P:GO:0006508	F:serine-type endopeptidase activity; P:proteolysis
GSSPFG00011996001-PA	copper transport protein atox1	2	P:GO:0030001; F:GO:0046872	P:metal ion transport; F:metal ion binding
GSSPFG00014584001.1-PA	cystathionine gamma-lyase	3	P:GO:0008152; F:GO:0016829; F:GO:0030170	P:metabolic process; F:lyase activity; F:pyridoxal phosphate binding
GSSPFG00020109001.4-PB	cytochrome p450	5	F:GO:0005506; C:GO:0005789; F:GO:0020037; P:GO:0055114; F:GO:0070330	F:iron ion binding; C:endoplasmic reticulum membrane; F:heme binding; P:oxidation-reduction process; F:aromatase activity
GSSPFG00027565001.2-PA	cytochrome p450	5	F:GO:0004497; F:GO:0005506; F:GO:0016705; F:GO:0020037; P:GO:0055114	F:monooxygenase activity; F:iron ion binding; F:oxidoreductase activity, acting on paired donors, with incorporation or reduction of molecular oxygen; F:heme binding; P:oxidation-reduction process
GSSPFG00014493001.3-PB	cytochrome p450 cyp314a1	4	F:GO:0004501; F:GO:0005506; F:GO:0020037; P:GO:0055114	F:ecdysone 20-monooxygenase activity; F:iron ion binding; F:heme binding; P:oxidation-reduction process
GSSPFG00014405001-PA	dipeptidyl peptidase 4	3	P:GO:0006508; F:GO:0008236; C:GO:0016020	P:proteolysis; F:serine-type peptidase activity; C:membrane
GSSPFG00020627001-PA	dj-1 beta	5	P:GO:0008344; P:GO:0042775; P:GO:0046626; P:GO:0048640; P:GO:0070301	P:adult locomotory behavior; P:mitochondrial ATP synthesis coupled electron transport; P:regulation of insulin receptor signaling pathway; P:negative regulation of developmental growth; P:cellular response to hydrogen peroxide
GSSPFG00005701001-PA	dopamine n-acetyltransferase-like	2	F:GO:0008080; P:GO:0008152	F:N-acetyltransferase activity; P:metabolic process
GSSPFG00003027001-PA	drip protein	3	F:GO:0005215; P:GO:0006810; C:GO:0016021	F:transporter activity; P:transport; C:integral component of membrane
GSSPFG00028772001-PA	e3 ubiquitin-protein ligase mycbp2 isoform x1	2	F:GO:0004842; P:GO:0016567	F:ubiquitin-protein transferase activity; P:protein ubiquitination
GSSPFG00015967001.1-PA	e3 ubiquitin-protein ligase trip12 isoform x1	8	F:GO:0004842; F:GO:0005096; C:GO:0005622; F:GO:0008270; P:GO:0016567; F:GO:0016874; P:GO:0035556; P:GO:0043547	F:ubiquitin-protein transferase activity; F:GTPase activator activity; C:intracellular; F:zinc ion binding; P:protein ubiquitination; F:ligase activity; P:intracellular signal transduction; P:positive regulation of GTPase activity
GSSPFG00004783001.1-PA	e3 ubiquitin-protein ligase trip12 isoform x1	3	F:GO:0004842; P:GO:0016567; F:GO:0016874	F:ubiquitin-protein transferase activity; P:protein ubiquitination; F:ligase activity
GSSPFG00002850001.3-PA	epithelial membrane protein	6	F:GO:0005044; C:GO:0005764; C:GO:0005887; P:GO:0006952; P:GO:0007155; P:GO:0035071	F:scavenger receptor activity; C:lysosome; C:integral component of plasma membrane; P:defense response; P:cell adhesion; P:salivary gland cell autophagic cell death
GSSPFG00007711001-PA	eukaryotic peptide chain release factor gtp-binding subunit erf3a isoform x1	7	F:GO:0003746; F:GO:0003747; F:GO:0003924; F:GO:0005525; C:GO:0005829; P:GO:0006414; P:GO:0006415	F:translation elongation factor activity; F:translation release factor activity; F:GTPase activity; F:GTP binding; C:cytosol; P:translational elongation; P:translational termination
GSSPFG00034701001-PA	fatty acid binding protein	3	F:GO:0005215; P:GO:0006810; F:GO:0008289	F:transporter activity; P:transport; F:lipid binding
GSSPFG00034702001-PA	fatty acid binding protein	3	F:GO:0005215; P:GO:0006810; F:GO:0008289	F:transporter activity; P:transport; F:lipid binding
GSSPFG00029716001.2-PA	fatty acid binding protein	3	F:GO:0005215; P:GO:0006810; F:GO:0008289	F:transporter activity; P:transport; F:lipid binding
GSSPFG00014800001-PA	fibrillin-2-like	3	F:GO:0005201; F:GO:0005509; C:GO:0031012	F:extracellular matrix structural constituent; F:calcium ion binding; C:extracellular matrix
GSSPFG00020100001-PA	gag-pol polyprotein	1	F:GO:0016787	F:hydrolase activity
GSSPFG00028012001-PA	gdp-d-glucose phosphorylase 1	2	P:GO:0008152; F:GO:0016740	P:metabolic process; F:transferase activity
GSSPFG00026650001-PA	glucose-dependent insulinotropic receptor-like	3	F:GO:0004930; P:GO:0007186; C:GO:0016021	F:G protein-coupled receptor activity; P:G protein-coupled receptor signaling pathway; C:integral component of membrane
GSSPFG00026492001.1-PA	glucosylceramidase-like	3	P:GO:0006665; F:GO:0016787; P:GO:0050794	P:sphingolipid metabolic process; F:hydrolase activity; P:regulation of cellular process
GSSPFG00003077001.5-PA	glutathione s-transferase	2	F:GO:0004364; P:GO:0008152	F:glutathione transferase activity; P:metabolic process
GSSPFG00021491001.3-PA	growth hormone-inducible transmembrane	1	C:GO:0016021	C:integral component of membrane
GSSPFG00033217001-PA	gtp-binding protein 128up	1	F:GO:0005525	F:GTP binding
GSSPFG00000050001-PA	gtp-binding protein rad	6	F:GO:0003924; F:GO:0005525; C:GO:0005622; P:GO:0007264; C:GO:0016020; P:GO:1901386	F:GTPase activity; F:GTP binding; C:intracellular; P:small GTPase mediated signal transduction; C:membrane; P:negative regulation of voltage-gated calcium channel activity
GSSPFG00024799001-PA	guanine nucleotide exchange factor dbs-like isoform x3	1	P:GO:0050789	P:regulation of biological process
GSSPFG00015723001.3-PA	heat shock 70 kda protein 14	2	F:GO:0005524; C:GO:0005737	F:ATP binding; C:cytoplasm
GSSPFG00013616001-PA	heat shock protein 70	17	F:GO:0003723; F:GO:0005524; C:GO:0005730; C:GO:0005925; P:GO:0006457; P:GO:0006611; P:GO:0006626; P:GO:0009636; F:GO:0017134; F:GO:0031072; F:GO:0031625; C:GO:0042645; P:GO:0043066; C:GO:0043209; F:GO:0051082; C:GO:0070062; P:GO:0071347	F:RNA binding; F:ATP binding; C:nucleolus; C:focal adhesion; P:protein folding; P:protein export from nucleus; P:protein targeting to mitochondrion; P:response to toxic substance; F:fibroblast growth factor binding; F:heat shock protein binding; F:ubiquitin protein ligase binding; C:mitochondrial nucleoid; P:negative regulation of apoptotic process; C:myelin sheath; F:unfolded protein binding; C:extracellular exosome; P:cellular response to interleukin-1
GSSPFG00001676001-PA	heme oxygenase	3	F:GO:0004392; P:GO:0006788; F:GO:0046872	F:heme oxygenase (decyclizing) activity; P:heme oxidation; F:metal ion binding
GSSPFG00026171001-PA	high affinity copper uptake protein 1 isoform x2	3	F:GO:0005375; C:GO:0016021; P:GO:0035434	F:copper ion transmembrane transporter activity; C:integral component of membrane; P:copper ion transmembrane transport
GSSPFG00008731001.2-PA	hox cluster protein	5	C:GO:0005634; P:GO:0006355; P:GO:0007275; F:GO:0043565; P:GO:0048856	C:nucleus; P:regulation of transcription, DNA-templated; P:multicellular organism development; F:sequence-specific DNA binding; P:anatomical structure development
GSSPFG00007187001-PA	islet cell autoantigen 1	2	F:GO:0019904; P:GO:0046928	F:protein domain specific binding; P:regulation of neurotransmitter secretion
GSSPFG00005709001-PA	leukocyte receptor cluster member 8 homolog isoform x2	1	F:GO:0016740	F:transferase activity
GSSPFG00015310001-PA	lim and senescent cell antigen-like-containing domain protein 1 isoform x3	2	F:GO:0008270; P:GO:0046854	F:zinc ion binding; P:phosphatidylinositol phosphorylation

GSSPFG00032730001-PA	lipase	3	C:GO:0005576; P:GO:0008152; F:GO:0052689	C:extracellular region; P:metabolic process; F:carboxylic ester hydrolase activity
GSSPFG00006843001-PA	lipoyltransferase mitochondrial	2	F:GO:0003824; P:GO:0006464	F:catalytic activity; P:cellular protein modification process
GSSPFG00005448001-PA	math and lrr domain-containing protein pfe0570w-like	2	F:GO:0097159; F:GO:1901363	F:organic cyclic compound binding; F:heterocyclic compound binding
GSSPFG00029683001-PA	methionine--trna mitochondrial	3	F:GO:0000166; F:GO:0004812; P:GO:0006418	F:nucleotide binding; F:aminoacyl-tRNA ligase activity; P:tRNA aminoacylation for protein translation
GSSPFG00028152001.3-PA	mitochondrial manganese superoxide dismutase	4	F:GO:0004784; P:GO:0019430; F:GO:0046872; P:GO:0055114	F:superoxide dismutase activity; P:removal of superoxide radicals; F:metal ion binding; P:oxidation-reduction process
GSSPFG00032551001-PA	nad-dependent protein deacylase sirtuin- mitochondrial	11	C:GO:0005758; C:GO:0005759; C:GO:0005829; P:GO:0006476; F:GO:0008270; P:GO:0010566; P:GO:0036047; P:GO:0036049; F:GO:0036054; F:GO:0036055; F:GO:0070403	C:mitochondrial intermembrane space; C:mitochondrial matrix; C:cytosol; P:protein deacetylation; F:zinc ion binding; P:regulation of ketone biosynthetic process; P:peptidyl-lysine demalonylation; P:peptidyl-lysine desuccinylation; F:protein-malonyllysine demalonylase activity; F:protein-succinyllysine desuccinylase activity; F:NAD+ binding
GSSPFG00000123001.3-PA	nedd2-like caspase	3	F:GO:0004197; P:GO:0006508; P:GO:0042981	F:cysteine-type endopeptidase activity; P:proteolysis; P:regulation of apoptotic process
GSSPFG00010594001-PA	neurexin-4 isoform x2	4	F:GO:0000287; P:GO:0008152; F:GO:0008897; C:GO:0016021	F:magnesium ion binding; P:metabolic process; F:holo-[acyl-carrier-protein] synthase activity; C:integral component of membrane
GSSPFG00026778001-PA	neuronal pas domain-containing protein 4-like	7	F:GO:0003677; F:GO:0004812; F:GO:0005524; C:GO:0005634; P:GO:0006355; P:GO:0006418; F:GO:0046983	F:DNA binding; F:aminoacyl-tRNA ligase activity; F:ATP binding; C:nucleus; P:regulation of transcription, DNA-templated; P:tRNA aminoacylation for protein translation; F:protein dimerization activity
GSSPFG00025843001-PA	nuclear pore complex protein nup107	2	C:GO:0005643; P:GO:0006810	C:nuclear pore; P:transport
GSSPFG00017351001-PA	organic cation transporter	3	F:GO:0005215; C:GO:0016021; P:GO:0055085	F:transporter activity; C:integral component of membrane; P:transmembrane transport
GSSPFG00008376001-PA	pancreatic triacylglycerol lipase-like	3	C:GO:0005576; P:GO:0008152; F:GO:0052689	C:extracellular region; P:metabolic process; F:carboxylic ester hydrolase activity
GSSPFG00022738001-PA	pancreatic triacylglycerol lipase-like	3	C:GO:0005576; P:GO:0008152; F:GO:0052689	C:extracellular region; P:metabolic process; F:carboxylic ester hydrolase activity
GSSPFG00009950001-PA	pancreatic triacylglycerol lipase-like	3	C:GO:0005576; P:GO:0008152; F:GO:0052689	C:extracellular region; P:metabolic process; F:carboxylic ester hydrolase activity
GSSPFG00005108001-PA	pancreatic triacylglycerol lipase-like	3	C:GO:0005576; P:GO:0008152; F:GO:0052689	C:extracellular region; P:metabolic process; F:carboxylic ester hydrolase activity
GSSPFG00005109001-PA	pancreatic triacylglycerol lipase-like	3	C:GO:0005576; P:GO:0008152; F:GO:0052689	C:extracellular region; P:metabolic process; F:carboxylic ester hydrolase activity
GSSPFG00003126001.3-PA	partial	3	F:GO:0003700; P:GO:0006355; F:GO:0043565	F:DNA-binding transcription factor activity; P:regulation of transcription, DNA-templated; F:sequence-specific DNA binding
GSSPFG00013063001-PA	peroxisomal multifunctional enzyme type 2	4	F:GO:0004303; C:GO:0005777; C:GO:0005811; P:GO:0055114	F:estradiol 17-beta-dehydrogenase activity; C:peroxisome; C:lipid droplet; P:oxidation-reduction process
GSSPFG00026320001-PA	phosphatidate mitochondrial	4	C:GO:0005739; P:GO:0008654; C:GO:0016020; F:GO:0016779	C:mitochondrion; P:phospholipid biosynthetic process; C:membrane; F:nucleotidyltransferase activity
GSSPFG00029413001-PA	poly-specific endoribonuclease homolog	4	P:GO:0008152; F:GO:0016788; F:GO:0097159; F:GO:1901363	P:metabolic process; F:hydrolase activity, acting on ester bonds; F:organic cyclic compound binding; F:heterocyclic compound binding
GSSPFG00016195001-PA	polyubiquitin-b isoform x1	33	F:GO:0002020; F:GO:0003723; C:GO:0005615; C:GO:0005634; C:GO:0005739; P:GO:0006511; P:GO:0007141; P:GO:0007144; P:GO:0008585; P:GO:0010992; P:GO:0021888; F:GO:0031386; P:GO:0031397; P:GO:0032020; P:GO:0032649; P:GO:0034340; P:GO:0042742; C:GO:0043005; C:GO:0043025; C:GO:0043209; P:GO:0045071; P:GO:0045648; P:GO:0047497; P:GO:0048812; P:GO:0051607; P:GO:0051881; P:GO:0060613; P:GO:0061136; C:GO:0070062; P:GO:0072520; P:GO:0097009; P:GO:1901214; P:GO:1902255	F:protease binding; F:RNA binding; C:extracellular space; C:nucleus; C:mitochondrion; P:ubiquitin-dependent protein catabolic process; P:male meiosis I; P:female meiosis I; P:female gonad development; P:ubiquitin recycling; P:hypothalamus gonadotrophin-releasing hormone neuron development; F:protein tag; P:negative regulation of protein ubiquitination; P:ISG15-protein conjugation; P:regulation of interferon-gamma production; P:response to type I interferon; P:defense response to bacterium; C:neuron projection; C:neuronal cell body; C:myelin sheath; P:negative regulation of viral genome replication; P:positive regulation of erythrocyte differentiation; P:mitochondrion transport along microtubule; P:neuron projection morphogenesis; P:defense response to virus; P:regulation of mitochondrial membrane potential; P:fat pad development; P:regulation of proteasomal protein catabolic process; C:extracellular exosome; P:seminiferous tubule development; P:energy homeostasis; P:regulation of neuron death; P:positive regulation of intrinsic apoptotic signaling pathway by p53 class mediator
GSSPFG00018941001-PA	probable chitinase 3	5	F:GO:0004568; C:GO:0005576; P:GO:0005975; P:GO:0006032; F:GO:0008061	F:chitinase activity; C:extracellular region; P:carbohydrate metabolic process; P:chitin catabolic process; F:chitin binding
GSSPFG00002398001-PA	prolactin-releasing peptide receptor-like	3	F:GO:0004983; P:GO:0007218; C:GO:0016021	F:neuropeptide Y receptor activity; P:neuropeptide signaling pathway; C:integral component of membrane
GSSPFG00009775001.1-PA	prostaglandin reductase 1-like	3	F:GO:0008270; F:GO:0016491; P:GO:0055114	F:zinc ion binding; F:oxidoreductase activity; P:oxidation-reduction process
GSSPFG00009778001.1-PA	prostaglandin reductase 1-like	3	F:GO:0008270; F:GO:0016491; P:GO:0055114	F:zinc ion binding; F:oxidoreductase activity; P:oxidation-reduction process
GSSPFG00033418001-PA	protein ddi1 homolog 2	2	F:GO:0004190; P:GO:0006508	F:aspartic-type endopeptidase activity; P:proteolysis
GSSPFG00033586001-PA	protein roadkill isoform x3	12	C:GO:0005634; P:GO:0007349; P:GO:0016567; P:GO:0030162; P:GO:0031648; P:GO:0042067; P:GO:0042308; F:GO:0042803; P:GO:0043065; P:GO:0045879; P:GO:0046330; P:GO:0046331	C:nucleus; P:cellularization; P:protein ubiquitination; P:regulation of proteolysis; P:protein destabilization; P:establishment of ommatidial planar polarity; P:negative regulation of protein import into nucleus; F:protein homodimerization activity; P:positive regulation of apoptotic process; P:negative regulation of smoothened signaling pathway; P:positive regulation of JNK cascade; P:lateral inhibition
GSSPFG00007550001-PA	protein vav isoform x1	5	F:GO:0005089; C:GO:0005622; P:GO:0035023; P:GO:0043547; F:GO:0046872	F:Rho guanyl-nucleotide exchange factor activity; C:intracellular; P:regulation of Rho protein signal transduction; P:positive regulation of GTPase activity; F:metal ion binding
GSSPFG00016017001-PA	proton-coupled folate transporter-like	2	C:GO:0016021; P:GO:0055085	C:integral component of membrane; P:transmembrane transport
GSSPFG00000597001-PA	rab gdp dissociation inhibitor alpha	8	F:GO:0005093; C:GO:0005829; C:GO:0005875; P:GO:0007269; C:GO:0008021; P:GO:0015031; P:GO:0016192; P:GO:0050790	F:Rab GDP-dissociation inhibitor activity; C:cytosol; C:microtubule associated complex; P:neurotransmitter secretion; C:synaptic vesicle; P:protein transport; P:vesicle-mediated transport; P:regulation of catalytic activity
GSSPFG00000598001-PA	rab gdp-dissociation inhibitor	3	F:GO:0005093; P:GO:0015031; P:GO:0050790	F:Rab GDP-dissociation inhibitor activity; P:protein transport; P:regulation of catalytic activity
GSSPFG00017853001-PA	seminal fluid protein hacp038	2	F:GO:0004252; P:GO:0006508	F:serine-type endopeptidase activity; P:proteolysis

GSSPFG00018308001-PA	septin isoform b	15	P:GO:0000281; F:GO:0003746; F:GO:0003924; F:GO:0005525; C:GO:0005875; C:GO:0005940; P:GO:0006414; P:GO:0007295; P:GO:0007349; P:GO:0007444; P:GO:0022008; C:GO:0031105; F:GO:0042803; C:GO:0045172; P:GO:0051726	P:mitotic cytokinesis; F:translation elongation factor activity; F:GTPase activity; F:GTP binding; C:microtubule associated complex; C:septin ring; P:translational elongation; P:growth of a germarium-derived egg chamber; P:cellularization; P:imaginal disc development; P:neurogenesis; C:septin complex; F:protein homodimerization activity; C:germline ring canal; P:regulation of cell cycle
GSSPFG00023708001.3-PA	serine threonine-protein kinase pelle	4	F:GO:0004674; F:GO:0005524; P:GO:0006468; P:GO:0007165	F:protein serine/threonine kinase activity; F:ATP binding; P:protein phosphorylation; P:signal transduction
GSSPFG00022767001-PA	set1 ash2 histone methyltransferase complex subunit ash2	3	F:GO:0008168; F:GO:0008270; P:GO:0032259	F:methyltransferase activity; F:zinc ion binding; P:methylation
GSSPFG00029484001-PA	signal-induced proliferation-associated 1-like protein 2 isoform x2	3	F:GO:0005096; P:GO:0043547; P:GO:0051056	F:GTPase activator activity; P:positive regulation of GTPase activity; P:regulation of small GTPase mediated signal transduction
GSSPFG00002106001.3-PA	slit homolog 1 protein	5	P:GO:0007165; P:GO:0008152; C:GO:0016021; F:GO:0016787; P:GO:0045087	P:signal transduction; P:metabolic process; C:integral component of membrane; F:hydrolase activity; P:innate immune response
GSSPFG00029082001.6-PA	small heat shock protein	2	F:GO:0005212; C:GO:0005840	F:structural constituent of eye lens; C:ribosome
GSSPFG00031268001-PA	splicing factor 3b subunit 6-like protein	2	F:GO:0000166; F:GO:0003676	F:nucleotide binding; F:nucleic acid binding
GSSPFG00018170001-PA	sterol carrier protein 2 3-oxoacyl- thiolase	7	F:GO:0005548; C:GO:0005739; C:GO:0005777; C:GO:0005811; P:GO:0008152; P:GO:0015914; F:GO:0016747	F:phospholipid transporter activity; C:mitochondrion; C:peroxisome; C:lipid droplet; P:metabolic process; P:phospholipid transport; F:transferase activity, transferring acyl groups other than amino-acyl groups
GSSPFG00007548001-PA	sulfiredoxin-1 isoform x1	3	F:GO:0005524; F:GO:0032542; P:GO:0055114	F:ATP binding; F:sulfiredoxin activity; P:oxidation-reduction process
GSSPFG00013592001.3-PA	superoxide dismutase	5	F:GO:0004784; C:GO:0005737; P:GO:0019430; F:GO:0046872; P:GO:0055114	F:superoxide dismutase activity; C:cytoplasm; P:removal of superoxide radicals; F:metal ion binding; P:oxidation-reduction process
GSSPFG00011227001-PA	synaptic vesicle glycoprotein 2b	4	F:GO:0008270; C:GO:0016021; F:GO:0022857; P:GO:0055085	F:zinc ion binding; C:integral component of membrane; F:transmembrane transporter activity; P:transmembrane transport
GSSPFG00026383001-PA	taf6-like rna polymerase ii p300 cbp-associated factor-associated factor 65 kda	5	C:GO:0005634; P:GO:0006325; P:GO:0006352; F:GO:0046982; P:GO:1903506	C:nucleus; P:chromatin organization; P:DNA-templated transcription, initiation; F:protein heterodimerization activity; P:regulation of nucleic acid-templated transcription
GSSPFG00017370001-PA	telomerase reverse transcriptase	2	F:GO:0003964; P:GO:0006278	F:RNA-directed DNA polymerase activity; P:RNA-dependent DNA biosynthetic process
GSSPFG00001034001-PA	topoisomerase partial	5	F:GO:0003677; F:GO:0003917; F:GO:0003918; C:GO:0005694; P:GO:0006265	F:DNA binding; F:DNA topoisomerase type I activity; F:DNA topoisomerase type II (ATP-hydrolyzing) activity; C:chromosome; P:DNA topological change
GSSPFG00015808001-PA	transcription factor dp-1	6	F:GO:0003677; F:GO:0003700; C:GO:0005634; C:GO:0005667; P:GO:0006355; P:GO:0007049	F:DNA binding; F:DNA-binding transcription factor activity; C:nucleus; C:transcription factor complex; P:regulation of transcription, DNA-templated; P:cell cycle
GSSPFG00021500001.3-PA	transforming growth factor beta-1-induced transcript 1 protein	1	F:GO:0008270	F:zinc ion binding
GSSPFG00024425001.3-PA	transforming growth factor beta-1-induced transcript 1 protein	1	F:GO:0008270	F:zinc ion binding
GSSPFG00012665001-PA	translocon-associated protein subunit gamma	3	C:GO:0005784; P:GO:0006613; C:GO:0030176	C:Sec61 translocon complex; P:cotranslational protein targeting to membrane; C:integral component of endoplasmic reticulum membrane
GSSPFG00024304001-PA	transmembrane protein 14c	1	C:GO:0016021	C:integral component of membrane
GSSPFG00001387001.3-PA	trypsin-like protease	1	F:GO:0016787	F:hydrolase activity
GSSPFG00030048001.3-PA	trypsin-like serine protease	2	F:GO:0004252; P:GO:0006508	F:serine-type endopeptidase activity; P:proteolysis
GSSPFG00028489001.5-PA	trypsin-like serine proteinase t26	2	F:GO:0005515; F:GO:0008233	F:protein binding; F:peptidase activity
GSSPFG00026411001-PA	tyrosine aminotransferase	4	F:GO:0004838; P:GO:0009058; P:GO:0009072; F:GO:0030170	F:L-tyrosine:2-oxoglutarate aminotransferase activity; P:biosynthetic process; P:aromatic amino acid family metabolic process; F:pyridoxal phosphate binding
GSSPFG00004237001-PA	u7 snrna-associated sm-like protein lsm10	1	C:GO:0005634	C:nucleus
GSSPFG00023353001-PA	uncharacterized protein loc101456014	8	P:GO:0002121; F:GO:0005089; P:GO:0007411; P:GO:0007480; P:GO:0035025; P:GO:0035277; P:GO:0043547; C:GO:0045179	P:inter-male aggressive behavior; F:Rho guanyl-nucleotide exchange factor activity; P:axon guidance; P:imaginal disc-derived leg morphogenesis; P:positive regulation of Rho protein signal transduction; P:spiracle morphogenesis, open tracheal system; P:positive regulation of GTPase activity; C:apical cortex
GSSPFG00007846001-PA	uncharacterized protein loc105389112	3	F:GO:0016301; P:GO:0016310; F:GO:0043168	F:kinase activity; P:phosphorylation; F:anion binding
GSSPFG00000520001-PA	uncharacterized protein partial	2	F:GO:0005319; P:GO:0006869	F:lipid transporter activity; P:lipid transport
GSSPFG00025800001-PA	uv excision repair protein rad23 homolog b	4	F:GO:0003684; C:GO:0005634; P:GO:0006289; P:GO:0043161	F:damaged DNA binding; C:nucleus; P:nucleotide-excision repair; P:proteasome-mediated ubiquitin-dependent protein catabolic process
GSSPFG00034417001-PA	uv opsin	7	F:GO:0004930; P:GO:0007186; P:GO:0007601; P:GO:0007602; F:GO:0009881; C:GO:0016021; P:GO:0018298	F:G protein-coupled receptor activity; P:G protein-coupled receptor signaling pathway; P:visual perception; P:phototransduction; F:photoreceptor activity; C:integral component of membrane; P:protein-chromophore linkage
GSSPFG00014231001-PA	v-type proton atpase subunit e	4	P:GO:0008152; P:GO:0015991; C:GO:0033178; F:GO:0046961	P:metabolic process; P:ATP hydrolysis coupled proton transport; C:proton-transporting two-sector ATPase complex, catalytic domain; F:proton-transporting ATPase activity, rotational mechanism
GSSPFG00009112001-PA	zinc finger protein 271-like	1	F:GO:0005488	F:binding
GSSPFG00000957001-PA	zinc finger protein 800 isoform x2	1	F:GO:0005488	F:binding
