# Supplementary material for: Interaction of a Densovirus with Glycans of the Peritrophic Matrix Mediates Oral Infection of the Lepidopteran Pest Spodoptera frugiperda
Source: Viruses. 2019 Sep 17;11(9):870. doi: 10.3390/v11090870 (PMC6783882; doi:10.3390/v11090870)
Supplement: Supplementary file 1 [file viruses-11-00870-s001.zip › Supplementary figures and Tables/Supplementary legends.docx]

**SUPPLEMENTARY FIGURES AND TABLES LEGENDS**

**Supplementary Figure 1. Calcofluor increases *S. frugiperda* caterpillars susceptibility to densovirus oral infection.** Survival of *S. frugiperda* caterpillars following JcDV infection. Larvae (n=24) were infected individually by feeding with JcDV (10^9^ veg/caterpillar) concomitantly with 0.5% (5 μg) of Calcofluor or PBS as a control. Caterpillar mortality was recorded once a day during 10 days and results were presented as survival rates per day. Three independent experiments were performed, each independent experiment gave similar results, two are represented here (Replicates 2 and 3, R2 and R3). The log-rank (Mantel-cox) and the Gehan-Breslow-Wilcoxon tests were used to determine statistical significance. *P* values of less than 0.05 were considered significant (ns, non significant; *, *P* < 0.05; **, *P* < 0.01). ‘PBS’ refers to control (PBS-treated and non-infected) caterpillars; ‘Calcofluor’ refers to Calcofluor-treated and non-infected caterpillars; ‘JcDV’ refers to JcDV-infected caterpillars and ‘JcDV+Calcofluor’ to Calcofluor-treated and JcDV-infected caterpillars.

**Supplementary Figure 2. Virus interaction with monosaccharides did not interfere with anti-capsid antibody recognition.** Dot blots assays were performed with semi-purified (A) or purified (B) JcDV. Virus (10^9^ veg/µl) were incubated or not with 5 mM of glycans (GlcNAc, GalNAc, Fucose or Mannose) for 1 h before dilution (1:10 and 1:100) and deposit of a drop onto nitrocellulose membranes. Control assays were performed with non-infected homogenate of caterpillars (Mock for JcDV, in A) or with PBS (for P_JcDV, in B). The membranes were saturated with 5% low-fat dry milk powder in PBST for 1 h at RT, and incubated OVN at 4°C with a rabbit anti-capsid antibody (1:1,000), then incubated 1 h with an anti-rabbit secondary antibody HRP-conjugated (see Methods). Dots were revealed by enhanced chemiluminescence using a Chemidoc imager (Biorad). Two independent experiments were performed, each independent experiment gave similar results, one is represented here.

**Supplementary Figure 3. Replicates of survival curves of caterpillars infected by JcDV.** Affinity for glycans mediates JcDV oral infection of *S. frugiperda* caterpillars. **(A and B)** Survival curves of caterpillars (n=24) infected by feeding with JcDV alone or with JcDV (10^9^ veg/caterpillar) incubated for 1 h with 5 µM (A) or 5 mM (B) of glycans (GlcNAc, GalNAc, Fucose or Mannose) before infection. Control caterpillars were fed with PBS. **(C)** Survival curves of caterpillars (n=24) infected by injection with JcDV alone or with JcDV (10^9^ veg/caterpillar) incubated for 1 h with 5 mM of glycans (GlcNAc, GalNAc, Fucose or Mannose) before infection. Control caterpillars were injected with PBS. Three independent experiments were performed, each independent experiment gave similar results, two are represented here (Replicates 2 and 3, R2 and R3). The log-rank (Mantel-cox) and the Gehan-Breslow-Wilcoxon tests were used to determine statistical significance. *P* values of less than 0.05 were considered significant (ns, non-significant; *, *P* < 0.05; **, *P* < 0.01; *** *P* < 0.001). ‘PBS’ refers to control (PBS-treated and non-infected) caterpillars; ‘JcDV’ to JcDV-infected caterpillars; ‘JcDV+GlcNAc’, ‘JcDV+GalNAc’, ‘JcDV+Fucose’ and ‘JcDV+Mannose’ refer to caterpillars infected with JcDV incubated with GlcNAc, GalNAc, Fucose or Mannose, respectively, before infection.

**Supplementary Figure 4. JcDV oral infection induces midgut gene expression modulation.** Pie charts representation of GO assignment of 196 and 60 unique transcripts over- and under-represented (≥ 5 fold change) between infected and non-infected midgut DGE libraries, respectively, at day 1 (up panel) and day 3 (lower panel) p.i.. Categorization were based on GO terms assignment from 2^nd^ level of Biological Process (A) and Molecular Function (B) using Blast2GO software.

**Supplementary Table 1. Annotation of PM proteins interacting with JcDV in VOPBA.** Protein bands revealed by VOPBA were cut in SDS-PAGE gel stained with Page blue and analyzed by LC–MS/MS as described in Methods. Among the 155 proteins common to the three replicates, 138 were annotated in the reference genome of *S. frugiperda* (Gouin et al., 2017). Sequences which found homology were annotated according to the gene ontology (GO) terms and classified using Blast2Go software (<https://www.blast2go.com/>). Column 1 contains the identifiers of the predicted transcripts from the OGS2.2 genome (Gouin et al., 2017). Columns 4 and 5 contain the corresponding GO IDs (GO identifiers) and GO names, respectively; P (biological process), F (molecular function) or C (cellular component) refers to the ontology to which the GO IDs belong.

**Supplementary Table 2. Characteristics of DGE libraries generated from caterpillars orally infected with JcDV.**

**Supplementary Table 3. Characteristics of annotated Tags and transcripts.** Differential expression of the Tag counts of the infected *versus* mock conditions was performed to obtain a list of up- and down-represented tags for each condition. Tags for which differential expression was ≥ 5 fold change were assigned using the reference databases for *S. frugiperda* (Legai et al., 2014; Gouin et al., 2017).

**Supplementary Table 4. Annotation of regulated intestinal transcripts following oral JcDV infection.** GO assignment of 60 and 196 unique transcripts down- and over-represented at day 1 (Tables 4A and B) and day 3 (Tables 4C and D) p.i. using Blast2Go software (<https://www.blast2go.com/>). Columns 1 contain the identifiers of the predicted transcripts from the OGS2.2 genome (Gouin et al., 2017). Columns 4 and 5 contain the corresponding GO IDs (GO identifiers) and GO names, respectively; P (biological process), F (molecular function) or C (cellular component) refers to the ontology to which the GO IDs belong.
