# Supplementary figures and images for: Interaction of a Densovirus with Glycans of the Peritrophic Matrix Mediates Oral Infection of the Lepidopteran Pest Spodoptera frugiperda
Source: Viruses. 2019 Sep 17;11(9):870. doi: 10.3390/v11090870 (PMC6783882; doi:10.3390/v11090870)

**A**

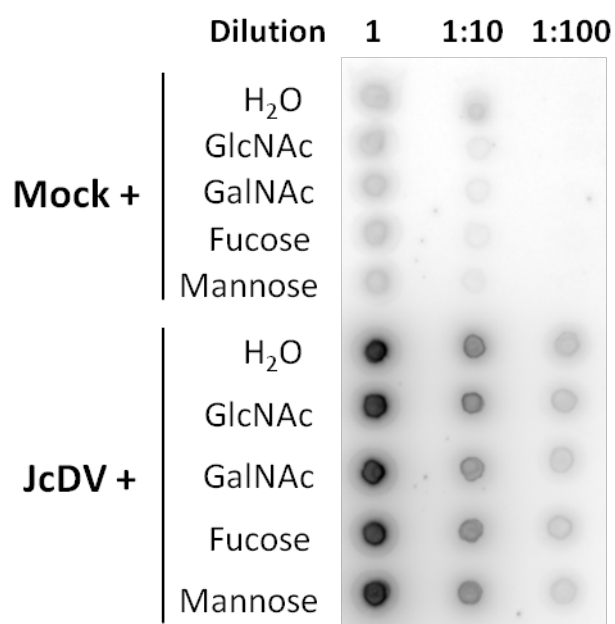

**B**

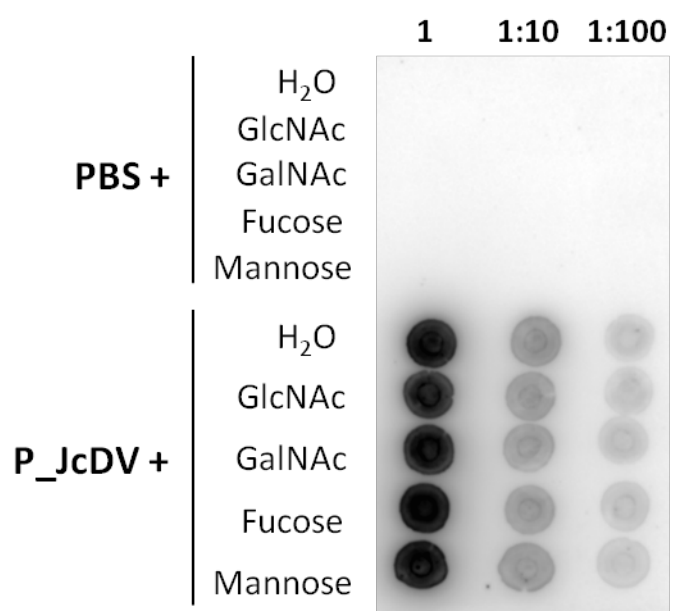

Supplement: Supplementary file 1 [file viruses-11-00870-s001.zip › Supplementary figures and Tables/Supp Fig2.pdf]

**C**

Infection by injection

— PBS

— JcDV

— GlcNAc

— GalNAc

— Fucose

— Mannose

+ JcDV

5 mM (R2)

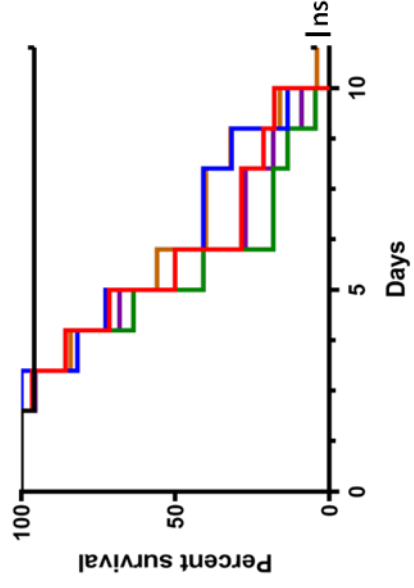

5 mM (R3)

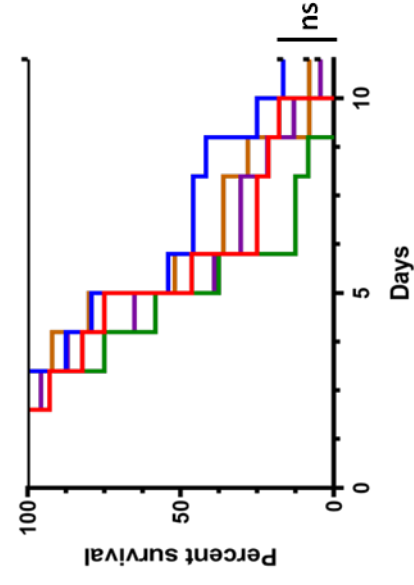

Supplement: Supplementary file 1 [file viruses-11-00870-s001.zip › Supplementary figures and Tables/Supp Fig3_C.pdf]
